# Novel Anti-Melanogenic Compounds, (*Z*)-5-(Substituted Benzylidene)-4-thioxothiazolidin-2-one Derivatives: In Vitro and In Silico Insights

**DOI:** 10.3390/molecules26164963

**Published:** 2021-08-17

**Authors:** Heejeong Choi, Il Young Ryu, Inkyu Choi, Sultan Ullah, Hee Jin Jung, Yujin Park, Yeongmu Jeong, YeJi Hwang, Sojeong Hong, In-Soo Yoon, Hwayoung Yun, Min-Soo Kim, Jin-Wook Yoo, Yunjin Jung, Pusoon Chun, Hyung Ryong Moon

**Affiliations:** 1 Laboratory of Medicinal Chemistry, College of Pharmacy, Pusan National University, Busan 46241, Korea; heejeong@pusan.ac.kr (H.C.); iy102@pusan.ac.kr (I.Y.R.); godhot785@pusan.ac.kr (I.C.); hjjung2046@pusan.ac.kr (H.J.J.); pyj10@pusan.ac.kr (Y.P.); dassabn@pusan.ac.kr (Y.J.); yjw4238@pusan.ac.kr (Y.H.); wwjd0912@pusan.ac.kr (S.H.); insoo.yoon@pusan.ac.kr (I.-S.Y.); hyun@pusan.ac.kr (H.Y.); minsookim@pusan.ac.kr (M.-S.K.); jinwook@pusan.ac.kr (J.-W.Y.); jungy@pusan.ac.kr (Y.J.); 2 Department of Molecular Medicine, The Scripps Research Institute, Jupiter, FL 33458, USA; sultanwardag@gmail.com; 3 College of Pharmacy, Inje University, Gyeongnam, Gimhae 50834, Korea; 4 Inje Institute of Pharmaceutical Sciences and Research, Inje University, Gyeongnam, Gimhae 50834, Korea

**Keywords:** tyrosinase, PUSTC scaffold, anti-melanogenic effect, docking simulation

## Abstract

To confirm that the β-phenyl-α,β-unsaturated thiocarbonyl (PUSTC) scaffold, similar to the β-phenyl-α,β-unsaturated carbonyl (PUSC) scaffold, acts as a core inhibitory structure for tyrosinase, twelve (*Z*)-5-(substituted benzylidene)-4-thioxothiazolidin-2-one ((*Z*)-BTTZ) derivatives were designed and synthesized. Seven of the twelve derivatives showed stronger inhibitory activity than kojic acid against mushroom tyrosinase. Compound **2b** (IC_50_ = 0.47 ± 0.97 µM) exerted a 141-fold higher inhibitory potency than kojic acid. Kinetic studies’ results confirmed that compounds **2b** and **2f** are competitive tyrosinase inhibitors, which was supported by high binding affinities with the active site of tyrosinase by docking simulation. Docking results using a human tyrosinase homology model indicated that **2b** and **2f** might potently inhibit human tyrosinase. In vitro assays of **2b** and **2f** were conducted using B16F10 melanoma cells. Compounds **2b** and **2f** significantly and concentration-dependently inhibited intracellular melanin contents, and the anti-melanogenic effects of **2b** at 10 µM and **2f** at 25 µM were considerably greater than the inhibitory effect of kojic acid at 25 µM. Compounds **2b** and **2f** similarly inhibited cellular tyrosinase activity and melanin contents, indicating that the anti-melanogenic effects of both were due to tyrosinase inhibition. A strong binding affinity with the active site of tyrosinase and potent inhibitions of mushroom tyrosinase, cellular tyrosinase activity, and melanin generation in B16F10 cells indicates the PUSTC scaffold offers an attractive platform for the development of novel tyrosinase inhibitors.

## 1. Introduction

Skin whitening and depigmentation are widespread practices in some ethnic groups, especially in Asia, Africa, and the Middle East, because of the complex interplay between cultural, social, political, and psychological factors [1], and a lighter complexion is considered a beauty attribute in these regions. About 28% of the world’s population undergo skin whitening at least once [2], and according to a market study, the use of skin whitening agents could reach USD 13.7 billion by 2025 [3]. In addition, depigmentation agents are also used to treat skin disorders caused by hyperpigmentation, such as melasma, lentigo, erythormelanosis follicularis faciei et colli, congenital melanocytic naevi, erythema dyschromicum perstans, and post-inflammatory hyperpigmentation [4]. Hyperpigmentation is the result of the excessive production of melanin, which is synthesized by melanosomes by a process called melanogenesis, which starts with the conversion of L-tyrosine to L-dopa by tyrosinase. This process occurs in special dendritic cells called melanocytes located in human skin, hair, and eyes, and at the subcellular level, is confined to unique membrane-bound organelles called melanosomes. Melanin is an essential component of the epidermis and protects skin from UV radiation and free radicals, but abnormal levels of melanin are associated with many pathologies. Tyrosinase plays a vital role in melanogenesis, as it catalyzes the rate-limiting first two stages of the process. Thus, most efforts to suppress or reduce melanogenesis in vivo have focused on the identification of tyrosinase inhibitors. 

Hydroquinone, kojic acid, arbutin, azelaic acid, and resveratrol are the most commonly used skin whitening agents for cosmetic and dermatological purposes. However, these agents all raise efficacy and safety concerns. For example, hydroquinone, which is considered a gold standard and has been used for decades, can cause skin irritation, exogenous ochronosis, contact dermatitis, conjunctival melanosis, corneal degeneration, and nail discoloration when used long-term [5,6,7,8] and has toxic effects on bone marrow, the kidneys, and the immune system [9]. In addition, the hydroquinone metabolite 1,4-benzoquinone appears to be carcinogenic, which has led to a ban on the use of hydroquinone in the EU [10]. Kojic acid has storage stability issues and may be carcinogenic [11], and ellagic acid also suffers from low bioavailability problems [11]. Due to these limitations of existing tyrosinase inhibitors, researchers in academia and industry are trying to develop new, potent, safe tyrosinase inhibitors from natural and synthetic sources. 

**Figure 1:**
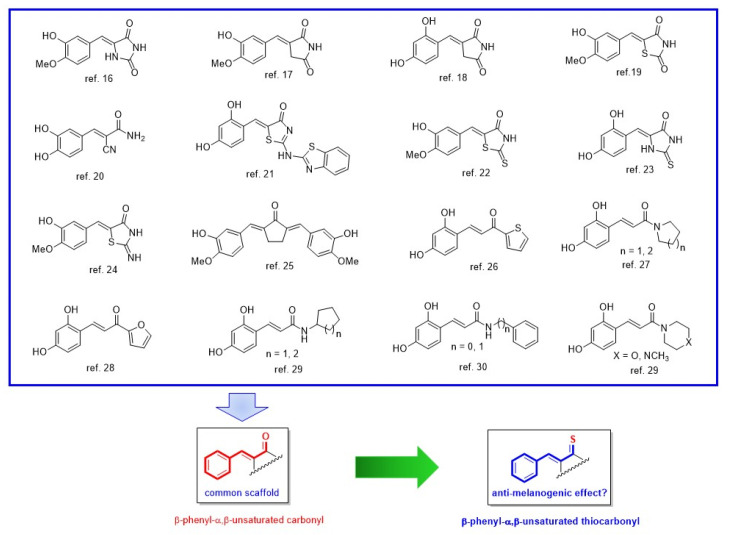
Potent tyrosinase inhibitors with a β-phenyl-α,β-unsaturated carbonyl (PUSC) scaffold and a diagram showing the relationship between the PUSC and PUSTC scaffolds.

Several tyrosinase inhibitor scaffolds have been studied over the past few decades, but none of them are used clinically [12,13,14,15]. During our past studies, we synthesized various derivatives with a phenyl-α,β-unsaturated carbonyl (PUSC) scaffold [16,17,18,19,20,21,22,23,24,25,26,27,28,29,30] with the aim of developing novel, potent tyrosinase inhibitors (Figure 1). In B16F10 melanoma cells, all of these derivatives inhibited tyrosinase and melanogenic activity more potently than kojic acid, a representative tyrosinase inhibitor. The present study was undertaken to determine whether replacing the PUSC scaffold with a β-phenyl-α,β-unsaturated thiocarbonyl (PUSTC) scaffold might enhance tyrosinase inhibitory and anti-melanogenic activities (Figure 1). As 4-thioxothiazolidin-2-one has been found in many drug-like molecules [31,32,33] used as anticancer, antibacterial, anti-inflammatory, antiviral, and antibiotics [34,35,36], 4-thioxothiazolidin-2-one was considered a great template to build the PUSTC scaffold. Therefore, twelve (*Z*)-5-(substituted benzylidene)-4-thioxothiazolidin-2-one ((*Z*)-BTTZ) derivatives having the 4-thioxothiazolidin-2-one structure were synthesized and evaluated for mushroom tyrosinase, B16F10 cellular tyrosinase, and melanogenesis inhibitory activities using cell-based assays. In addition, kinetic studies were conducted using Lineweaver–Burk double reciprocal plots to determine their inhibitory modes of action, and a docking simulation was performed using a homology model of human tyrosinase to investigate human tyrosinase inhibitory potentials. 

## 2. Results and Discussion

### 2.1. Chemistry 

**Scheme 1:**
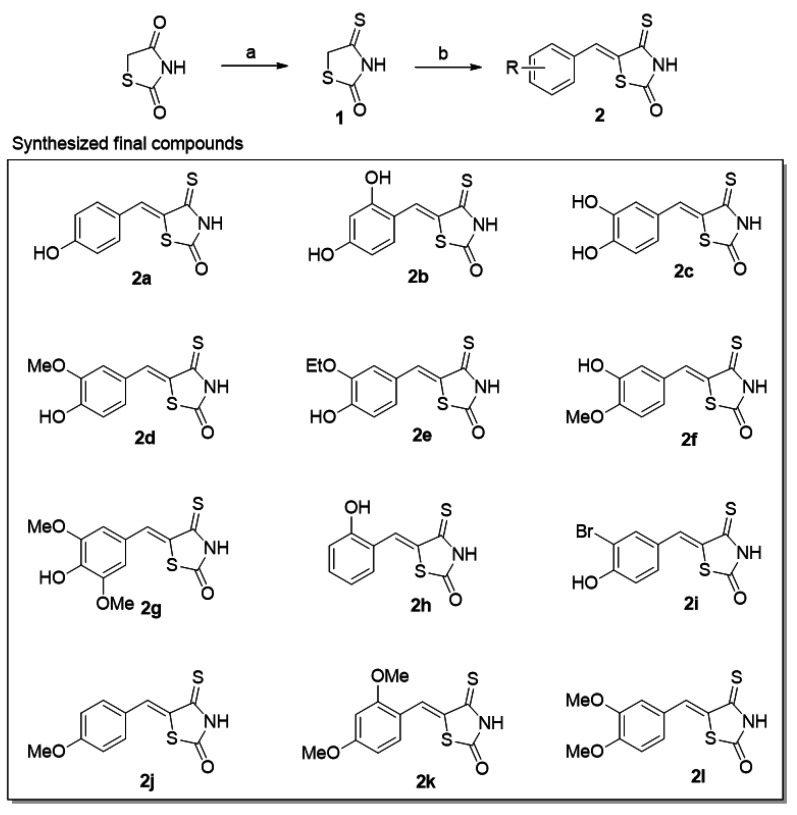
Synthetic scheme for (*Z*)-BTTZ derivatives. Reagents and conditions: (**a**) Lawesson’s reagent, toluene, reflux, 1 h, 90%; (**b**) substituted benzaldehydes, piperidine, ethanol, reflux, 15–30 min, 35–82%.

**Figure 2:**
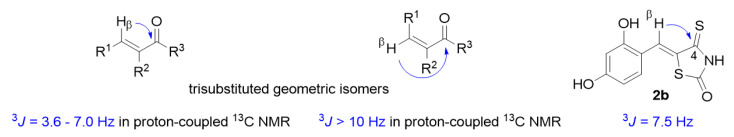
Relationship between the geometry of the double bond and C,H-spin-coupling constants over three bonds and the ^3^*J* value at C(4) of compound **2b**.

First, 4-thioxothiazolidin-2-one (**1**) was prepared as a key intermediate for the synthesis of the desired (*Z*)-BTTZ derivatives **2a**–**2l** by reacting thiazolidin-2,4-dione with Lawesson’s reagent (2,4-bis(4-methoxyphenyl)-2,4-dithioxo-1,3,2,4-dithiadiphosphetane) (90% yield, Scheme 1). The synthesis of the final compounds **2a**–**2l** was achieved using Knoevenagel condensation [37]. The coupling of compound **1** with substituted benzaldehydes in the presence of piperidine in ethanol afforded the twelve (*Z*)-BTTZ derivatives **2a**–**2l** as sole products at yields from 35 to 82%. To examine the effect of substituents on the β-phenyl ring of PUSTC on tyrosinase inhibition, benzaldehydes bearing a hydroxyl, methoxyl, bromo, and/or an ethoxyl group were coupled with 4-thioxothiazolidin-2-one (**1**). Twelve (*Z*)-BTTZ derivatives with the PUSTC scaffold were synthesized and identified using ^1^H and ^13^C NMR and mass spectroscopy. The geometries of the double bond generated by benzaldehyde coupling with compound **1** were determined using vicinal ^1^H and ^13^C-coupling constants (^3^*J*) in proton-coupled ^13^C NMR spectra. Nair and co-workers reported that in the proton-coupled ^13^C NMR spectra of a variety of trisubstituted geometric isomers, which were α,β-unsaturated carbonyl compounds including 5- or 6-membered exocyclic methylene carbonyl compounds, different vicinal ^1^H,^13^C-coupling constants (^3^*J*) were observed (Figure 2) [38]. In geometric isomers with a cis positioned carbonyl and β-hydrogen, the carbonyl carbon had vicinal ^1^H,^13^C-coupling constants (^3^*J*) in the range 4.8 to 7.0 Hz, whereas in the trans position, the carbonyl carbon had vicinal ^1^H, ^13^C-coupling constants above 10 Hz. As shown in Figure 2, the ^13^C NMR of compound **2b** was measured in the proton-coupled ^13^C mode, and the vicinal ^1^H,^13^C-coupling constant (^3^*J*) of C4 was 7.5 Hz (Supplementary data, S6), indicating that compound **2b** was the (*Z*)-isomer. Furthermore, the ^1^H and ^13^C NMR data of compound **2b** matched that of the authentic compound prepared by reacting compound **1** with 2,4-dihydroxybenzaldehyde in the presence of sodium acetate and acetic acid [37]. 

### 2.2. Mushroom Tyrosinase Inhibition and Values of Logarithm of Partition Function

Although it has been reported that melanogenesis is regulated by hormones [39], tyrosinase inhibition is the most promising method of inhibiting melanogenesis. L-Tyrosine and L-dopa are not only substrates for melanogenesis, but also act as regulators of the melanogenesis pathway [40]. The anti-tyrosinase efficacies of the 12 synthesized (*Z*)-BTTZ derivatives **2a**–**2l** were examined as previously described in a study on mushroom tyrosinase inhibitory activity using kojic acid as a positive control and L-tyrosine as a substrate [39]. All 12 (*Z*)-BTTZ derivatives exhibited concentration-dependent mushroom tyrosinase inhibition. The IC_50_ values of compounds **2a**–**2l** and kojic acid are shown in Table 1.

**Figure 3:**
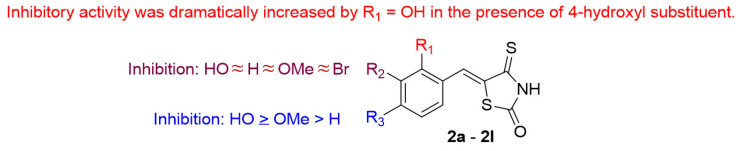
Structure–activity relationships of (*Z*)-BTTZ derivatives **2a**–**2l**.

Compound **2b** most potently inhibited mushroom tyrosinase with an IC_50_ value of 0.47 ± 0.97 µM, whereas the representative tyrosinase inhibitor, kojic acid, had an IC_50_ value of 66.30 ± 0.75 µM. That is, compound **2b** with two hydroxyls at the two and four positions on the β-phenyl ring of the PUSTC scaffold was 141 times more potent than kojic acid. Compound **2f**, which had a 3-hydroxy and a 4-methoxyl substituent on the β-phenyl ring also exhibited strong tyrosinase inhibition with an IC_50_ value of 15.24 ± 1.68 µM. Interestingly, compound **2d** (IC_50_: 70.75 ± 1.00 µM), which had a methoxyl at the three position and a hydroxyl at the four position had a 4.6-fold lower mushroom tyrosinase inhibitory activity than compound **2f**. In addition to compounds **2b** and **2f**, five other compounds, namely, **2a** with a 4-hydroxyphenyl, **2c** with a 3,4-dihydroxyphenyl, **2i** with a 3-bromo-4-hydroxyl, **2j** with a 4-methoxyl, and **2l** with a 3,4-dimethoxyl, also inhibited mushroom tyrosinase more potently than kojic acid, with IC_50_ values of 28.05 ± 1.16, 47.41 ± 2.18, 26.27 ± 4.10, 30.83 ± 1.41, and 23.31 ± 0.28 µM, respectively; that is, these five compounds had a 1.4- to 2.8-fold stronger tyrosinase inhibitory activity than kojic acid. Compound **2e**, in which the 3-methoxyl substituent of **2d** was replaced with the more sterically demanding ethoxyl substituent, had slightly less tyrosinase inhibitory activity than kojic acid (IC_50_: 70.75 ± 1.00 µM to 92.81 ± 0.89 µM). Compound **2k**, in which the 2,4-dihydroxyl substituents of compound **2b** were replaced with 2,4-dimethoxyl, showed a dramatic reduction in tyrosinase inhibitory activity (from an IC_50_ of 0.47 ± 0.97 µM to 126.35 ± 0.46 µM). Compound **2b** with a 2,4-dihydroxyl substituent had the lowest IC_50_ value, but compounds **2a** and **2h** in which the 2- or 4-hydroxyl groups of compound **2b** were removed, respectively, had a 60- and 314-fold lower tyrosinase inhibitory activities than **2b**. These results indicate that the 2,4-dihydroxyl substitution on the β-phenyl ring of the PUSTC scaffold markedly increases tyrosinase inhibition. The insertion of a 3-bromo group into the β-phenyl ring of compound **2a** did not affect mushroom tyrosinase inhibition (**2a** vs. **2i**). In summary, hydroxyl and methoxyl groups at R_3_ conferred greater tyrosinase inhibitory activity than hydrogen, and generally, the presence of a hydroxyl group at R_3_ resulted in stronger tyrosinase inhibition than a methoxyl (Figure 3). At R_2_, all the substituents, including hydrogen and bromine, exhibited similar tyrosinase inhibitory activity and the presence of hydroxyl at R_2_ resulted in higher tyrosinase inhibitory activity in the presence of a 4-methoxyl group (**2f** vs. **2j**). The hydroxyl substituent at R_1_ greatly increased the tyrosinase inhibitory activity in the presence of the 4-hydroxyl substituent. These results suggest that the PUSTC scaffold might be an important template for mushroom tyrosinase inhibitors. 

Log *p* values for the 12 (*Z*)-BTTZ derivatives **2a**–**2l** were obtained using ChemDraw Ultra 12.0 and the observed range was 1.26 to 2.47 (Table 1). The log *p* value of kojic acid was −2.45, which indicated that the (*Z*)-BTTZ derivatives are more likely to be absorbed by skin than kojic acid.

Due to their strong inhibitory activities against mushroom tyrosinase, compounds **2b** and **2f** were used in docking simulation and kinetic studies and in in vitro experiments on their tyrosinase inhibitory and anti-melanogenic activities.

### 2.3. Modes of Action of Compounds ***2b*** and ***2f***

**Figure 4:**
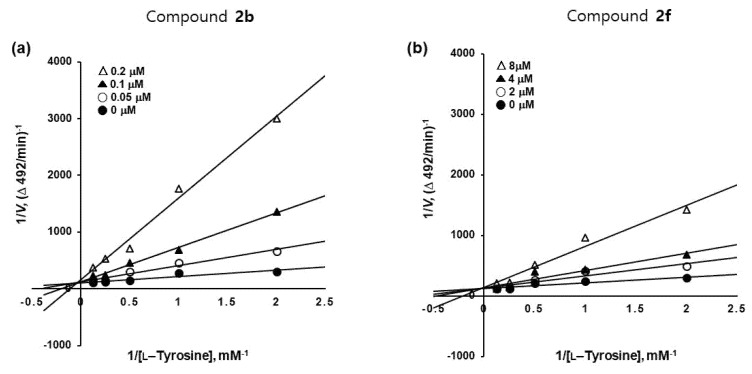
Lineweaver–Burk plots for the inhibition of mushroom tyrosinase by compounds (**a**) **2b** and (**b**) **2f**. Modes of action of **2b** and **2f** were determined using Lineweaver–Burk plots. Results are presented as mean values of 1/V, defined as the inverse of increased dopachrome absorbance per min at 492 nm as determined by three independent experiments at five different L-tyrosine concentrations. For **2b**, four different inhibitor concentrations were used, 0 µM (filled circles), 0.05 µM (unfilled circles), 0.1 µM (filled triangles), and 0.2 µM (unfilled triangles), and for **2f**, four different inhibitor concentrations were used, 0 µM (filled circles), 2.0 µM (unfilled circles), 4.0 µM (filled triangles), and 8.0 µM (unfilled triangles). The modified Michaelis–Menten equation was used to calculate 1/V_max_ values (1/V_max_ = (1/K_M_)(1 + [S]/K_i_), where V_max_ is maximum reaction rate, K_M_ is a Michaelis–Menten constant, [S] is L-tyrosine concentration, and K_i_ is the inhibition constant). All experiments were independently performed three times.

To investigate the modes of action of compounds **2b** and **2f**, a kinetic study was performed using mushroom tyrosinase and different concentrations of L-tyrosine as a substrate in the presence of **2b** or **2f**. As depicted in Figure 4, inhibitory mechanisms were determined using Lineweaver–Burk plot analysis. The Lineweaver–Burk plot patterns of **2b** and **2f** were similar. The plots obtained at different concentrations of **2b** and **2f** merged at single points on the y-axis. Regardless of the concentration, the V_max_ values of **2b** and **2f** were independent of concentration, whereas their K_M_ values increased concentration-dependently. These results show that compounds **2b** and **2f** are competitive inhibitors of mushroom tyrosinase.

### 2.4. In Silico Studies of (Z)-BTTZ Derivatives ***2b*** and ***2f***

In the mushroom tyrosinase enzyme assays, compounds **2b** and **2f** potently inhibited tyrosinase and kinetic studies indicated that they inhibited it competitively, which suggests they both bind strongly to the active site of mushroom tyrosinase. In silico studies were performed using Schrödinger Suite (release 2021-1) to determine whether tyrosinase inhibition was caused by direct binding between **2b** or **2f** and the active site of tyrosinase. Docking simulations of **2b** and **2f** were performed using the crystal structure of mushroom tyrosinase and a human tyrosinase homology model based on human tyrosinase-related protein (*h*TRP1). The results were compared with kojic acid. 

#### 2.4.1. Docking Simulations of Compounds **2b** and **2f** and Kojic Acid with Mushroom Tyrosinase 

**Figure 5:**
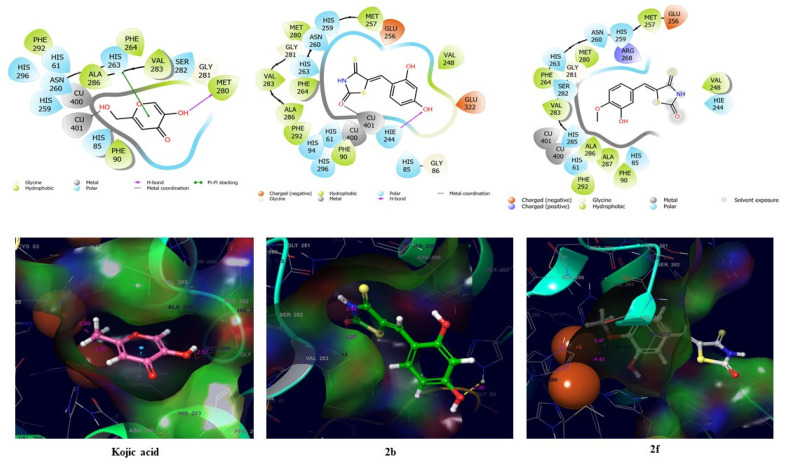
Docking studies on compounds **2b** and **2f** and kojic acid using Schrödinger Suite. *m*TYR (*Agaricus bisporus* tyrosinase, PDB ID = 2Y9X) was used as the tyrosinase for docking simulations. Pharmacophore results for **2b**, **2f**, and kojic acid are represented in 2D and 3D conformations.

The crystal structure of mushroom tyrosinase (PDB ID = 2Y9X) was imported from the protein data bank (PDB) and prepared for docking against **2b**, **2f**, and kojic acid. The 2D and 3D conformations shown in Figure 5 indicate binding interactions between compounds **2b** and **2f** and the active site of mushroom tyrosinase. As shown by the figure, both compounds occupied the same binding site as kojic acid, although the interactions involved differed. Interestingly, similar to kojic acid, compound **2b** coordinated with the copper ion (Cu401) of tyrosinase using the carbonyl of its thioxothiazolidin-2-one ring. Compound **2b** hydrogen bonded (2.39 Å) with Hie244 (protonated His244) instead of Met280. On the other hand, compound **2f** did not exhibit metal coordination, but the methoxyl group of its phenyl ring interacted (3.37 and 4.42 Å) with both copper ions of tyrosinase. Interestingly, **2f** and **2b** adopted different conformations at the active site of mushroom tyrosinase. The phenyl ring of **2b** was located far from the copper ions, whereas the phenyl ring of **2f** was located close to the copper ions. These interactions resulted in docking scores of −5.12 and −4.56 kcal/mol for **2b** and **2f**, respectively, and of −4.42 kcal/mol for kojic acid. These binding affinity scores implied that **2b** and **2f** inhibit mushroom tyrosinase activity more potently than kojic acid and support that both are competitive inhibitors. Furthermore, **2b** and **2f** had higher binding affinities than kojic acid, which suggested that the PUSTC scaffold, similar to the PUSC scaffold, confers potent tyrosinase inhibitory activity.

#### 2.4.2. Docking Simulation of Compounds **2b** and **2f** and Kojic Acid with the Human Tyrosinase Homology Model 

To further investigate the inhibitory activities of compounds **2b** and **2f**, we used a human tyrosinase homology model to confirm that these compounds effectively interact with human tyrosinase. For this purpose, we created a human tyrosinase homology model using Schrödinger Suite based on human tyrosinase related protein (*h*TRP1, PDB ID = 5M8Q) [41,42]. 

**Figure 6:**
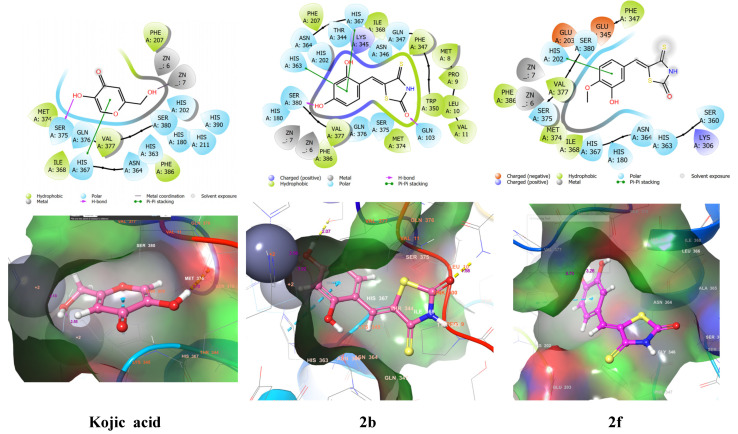
Expected binding modes of compounds **2b**, **2f,** and kojic acid in the *h*TYR homology model using Schrödinger Suite. The 2D and 3D conformations of **2b**, **2f**, and kojic acid are shown, respectively.

The binding interactions between compounds **2b**, **2f**, and kojic acid and the human tyrosinase homology model are shown in Figure 6 in 2D and 3D conformations. Kojic acid has been shown to coordinate with a zinc ion (Zn7) of tyrosinase, to form a hydrogen bond at Ser375, and to interact by pi–pi stacking with His367. Compound **2b** was unable to coordinate with either of the two zinc ions of tyrosinase but formed two hydrogen bonds and interacted by pi–pi stacking. Compound **2b** used one of its two hydroxyl groups on its β-phenyl ring to hydrogen bond with Ser380, and the carbonyl group of the thiooxothiazolidin-2-one ring to hydrogen bond with Gln103. Its pi–pi stacking interaction was formed by the β-phenyl ring and two amino acids, His363 and His367. The lipophilic region of compound **2b** was located in a hydrophobic environment created by several amino acid residues (marked green in Figure 6). On the other hand, compound **2f** formed only one pi–pi stacking interaction with His202. According to these results, the calculated docking scores for compounds **2b** and **2f** and kojic acid were −4.98, −4.05, and −4.56 kcal/mol, respectively.

Compound **2b** had a higher binding affinity than compound **2f** or kojic acid with both *m*TYR and *h*TYR in the docking simulations. Furthermore, **2b** and **2f** occupied the same binding sites in *m*TYR and *h*TYR as kojic acid (Figure 5 and Figure 6), but their interactions in the binding sites differed. The thiooxothiazolidin-2-one ring of compound **2b** and the phenyl ring of compound **2f** were placed near metal ions (Cu^++^ or Zn^++^, respectively) in the active sites of *m*TYR and *h*TYR. Both compounds had similar or higher binding affinities than kojic acid in docking simulations with *m*TYR and *h*TYR. These results indicate that the PUSTC scaffold is well tolerated at the active sites of *m*TYR and *h*TYR.

### 2.5. Cytotoxic Effects of Compounds ***2b*** and ***2f***

**Figure 7:**
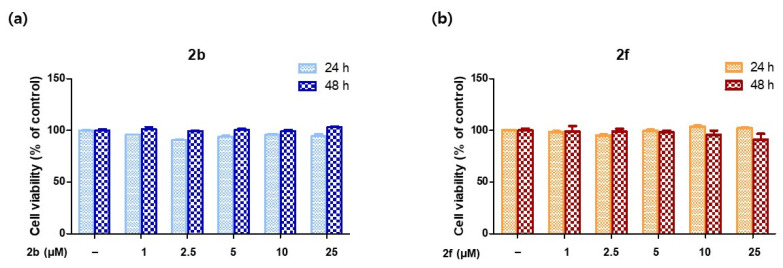
The cytotoxic effects of compounds **2b** (**a**) and **2f** (**b**) on B16F10 melanoma cells at concentrations of 1, 2.5, 5, 10, or 25 µM. Cell viabilities were determined using the EZ-Cytox assay and are expressed as percentages versus non-treated controls. Bars present standard errors.

Before examining the inhibitory effects of compounds **2b** and **2f** on tyrosinase and melanin biosynthesis, we investigated their cytotoxic effects using murine B16F10 melanoma cells. The cells were treated with different concentrations of **2b** or **2f** (0, 1, 2.5, 5, 10, or 25 µM) for 24 and 48 h (Figure 7), respectively, and the cell viabilities were assessed using the EZ-Cytox assay. Neither **2b** nor **2f** exhibited any significant cytotoxic effect at concentrations of ≤25 µM.

### 2.6. Anti-Melanogenic Activities of Compounds ***2b*** and ***2f***

Melanin content assays were used to evaluate the inhibitory effects of compounds **2b** and **2f** on α-MSH-stimulated melanogenesis. Briefly, B16F10 melanoma cells were pretreated with various concentrations (0, 5, 10, or 25 µM) of compounds **2b** or **2f** or kojic acid (25 µM) for 1 h, and then stimulated with α-MSH (0.5 µM for **2b**, and 1.0 µM for **2f**) for 48 h to increase the melanin contents. The inhibitory activities on α-MSH-stimulated melanogenesis were determined by measuring melanin optical densities. 

**Figure 8:**
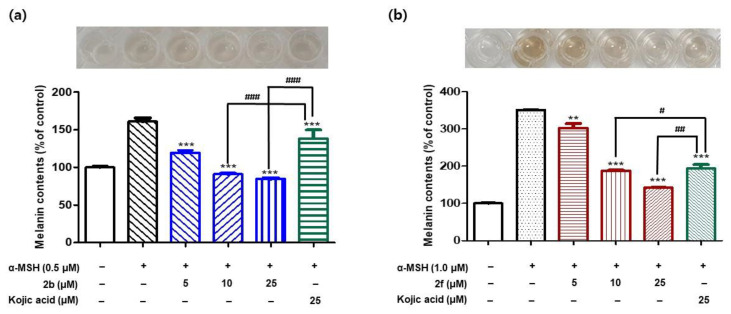
The effects of compounds **2b** (**a**) and **2f** (**b**) on melanin levels in B16F10 melanoma cells stimulated with α-MSH (0.5 µM for **2b**, and 1.0 µM for **2f**). Kojic acid was used as the positive control. B16F10 cells were treated with 5, 10, or 25 µM of **2b** or **2f**, or 25 µM of kojic acid. Symbols * and ^#^ indicate significant differences between columns: ** *p* < 0.01, and *** *p* < 0.001 for α-MSH-stimulated cells versus α-MSH-stimulated and compound (**2b**, **2f**, or kojic acid) treated cells; ^#^ *p* < 0.05, ^##^ *p* < 0.01, and ^###^ *p* < 0.001 for compounds **2b** and **2f** versus kojic acid. Bars represent standard errors.

When the α-MSH-stimulated cells were treated with **2b** at 5, 10, or 25 µM, melanin levels decreased from 1.61-fold for α-MSH-stimulated cells to 1.19-, 0.91-, and 0.85-fold, respectively (values are percentages of non-treated controls). On the other hand, kojic acid at 25 µM only decreased melanin levels to 1.38-fold. Furthermore, **2b** at 5 µM more potently inhibited melanin production than kojic acid at 25 µM. When the α-MSH-stimulated melanoma cells were treated with **2b** at different concentrations, the reductions in melanin contents were as follows: 26% at 5 µM, 44% at 10 µM, and 47% at 25 µM, which is compared with 14% for kojic acid at 25 µM (Figure 8a). 

When the α-MSH-stimulated cells were treated with **2f** at 5, 10, or 25 µM, melanin levels reduced from 3.51-fold for α-MSH (1 µM) cells to 3.03-, 1.88-, and 1.42-fold, respectively (values are percentages of non-treated controls). Compound **2f** concentration-dependently reduced melanogenesis, and at 25 µM, inhibited melanogenesis activity more than kojic acid (25 µM). When the α-MSH-stimulated melanoma cells were treated with **2f,** the reductions in melanin contents were 14% at 5 µM, 46% at 10 µM, and 60% at 25 µM, which compared with 45% for kojic acid at 25 µM (Figure 8b). At 10 µM, compound **2f** inhibited melanin production to the same extent as kojic acid at 25 µM.

### 2.7. Anti-Tyrosinase Activities of Compounds ***2b*** and ***2f***

The inhibitory activities of compounds **2b** and **2f** against cellular tyrosinase were assessed using α-MSH-stimulated melanoma cells. B16F10 cells were exposed to various concentrations (0, 5, 10, or 25 µM) of compounds **2b** or **2f** or kojic acid (25 µM) for 1 h, and then stimulated with α-MSH (0.5 µM for **2b**, and 1.0 µM for **2f**) for 48 h to increase tyrosinase activity. The inhibitory effects on tyrosinase activity were evaluated by measuring optical densities. 

**Figure 9:**
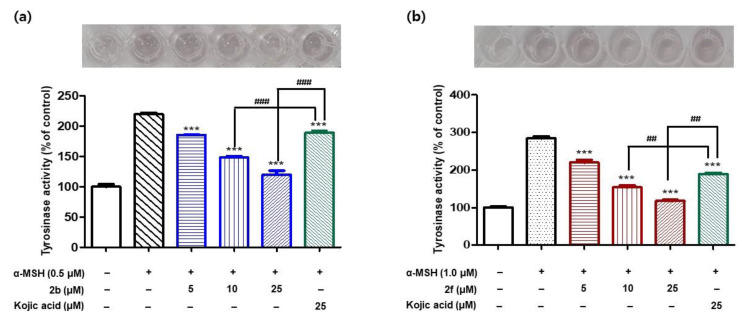
The inhibitory effects of compounds **2b** (**a**) and **2f** (**b**) on tyrosinase in B16F10 melanoma cells stimulated with α-MSH (0.5 µM for **2b** and 1.0 µM for **2f**). Kojic acid was used as a positive control. B16F10 cells were treated with 5, 10, or 25 µM of **2b** or **2f** or 25 µM of kojic acid. Symbols * and ^#^ indicate significant differences between columns: *** *p* < 0.001 for α-MSH stimulated controls versus α-MSH stimulated and **2b**, **2f**, or kojic acid treated cells; ^##^ *p* < 0.01 and ^###^ *p* < 0.001 for compounds **2b** and **2f**, respectively, versus kojic acid. Bars represent standard errors.

For compound **2b**, exposure at 5, 10, or 25 µM resulted in concentration-dependent reductions in tyrosinase activity from 2.19-fold for cells stimulated with α-MSH (versus non-treated cells) to 1.85-, 1.48-, and 1.20-fold, respectively, which corresponded to reductions of 16, 32, and 45%, as compared with 14% for 25 µM kojic acid (Figure 9a). Notably, at 5 µM, **2b** had the same inhibitory effect as kojic acid at 25 µM. 

For compound **2f**, exposure at 5, 10, or 25 µM resulted in concentration-dependent reductions in tyrosinase activity from 2.84-fold for cells stimulated with α-MSH (versus non-treated cells) to 2.20-, 1.54-, and 1.18-fold, respectively, which corresponded to reductions of 23, 46, and 59%, as compared with 33% for kojic acid at 25 µM. (Figure 9b). 

As neither compound **2b** nor **2f** had a significant cytotoxic effect on B16F10 melanoma cells at ≤25 µM, we considered that the anti-melanogenic activities of compounds **2b** and **2f** (Figure 8) were caused by tyrosinase inhibition. 

## 3. Conclusions

To investigate the tyrosinase inhibitory effects of the PUSTC scaffold, we synthesized 12 (*Z*)-BTTZ derivatives (compounds **2a** to **2l**) with this scaffold. Seven of the derivatives inhibited mushroom tyrosinase more potently than kojic acid, and compound **2b** had an IC_50_ value of only 0.47 ± 0.97 µM, which made it >100 times more potent than kojic acid. The Lineweaver–Burk plots of compounds **2b** and **2f** indicated they both competitively inhibited mushroom tyrosinase, and the docking simulation results showed both bound strongly to the active sites of human and mushroom tyrosinase. Compounds **2b** and **2f** inhibited tyrosinase activity concentration-dependently in α-MSH-stimulated B16F10 murine melanoma cells, and did so much more effectively than kojic acid. Melanin production was also concentration-dependently reduced by **2b** and **2f** in α-MSH-stimulated melanoma cells, and again both were markedly more potent than kojic acid. The similarity between the inhibitions of cellular tyrosinase and melanogenesis by compounds **2b** and **2f** and their minimal cytotoxic effects at ≤25 µM suggested that their anti-melanogenic effects were due to the inhibition of cellular tyrosinase. These results suggest that the PUSTC scaffold offers a template for the design of tyrosinase inhibitors. 

## 4. Materials and Methods 

### 4.1. Chemistry

#### 4.1.1. General Methods

All reagents were obtained commercially and used without further purification. Reactions were carried out under nitrogen and monitored using thin layer chromatography (TLC, Merck precoated 60F_245_ plates). Column chromatography was conducted using MP Silica 40–63, 60 Å. All anhydrous solvents were distilled over Na/benzophenone or CaH_2_. Low resolution mass data were recorded in ESI positive mode on an Expression CMS mass spectrometer (Advion Inc., Ithaca, NY, USA). ^1^H and ^13^C NMR spectra were obtained using a Varian Unity INOVA 400 or a Varian Unity AS500 unit (Agilent technologies, Santa Clara, CA, USA) at 400 or 500 MHz for ^1^H NMR and 100 or 125 MHz for ^13^C NMR. CD_3_OD, CDCl_3_, and DMSO-*d*_6_ were used as NMR solvents. All chemical shifts were measured in parts per million (ppm) versus residual solvent or deuterated peaks *δ*_H_ 7.26 and *δ*_C_ 77.0 for CDCl_3_, *δ*_H_ 3.31 for CD_3_OD, *δ*_H_ 2.48 and *δ*_C_ 40.0 for DMSO-*d*_6_). Coupling constant (*J*) values are presented in hertz (Hz). The following abbreviations are used for ^1^H NMR: s (singlet), d (doublet), t (triplet), q (quartet), brs (broad singlet), and dd (doublet of doublets).

##### Synthesis of 4-thioxothiazolidin-2-one (**1**) 

To a stirred solution of thiazolidin-2,4-dione (7.0 g, 59.76 mmol) in toluene (60 mL) was added 2,4-bis(4-methoxyphenyl)-2,4-dithioxo-1,3,2,4-dithiadiphosphetane (Lawesson’s reagent, 12.0 g, 29.67 mmol). The reaction mixture was refluxed for 1 h, cooled to room temperature, and filtered. The precipitate thus obtained was washed with hexane to give 4-thioxothiazolidin-2-one (**1**, 7.18 g, 90%) as a solid. ^1^H NMR (400 MHz, DMSO-*d*_6_) *δ* 4.57 (s, 2H, 5-H_2_); ^13^C NMR (125 MHz, DMSO-*d*_6_) *δ* 207.2 (*C*=S), 176.4 (*C*=O), 46.4 (*C*H_2_); LRMS (ESI-) *m*/*z* 132 (M − H)^−^.

##### General Procedure Used to Synthesize (Z)-5-(Substituted Benzylidene)-4-thioxothiazolidin-2-one Derivatives (Compounds **2a**–**2l**)

Piperidine (0.3 equivalent) was added to a stirred solution of a substituted benzaldehyde (100 mg) and 5,6-dihydroimidazo [2,1-*b*]thiazol-3(2*H*)-one (1.0 equivalent) in ethanol (1.0 mL) and refluxed for 15 to 60 min. Water was then added to the reaction mixture and stirred overnight. The precipitate obtained by filtration was washed with water, MeOH, DCM, ethyl acetate, and/or acetone (depending on the solubilities of residual starting materials and by-products) to give pure (*Z*)-5-(substituted benzylidene)-4-thioxothiazolidin-2-one derivatives **2a**–**2l** as solids in yields of 35–82%.

##### (Z)-5-(4-Hydroxybenzylidene)-4-thioxothiazolidin-2-one (Compound **2a**)

Reaction time, 30 min; yield, 49%; ^1^H NMR (500 MHz, DMSO-*d*_6_) *δ* 13.66 (brs, 1H, NH), 10.51 (s, 1H, OH), 8.02 (s, 1H, vinylic H), 7.54 (d, 2H, *J* = 8.5 Hz, 2-H, 6-H), 6.92 (d, 2H, *J* = 8.5 Hz, 3-H, 5-H); ^13^C NMR (125 MHz, DMSO-*d*_6_) *δ* 195.4, 171.1, 161.2, 137.4, 133.8, 126.3, 124.9, 117.1; LRMS (ESI-) *m*/*z* 236 (M − H)^−^.

##### (Z)-5-(2,4-Dihydroxybenzylidene)-4-thioxothiazolidin-2-one (Compound **2b**)

Reaction time, 35 min; yield, 71%; ^1^H NMR (500 MHz, CD_3_OD) *δ* 8.62 (s, 1H, vinylic H), 7.32 (d, 1H, *J* = 8.5 Hz, 6-H), 6.40 (dd, 1H, *J* = 8.5, 2.0 Hz, 5-H), 6.36 (d, 1H, *J* = 2.0 Hz, 3-H); ^13^C NMR (100 MHz, DMSO-*d*_6_) *δ* 195.2, 171.5, 163.4, 161.6, 133.4, 130.8, 124.5, 113.5, 109.6, 103.2; LRMS (ESI-) *m*/*z* 252 (M − H)^−^.

##### (Z)-5-(3,4-Dihydroxybenzylidene)-4-thioxothiazolidin-2-one (Compound **2c**)

Reaction time, 15 min; yield, 82%; ^1^H NMR (500 MHz, DMSO-*d*_6_) *δ* 13.63 (brs, 1H, NH), 10.07 (brs, 1H, OH), 9.55 (s, 1H, OH), 7.94 (s, 1H, vinylic H), 7.06* (s, 1H, 2-H), 7.06* (d, 1H, *J* = 8.5 Hz, 6-H), 6.87 (d, 1H, *J* = 8.5 Hz, 5-H); ^13^C NMR (125 MHz, DMSO-*d*_6_) *δ* 195.5, 171.3, 150.2, 146.5, 137.8, 126.1, 125.9, 125.3, 117.0, 117.0; LRMS (ESI-) *m*/*z* 252 (M − H)^−^.

##### (Z)-5-(4-Hydroxy-3-methoxybenzylidene)-4-thioxothiazolidin-2-one (compound **2d**)

Reaction time, 15 min; yield, 53%; ^1^H NMR (500 MHz, DMSO-*d*_6_) *δ* 13.67 (brs, 1H, NH), 10.18 (s, 1H, OH), 8.04 (s, 1H, vinylic H), 7.22 (s, 1H, 2-H), 7.17 (d, 1H, *J* = 8.0 Hz, 6-H), 6.93 (d, 1H, *J* = 8.0 Hz, 5-H), 3.82 (s, 3H, OCH_3_); ^13^C NMR (100 MHz, DMSO-*d*_6_) *δ* 195.6, 171.4, 151.1, 148.8, 138.0, 126.7, 125.8, 125.6, 117.1, 115.4, 56.3; LRMS (ESI-) *m*/*z* 266 (M − H)^−^.

##### (Z)-5-(3-Ethoxy-4-hydroxybenzylidene)-4-thioxothiazolidin-2-one (Compound **2e**)

Reaction time, 15 min; yield, 72%; ^1^H NMR (500 MHz, CDCl_3_) *δ* 9.57 (s, 1H, NH), 8.11 (s, 1H, vinylic H), 7.18 (d, 1H, *J* = 8.5 Hz, 6-H), 7.06 (s, 1H, 2-H), 7.02 (d, 1H, *J* = 8.5 Hz, 5-H), 6.16 (brs, 1H, OH), 4.19 (q, 2H, *J* = 7.0 Hz, C*H*_2_CH_3_), 1.51 (t, 3H, *J* = 7.0 Hz, CH_2_C*H*_3_); ^13^C NMR (100 MHz, DMSO-*d*_6_) *δ* 193.3, 169.7, 149.3, 146.5, 138.8, 126.5, 126.5, 126.5, 115.6, 113.4, 65.0, 14.9; LRMS (ESI-) *m*/*z* 280 (M − H)^−^.

##### (Z)-5-(3-Hydroxy-4-methoxybenzylidene)-4-thioxothiazolidin-2-one (Compound **2f**)

Reaction time, 15 min; yield, 61%; ^1^H NMR (500 MHz, DMSO-*d*_6_) *δ* 13.70 (brs, 1H, NH), 9.58 (s, 1H, OH), 7.96 (s, 1H, vinylic H), 7.17 (d, 1H, *J* = 8.5 Hz, 6-H), 7.08* (s, 1H, 2-H), 7.07* (d, 1H, *J* = 8.5 Hz, 5-H), 3.83 (s, 3H, OCH_3_); ^13^C NMR (100 MHz, DMSO-*d*_6_) *δ* 195.7, 171.4, 151.5, 147.8, 137.5, 127.4, 126.8, 125.6, 116.6, 113.3, 56.4; LRMS (ESI-) *m*/*z* 266 (M − H)^−^.

##### (Z)-5-(4-Hydroxy-3,5-dimethoxybenzylidene)-4-thioxothiazolidin-2-one (Compound **2g**)

Reaction time, 20 min; yield, 47%; ^1^H NMR (400 MHz, DMSO-*d*_6_) *δ* 9.53 (brs, 1H, OH), 8.00 (s, 1H, vinylic H), 6.93 (s, 2H, 2-H, 6-H), 3.79 (s, 6H, 2 × OCH_3_); ^13^C NMR (100 MHz, DMSO-*d*_6_) *δ* 196.4, 171.9, 148.9, 140.1, 137.7, 127.8, 124.6, 109.3, 56.8; LRMS (ESI-) *m*/*z* 296 (M − H)^−^.

##### (Z)-5-(2-Hydroxybenzylidene)-4-thioxothiazolidin-2-one (Compound **2h**)

Reaction time, 20 min; yield, 78%; ^1^H NMR (500 MHz, DMSO-*d*_6_) *δ* 13.72 (brs, 1H, NH), 10.60 (s, 1H, OH), 8.44 (s, 1H, vinylic H), 7.40 (d, 1H, *J* = 8.0 Hz, 6-H), 7.32 (t, 1H, *J* = 8.0 Hz, 4-H), 6.96* (d, 1H, *J* = 8.0 Hz, 3-H), 6.93* (t, 1H, *J* = 8.0 Hz, 5-H); ^13^C NMR (100 MHz, DMSO-*d*_6_) *δ* 196.9, 171.9, 158.9, 133.5, 132.0, 129.5, 128.9, 121.4, 120.5, 116.9; LRMS (ESI-) *m*/*z* 236 (M − H)^−^.

##### (Z)-5-(3-Bromo-4-hydroxybenzylidene)-4-thioxothiazolidin-2-one (Compound **2i**)

Reaction time, 1 h; yield, 73%; ^1^H NMR (500 MHz, DMSO-*d*_6_) *δ* 13.72 (brs, 1H, NH), 11.32 (brs, 1H, OH), 7.98 (s, 1H, vinylic H), 7.85 (s, 1H, 2-H), 7.53 (d, 1H, *J* = 8.5 Hz, 6-H), 7.09 (d, 1H, *J* = 8.5 Hz, 5-H); ^13^C NMR (100 MHz, DMSO-*d*_6_) *δ* 195.7, 171.1, 157.6, 136.7, 135.8, 132.0, 128.0, 126.8, 117.7, 111.1; LRMS (ESI-) *m*/*z* 314 (M − H)^−^, 316 (M − H + 2)^−^.

##### (Z)-5-(4-Methoxybenzylidene)-4-thioxothiazolidin-2-one (Compound **2j**)

Reaction time, 15 min; yield, 47%; ^1^H NMR (500 MHz, DMSO-*d*_6_) *δ* 13.70 (brs, 1H, NH), 8.05 (s, 1H, vinylic H), 7.62 (d, 2H, *J* = 8.5 Hz, 2-H, 6-H), 7.09 (d, 2H, *J* = 8.5 Hz, 3-H, 5-H), 3.82 (s, 3H, OCH_3_); ^13^C NMR (100 MHz, DMSO-*d*_6_) *δ* 196.0, 171.4, 162.2, 136.9, 133.5, 127.8, 126.7, 115.8, 56.3; LRMS (ESI-) *m*/*z* 250 (M − H)^−^.

##### (Z)-5-(2,4-Dimethoxybenzylidene)-4-thioxothiazolidin-2-one (Compound **2k**)

Reaction time, 15 min; yield, 64%; ^1^H NMR (500 MHz, DMSO-*d*_6_) *δ* 13.66 (brs, 1H, NH), 8.37 (s, 1H, vinylic H), 7.43 (d, 1H, *J* = 8.5 Hz, 6-H), 6.70* (d, 1H, *J* = 8.5 Hz, 5-H), 6.68* (s, 1H, 3-H), 3.89 (s, 3H, OCH_3_), 3.85 (s, 3H, OCH_3_); ^13^C NMR (100 MHz, DMSO-*d*_6_) *δ* 197.0, 172.2, 164.3, 161.5, 131.2, 130.7, 128.1, 116.0, 107.5, 99.3, 56.8, 56.4; LRMS (ESI-) *m*/*z* 280 (M − H)^−^.

##### (Z)-5-(3,4-Dimethoxybenzylidene)-4-thioxothiazolidin-2-one (Compound **2l**)

Reaction time, 20 min; yield, 35%; ^1^H NMR (500 MHz, DMSO-*d*_6_) *δ* 13.72 (brs, 1H, NH), 8.06 (s, 1H, vinylic H), 7.27 (dd, 1H, *J* = 8.5, 2.0 Hz, 6-H), 7.24 (d, 1H, *J* = 2.0 Hz, 2-H), 7.13 (d, 1H, *J* = 8.5 Hz, 5-H), 3.83 (s, 3H, OCH_3_), 3.81 (s, 3H, OCH_3_); ^13^C NMR (100 MHz, DMSO-*d*_6_) *δ* 196.0, 171.5, 152.1, 149.7, 137.3, 128.0, 126.9, 125.3, 114.3, 112.9, 56.5, 56.3; LRMS (ESI-) *m*/*z* 280 (M − H)^−^.

### 4.2. Tyrosinase Inhibition—Kinetic and In Silico Studies

#### 4.2.1. Mushroom Tyrosinase Inhibition Assay

The tyrosinase inhibitory activity assay for compounds **2a**–**2l** was performed against mushroom tyrosinase, according to a previously described method [43], with minor modifications. In brief, a 200-microliter mixture consisting of a solution (10 µL) of compounds **2a**–**2l** at various concentrations, a tyrosinase solution (20 µL, 150 units), and a substrate solution (170 µL), comprising 17.2 mM potassium phosphate buffer and 345 µM L-tyrosine solution, was added to 96-well microplates and incubated at 37 °C in a humidified 5% CO_2_ atmosphere for 30 min. The absorbances of dopachrome produced in each well during incubation was measured at 450 nm using a microplate reader (VersaMax™, Molecular Devices, Sunnyvale, CA, USA). Kojic acid, a well-known tyrosinase inhibitor, was used as a positive control. All experiments were independently conducted 3 times. To calculate the % inhibition of tyrosinase activity, the following formula was used: % inhibition = [1 − (A/B) × 100], where A means the optical density of the test samples and B means the optical density of the non-treated control. To calculate the concentration necessary for 50% inhibition of enzyme activity (IC_50_), the % tyrosinase inhibition of each compound was obtained at 3 or more different concentrations. IC_50_ values were calculated by constructing a linear regression curve showing the concentration of the (*Z*)-BTTZ compounds on the x-axis and percentage inhibition on the y-axis. To obtain a negative control dimethyl sulfoxide (DMSO) was added instead of the (*Z*)-BTTZ compounds.

#### 4.2.2. Kinetic Studies on Mushroom Tyrosinase Inhibition by **2b** and **2f**

Compounds **2b** or **2f** (10 µL, final concentrations: 0, 0.05, 0.1, or 0.2 µM for **2b** and 0, 2, 4, or 8 µM for **2f**) and mushroom tyrosinase solution (20 µL, 150 units) were added to the wells of a 96-well plate containing aqueous solutions (170 µL) containing L-tyrosine at 0.5, 1.0, 2.0, 4.0, or 8.0 mM (final concentrations), distilled water, and potassium phosphate buffer (final concentration: 14.7 mM, pH 6.5), in the ratio 10:9:10. Initial rates of dopachrome formation in reaction mixtures were calculated by measuring increases in absorbances at 492 nm (ΔOD_492_/min) using a microplate reader (VersaMax^TM^, Molecular Devices, Sunnyvale, CA, USA). Maximal velocity (V_max_) and Michaelis constant (K_M_) were determined using Lineweaver–Burk plots obtained by plotting the inverse of reaction velocity (1/V) against the inverse of substrate concentration (1/[S]) obtained at five L-tyrosine concentrations. Modes of tyrosinase inhibition were determined using points of convergence.

#### 4.2.3. In Silico Study of Interactions between Mushroom Tyrosinase and Compounds **2b** and **2f** and Kojic Acid

The in silico study was conducted using Schrödinger Suite (2021-1) with minor modifications, as previously described [44]. The crystal structure of *m*TYR (*Agaricus bisporus*, PDB: 2Y9X) was downloaded from the Protein Data Bank (PDB) to Maestro12.4’s Protein Preparation Wizard, and processed, and unwanted protein chains were removed. To optimize the structure, hydrogen atoms were added, water molecules > 3 Å away from the enzyme were removed, and the structure was minimized. The glide grid and the active site were determined using the binding site of tropolone (a ligand of tyrosinase) obtained from PDB and the literature [45,46,47]. The structures of compounds **2b** and **2f** and kojic acid were imported into the entry list of Maestro in CDXML format. Before ligand docking, the structures of **2b** and **2f** and kojic acid were prepared using LigPrep. The compounds were then docked to the enzyme’s glide grid using Glide from the Maestro task list [48]. Binding affinities and ligand–protein interactions were obtained using the glide extra precision (XP) method [49].

#### 4.2.4. In Silico Study of Interactions between Compounds **2b** and **2f** and Kojic Acid and the Human Tyrosinase Homology Model

The *h*TYR homology model was created using the Swiss-Model online server (https://swissmodel.expasy.org; accessed on 4 April 2021) and Schrödinger Suite (2020–2) using default settings. The protein sequence of *h*TYR (P14679) was imported from the UniProt database (https://www.uniprot.org; accessed on 4 April 2021) and the homology model was generated in the Swiss-Model online server using the TRP1 (PDB: 5M8Q) template. The model was further processed using Schrödinger Suite and validated using Schrödinger prime (a homology modeling tool in Schrödinger Suite). Compounds **2b** and **2f** and kojic acid were docked with the processed human homology model using the same protocols mentioned above for *m*TYR docking.

### 4.3. Cell Culture

Murine melanoma B16F10 cells were obtained from the American Type Culture Collection (ATCC, Manassas, VA, USA). Fetal bovine serum (FBS), Dulbecco’s modified Eagle’s medium (DMEM), phosphate buffer solution (PBS), penicillin, streptomycin, and trypsin were purchased from Gibco/Thermo Fisher Scientific (Carlsbad, CA, USA). B16F10 cells were cultured in DMEM containing penicillin/streptomycin (100 IU/100 µg/mL) and 10% heat-inactivated FBS in a standard humidified 5% CO_2_ atmosphere at 37 °C. Cell viability, anti-tyrosinase activity, and anti-melanogenic activity experiments were conducted on these cells cultured in 24- or 96-well plates.

### 4.4. Cell Viability Assays

Cell viability assays of compounds **2b** and **2f** were performed using B16F10 melanoma cells and the EZ-Cytox assay (EZ-3000, DoGenBio, Seoul, Korea), as previously described [50]. Briefly, cells were seeded at a density of 1 × 10^4^ cells/well in a 96-well plate for 24 h, and then were exposed to different concentrations (0, 1, 2.5, 5, 10, or 25 µM) of **2b** or **2f** and incubated at 37 °C for 24 and 48 h, respectively. EZ-Cytox solution (10 µL) was then added to each well and cells were incubated at 37 °C for 2 h. Absorbances were measured at 450 nm using a microplate reader (VersaMax^TM^, Molecular Devices, Sunnyvale, CA, USA). Each assay was performed in triplicate.

### 4.5. Anti-Melanogenesis Activity Assays

The anti-melanogenesis activities of compounds **2b** and **2f** were assessed using a standard melanin content assay with minor modifications [51]. Briefly, B16F10 cells were seeded at a density of 5 × 10^4^/well in a 24-well plate and allowed to adhere to wells in a DMEM solution containing penicillin/streptomycin (100 IU/100 µg/mL) and 10% heat-inactivated FBS in a standard humidified 5% CO_2_ atmosphere at 37 °C. After culture for 24 h, cells were exposed to α-MSH (0.5 µM for **2b** and 1.0 µM for **2f**) and various concentrations (0, 5, 10, or 25 µM) of **2b** or **2f** or kojic acid at 25 µM in a standard humidified 5% CO_2_ atmosphere at 37 °C for 48 h. Cells were then rinsed twice with PBS and lysed in 200 µL of 1N NaOH containing 10% dimethyl sulfoxide (DMSO) for 1 h at 60 °C. Lysates were transferred to the wells of a 96-well plate, and melanin absorbances were measured at 405 nm using a microplate reader (VersaMax^TM^, Molecular Devices, Sunnyvale, CA, USA). All experiments were conducted three times in triplicate. Kojic acid, 1 N NaOH, DMSO, and α-MSH were purchased from Sigma-Aldrich (St. Louis, MO, USA).

### 4.6. Anti-Tyrosinase Activity Assays

3,4-Dihydroxy-L-phenylalanine (L-DOPA), phenylmethylsulfonyl fluoride (PMSF), 4-(1,1,3,3-tetramethylbutyl)phenylpolyethylene glycol (Triton^TM^ X-100), potassium hydrogen phosphate, and potassium dihydrogen phosphate were purchased from Sigma-Aldrich (St. Louis, MO, USA).

Tyrosinase activities were assessed by determining the L-DOPA oxidation rate, as described previously with minor modifications [52]. Briefly, B16F10 cells were seeded at 5 × 10^4^ cells/well in a 24-well plate, allowed to adhere to wells in a DMEM solution containing penicillin/streptomycin (100 IU/100 µg/mL) and 10% heat-inactivated FBS in a standard humidified 5% CO_2_ atmosphere at 37 °C for 24 h, exposed to α-MSH (0.5 µM for **2b** and 1.0 µM for **2f**) and various concentrations (0, 5, 10, or 25 µM) of **2b** or **2f** or kojic acid (25 µM), and incubated in a standard humidified 5% CO_2_ atmosphere at 37 °C for 48 h. Cells were then rinsed twice with PBS, exposed to 100 µL of lysis buffer (90 µL of 50 mM phosphate buffer pH 6.8; 5 µL of 2 mM PMSF; 5 µL of 20% Triton), and frozen at −80 °C for 30 min. After defrosting, lysates were centrifuged at 12,000 *g* for 30 min at 4 °C, and supernatants (80 µL) were mixed with 10 mM L-DOPA (20 µL) in a 96-well plate. Absorbances were measured at 490 nm at 37 °C every 10 min for 1 h using a microplate reader (VersaMax^TM^, Molecular Devices, Sunnyvale, CA, USA). All experiments were carried out three times in triplicate.

### 4.7. Statistical Analysis

One-way analysis of variance (ANOVA) followed by Bonferroni post hoc test was used to determine the significances of differences between treatments. The statistical analysis was carried out using GraphPad Prism 5 software (La Jolla, CA, USA). Results are presented as means ± standard errors of means (SEMs). Two-sided *p*-values of <0.05 were considered significant.

## Figures and Tables

**Table 1:** Substitution patterns and IC_50_ values of compounds **2a**–**2l** and kojic acid against mushroom tyrosinase and their log *p* values.

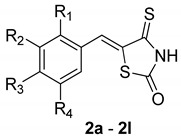						
Compound	R_1_	R_2_	R_3_	R_4_	IC_50_ (µM)	^a^ Log *p*
**2a**	H	H	OH	H	28.05 ± 1.16	1.65
**2b**	OH	H	OH	H	0.47 ± 0.97	1.26
**2c**	H	OH	OH	H	47.41 ± 2.18	1.26
**2d**	H	OMe	OH	H	70.75 ± 1.00	1.52
**2e**	H	OEt	OH	H	92.81 ± 0.89	1.86
**2f**	H	OH	OMe	H	15.24 ± 1.68	1.52
**2g**	H	OMe	OH	OMe	82.39 ± 1.26	1.39
**2h**	OH	H	H	H	147.61 ± 0.96	1.65
**2i**	H	Br	OH	H	26.27 ± 4.10	2.47
**2j**	H	H	OMe	H	30.83 ± 1.41	1.91
**2k**	OMe	H	OMe	H	126.35 ± 0.46	1.78
**2l**	H	OMe	OMe	H	23.31 ± 0.28	1.78
Kojic acid					66.30 ± 0.75	−2.45

^a^ Log *p* values were obtained using ChemDraw Ultra 12.0.

## Data Availability

Data are available from the authors upon reasonable request.

## Supplementary Materials

The following are available online. ^1^H- and ^13^C NMR and ESI-mass data of compounds **1a** and **2a**–**2l** are provided in the Supplementary Materials.

## Sample Availability

Samples of the compounds are available from the authors.
