# Supplementary material for: Novel Anti-Melanogenic Compounds, (Z)-5-(Substituted Benzylidene)-4-thioxothiazolidin-2-one Derivatives: In Vitro and In Silico Insights
Source: Molecules. 2021 Aug 17;26(16):4963. doi: 10.3390/molecules26164963 (PMC8400311; doi:10.3390/molecules26164963)
Supplement: Supplementary file 1 [file molecules-26-04963-s001.zip › molecules-1329992-supplementary.pdf]

Supplementary material

For

Novel anti-melanogenic compounds, (*Z*)-5-(substituted benzylidene)-4-thioxothiazolidin-2-one derivatives: *In vitro* and *in silico* insights

Heejeong Choi[1,†], Il Young Ryu[1,†], Inkyu Choi[1,†], Sultan Ullah[2,†], Hee Jin Jung[1], Yujin Park[1], Yeongmu Jeong[1], YeJi Hwang[1], Sojeong Hong[1], In-Soo Yoon[1], Hwayoung Yun[1], Min-Soo Kim[1], Jin-Wook Yoo[1], Yunjin Jung[1], Pusoon Chun[3,4,*] and Hyung Ryong Moon[1,*]

[1]*Laboratory of Medicinal Chemistry, College of Pharmacy, Pusan National University, Busan 46241, South Korea*

[2]*Department of Molecular Medicine, The Scripps Research Institute, Florida 33458, USA*

[3]*College of Pharmacy, Inje University, Gimhae, Gyeongnam 50834, South Korea*

[4]*Inje Institute of Pharmaceutical Sciences and Research, Inje University, Gimhae, Gyeongnam 50834, South Korea*

Contents

S1. ^{1}H NMR spectrum of compound **2a**……………………………………………………………4

S2. ^{13}C NMR spectrum of compound **2a**………………………………………………………...5

S3. LRMS spectrum of compound **2a**…………………………..……………….……6

S4. ^{1}H NMR spectrum of compound **2b**……………………………………………………………7

S5. ^{13}C NMR spectrum of compound **2b**………………………………………………………...8

S6. ^{1}H-coupled ^{13}C NMR spectrum of compound **2b**…………………….………………...9

S7. LRMS spectrum of compound **2b**…………………………..…..……………..…10

S8. ^{1}H NMR spectrum of compound **2c**………………………………………………………...11

S9. ^{13}C NMR spectrum of compound **2c**……………………………………………………..12

S10. LRMS spectrum of compound **2c**…………………………...……………..……13

S11. ^{1}H NMR spectrum of compound **2d**…………………………………………………14

S12. ^{13}C NMR spectrum of compound **2d**……………………………..………………15

S13. LRMS spectrum of compound **2d**…………………………………..……..……16

S14. ^{1}H NMR spectrum of compound **2e**……………………………………..……...17

S15. ^{13}C NMR spectrum of compound **2e**……………………………………………………18

S16. LRMS spectrum of compound **2e**…………………………………..……..…19

S17. ^{1}H NMR spectrum of compound **2f**………………………………………………..…20

S18. ^{13}C NMR spectrum of compound **2f**…………………………………………………21

S19. LRMS spectrum of compound **2f**…………………………………………..……22

S20. ^{1}H NMR spectrum of compound **2g**…………………………………………………23

S21. ^{13}C NMR spectrum of compound **2g**……………………………...…………24

S22. LRMS spectrum of compound **2g**…………………………………..……..……..…25

S23. ^{1}H NMR spectrum of compound **2h**……………………………………………………26

S24. ^{13}C NMR spectrum of compound **2h**..27

S25. LRMS spectrum of compound **2h**..28

S26. ^{1}H NMR spectrum of compound **2i**..29

S27. ^{13}C NMR spectrum of compound **2i**..30

S28. LRMS spectrum of compound **2i**..31

S29. ^{1}H NMR spectrum of compound **2j**..32

S30. ^{13}C NMR spectrum of compound **2j**..33

S31. LRMS spectrum of compound **2j**..34

S32. ^{1}H NMR spectrum of compound **2k**..35

S33. ^{13}C NMR spectrum of compound **2k**..36

S34. LRMS spectrum of compound **2k**..37

S35. ^{1}H NMR spectrum of compound **2l**..38

S36. ^{13}C NMR spectrum of compound **2l**..39

S37. LRMS spectrum of compound **2l**..40

S38. ^{1}H NMR spectrum of compound **1a**..41

S39. ^{13}C NMR spectrum of compound **1a**..42

S40. LRMS spectrum of compound **1a**..43

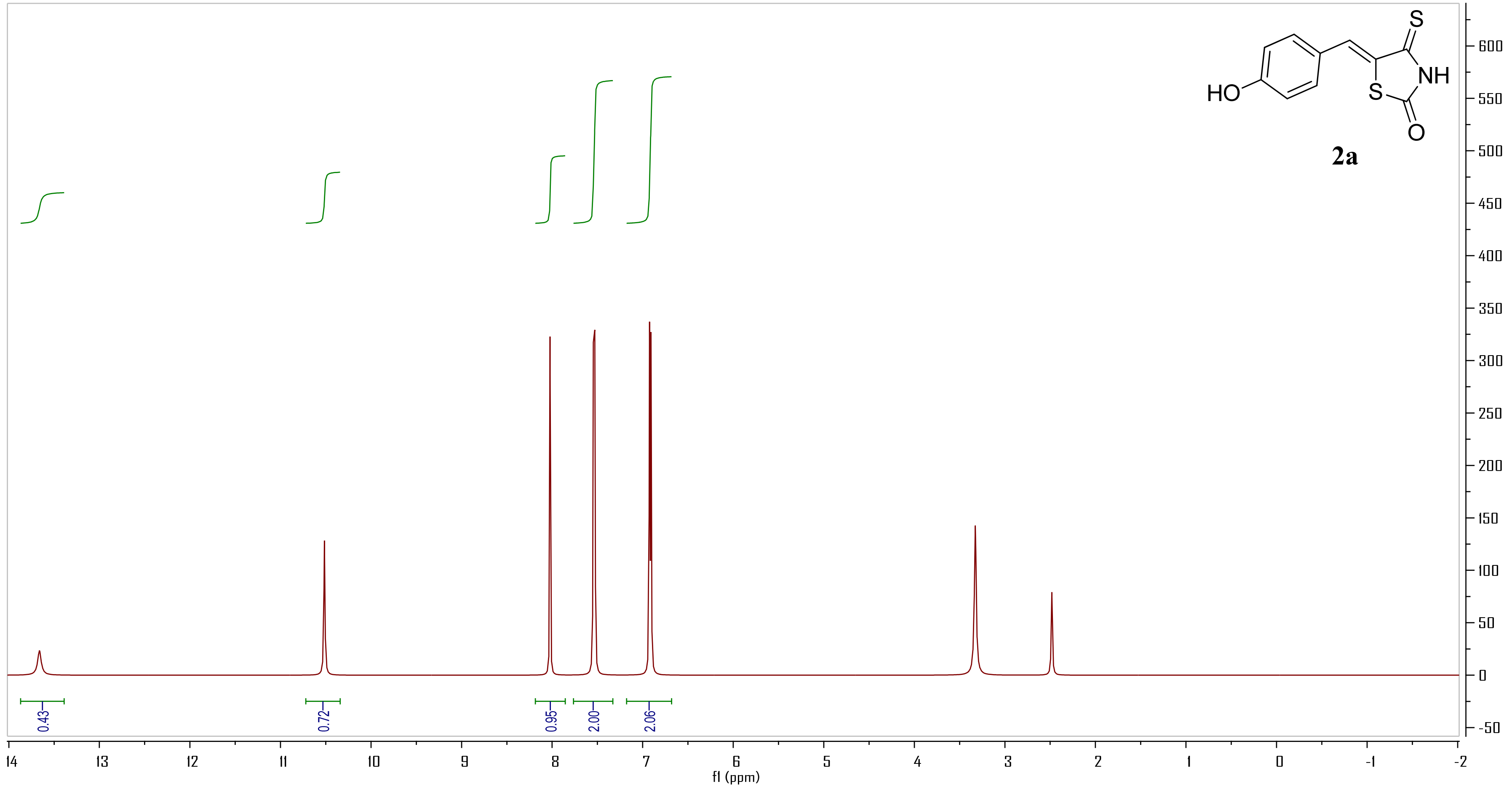

S1. ^{1}H NMR spectrum of compound **2a**

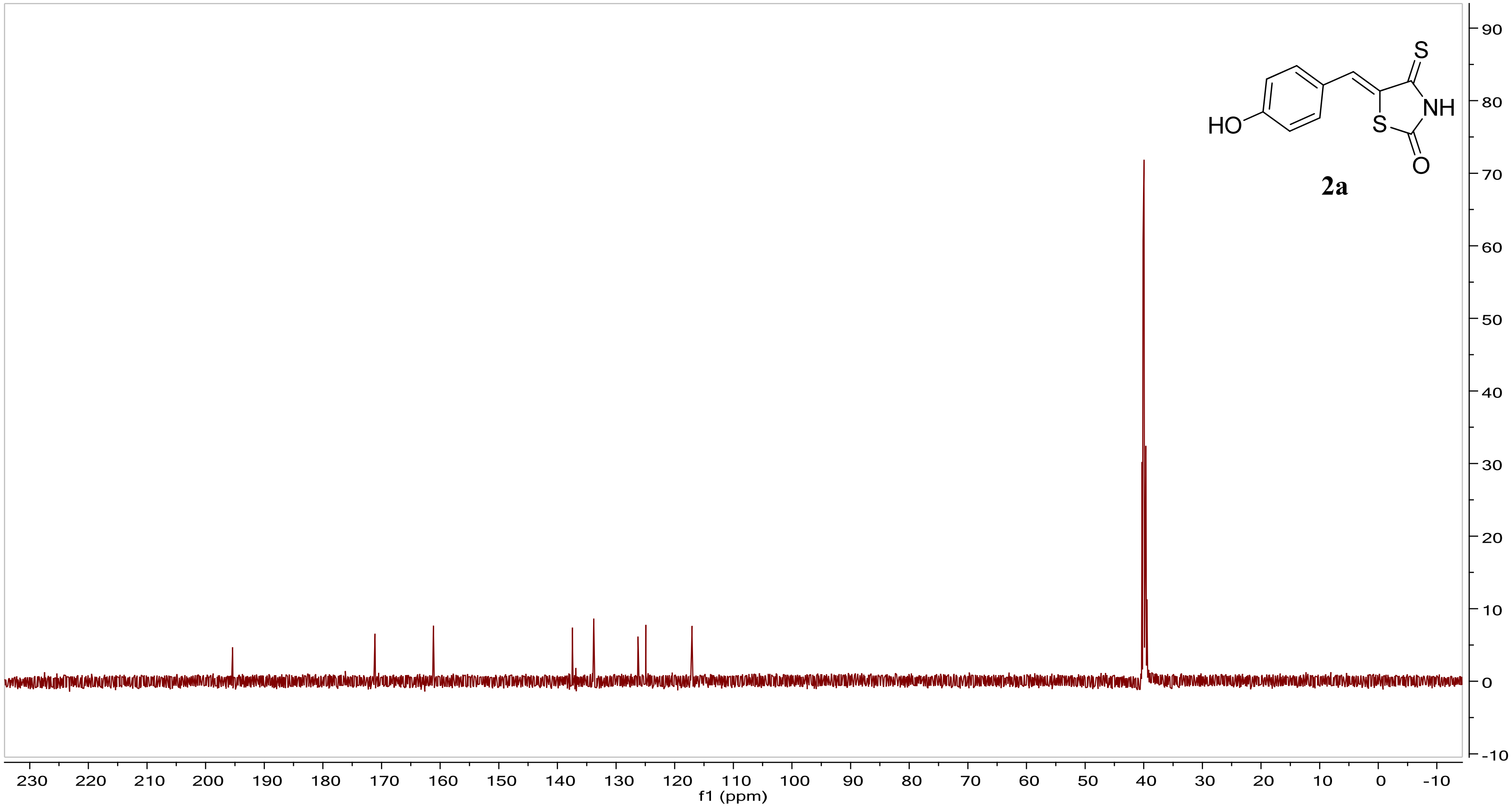

S2. ^{13}C NMR spectrum of compound **2a**

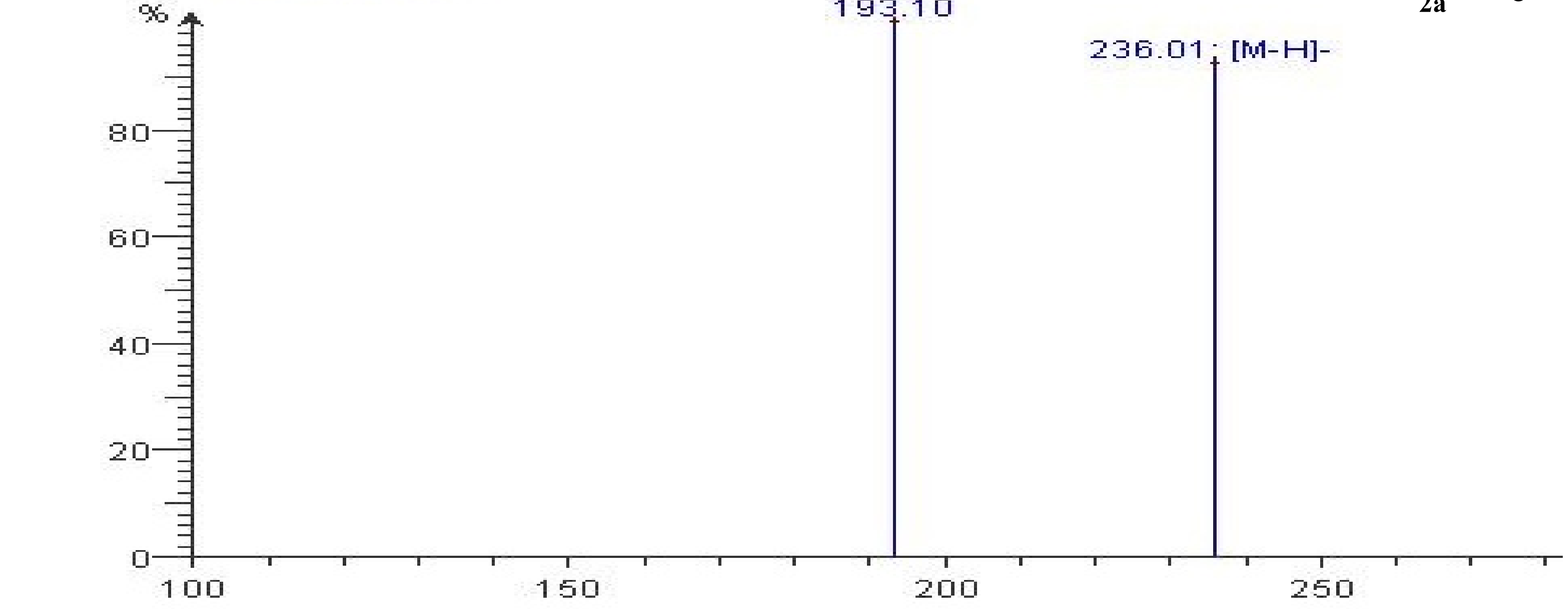

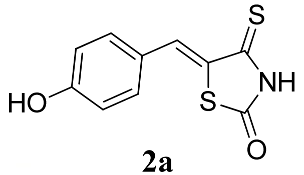

2a

S3. LRMS spectrum of compound **2a**

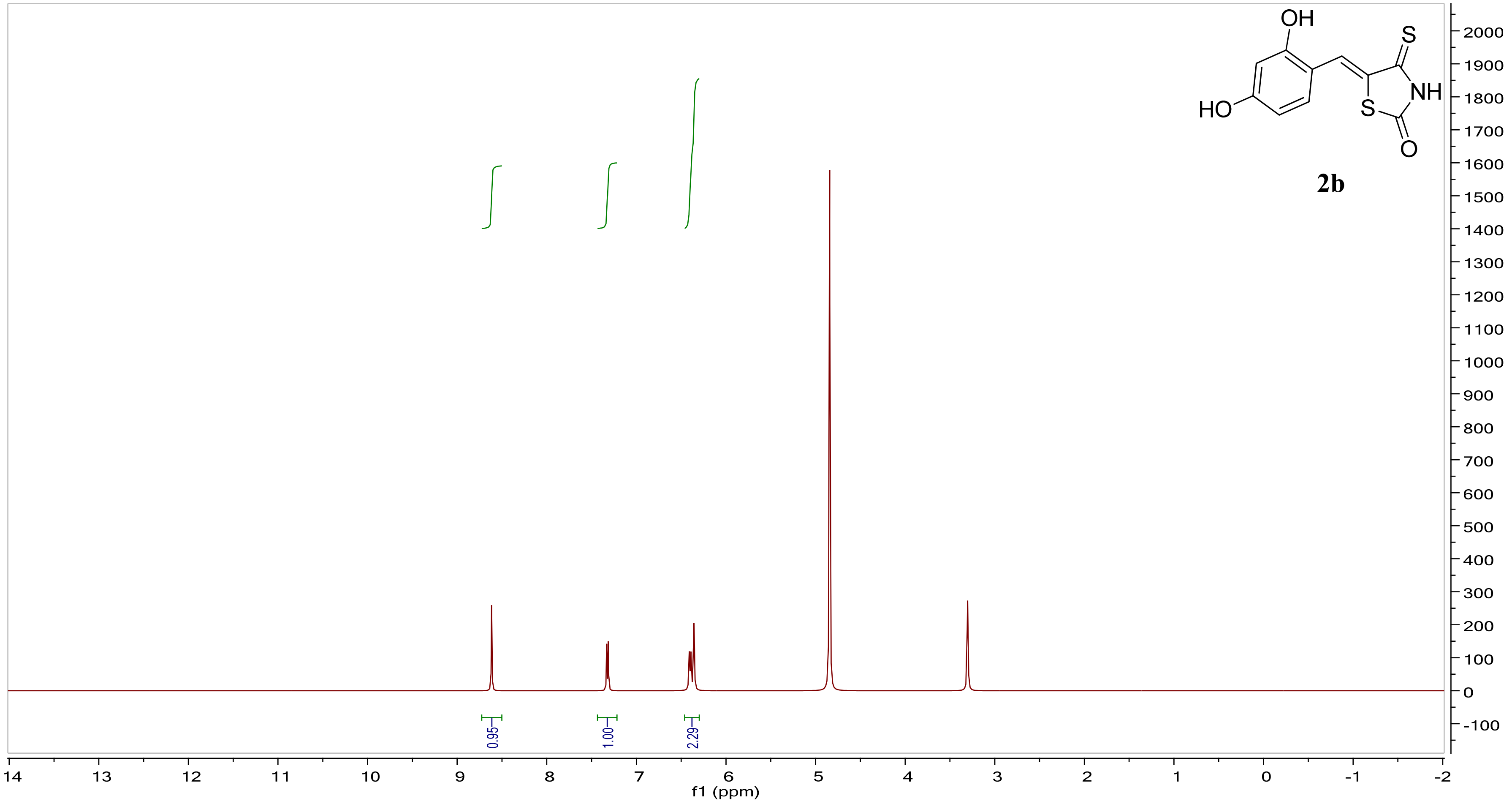

S4. ^{1}H NMR spectrum of compound **2b**

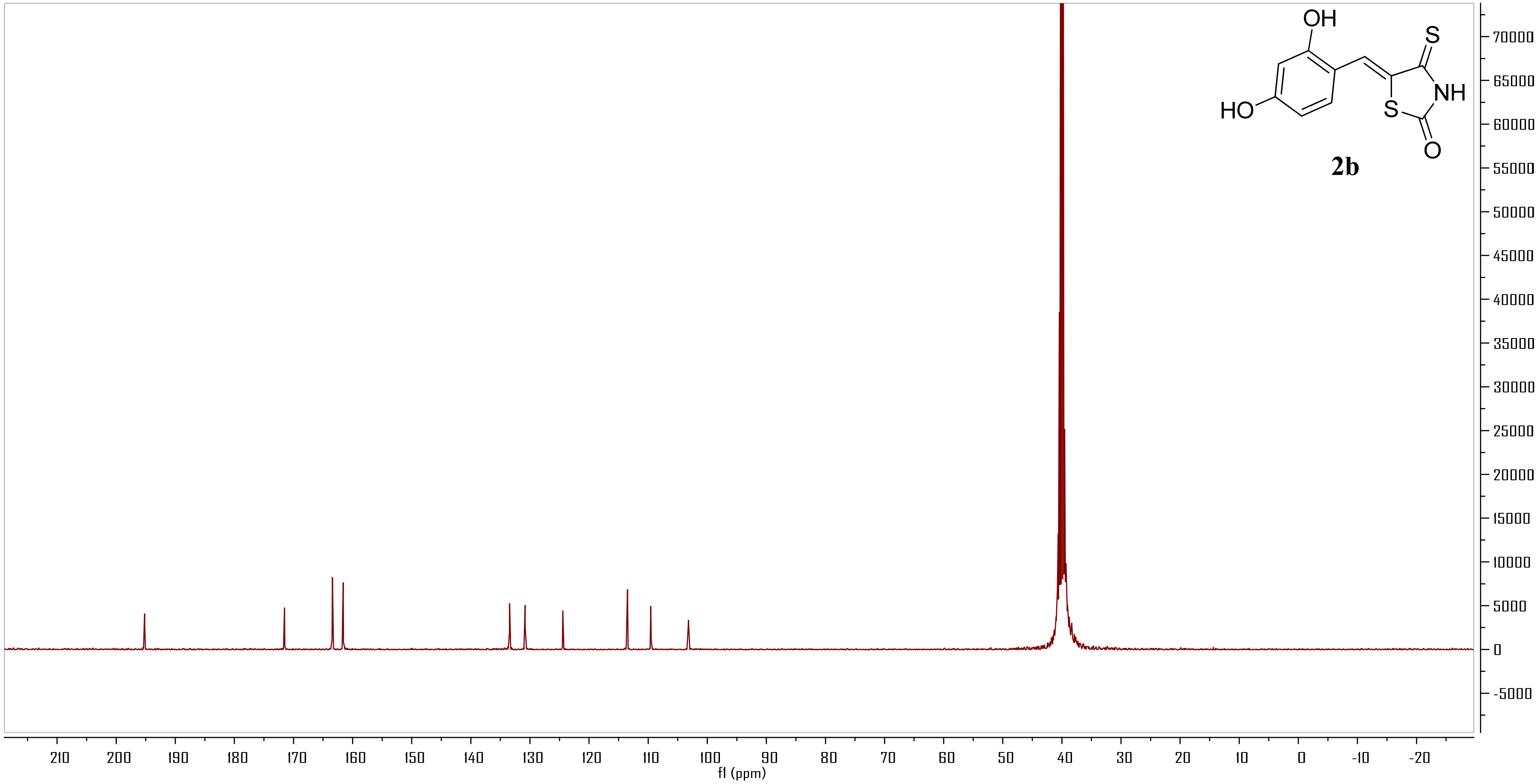

S5. ^{13}C NMR spectrum of compound **2b**

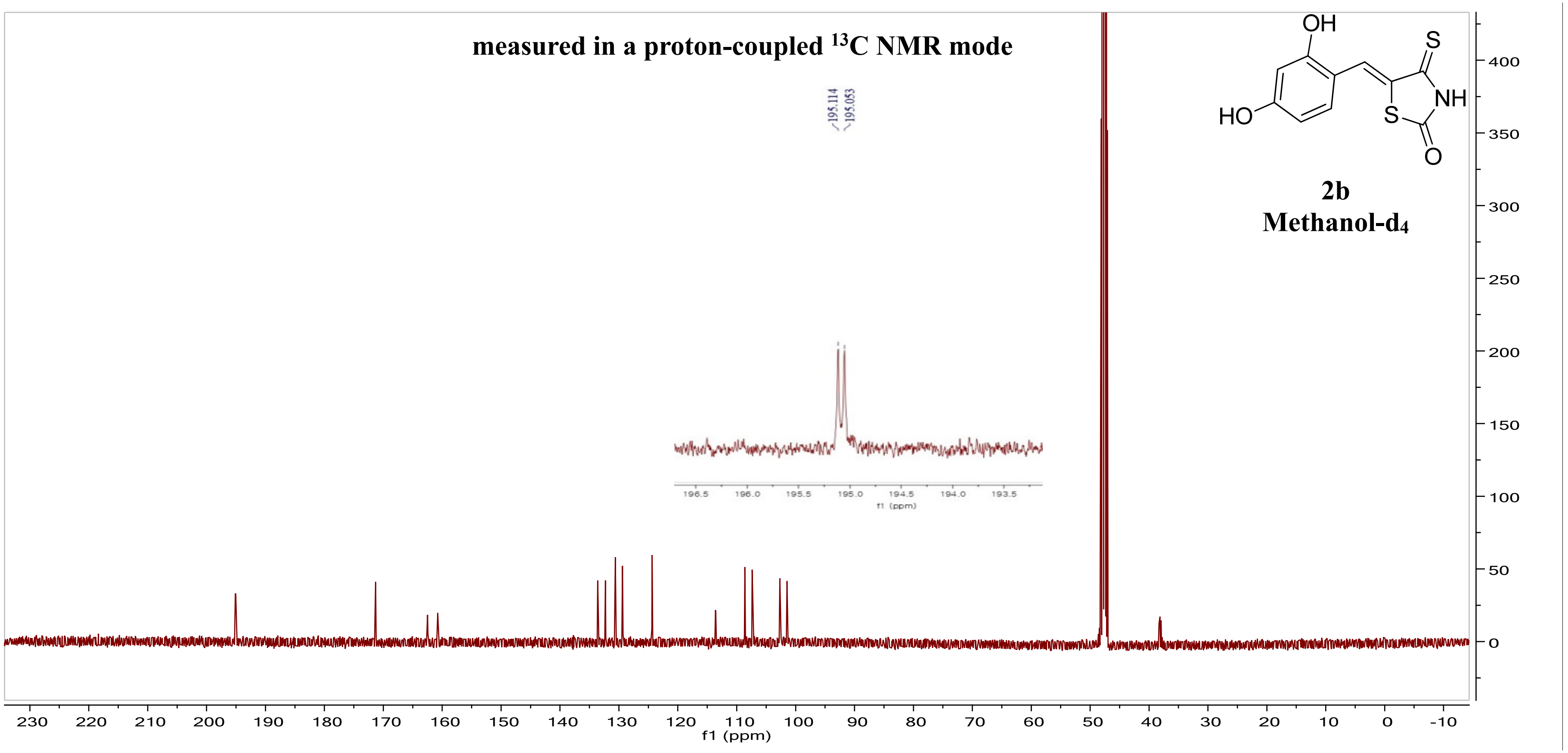

S6. ^{1}H-coupled ^{13}C NMR spectrum of compound **2b**

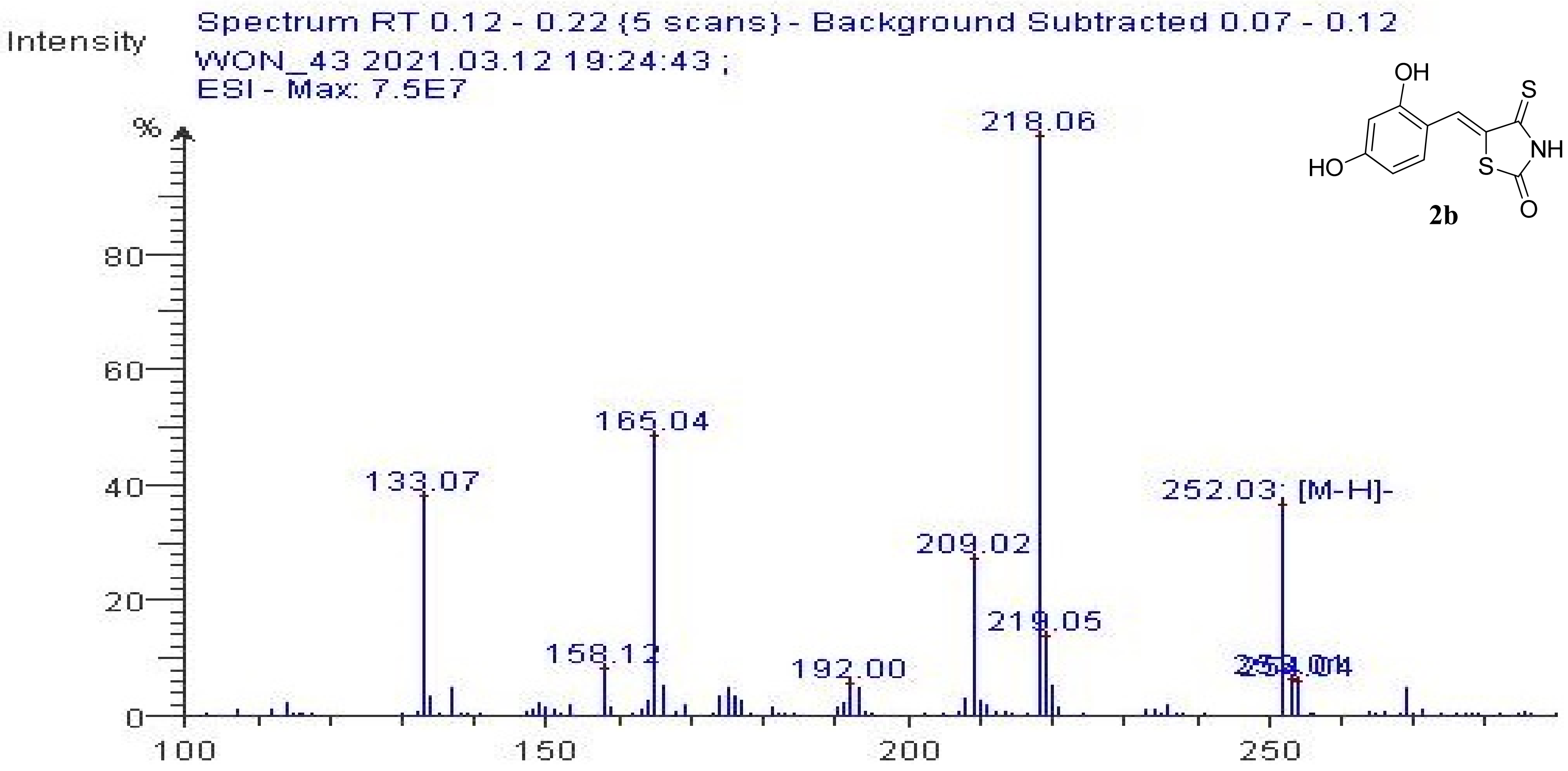

S7. LRMS spectrum of compound **2b**

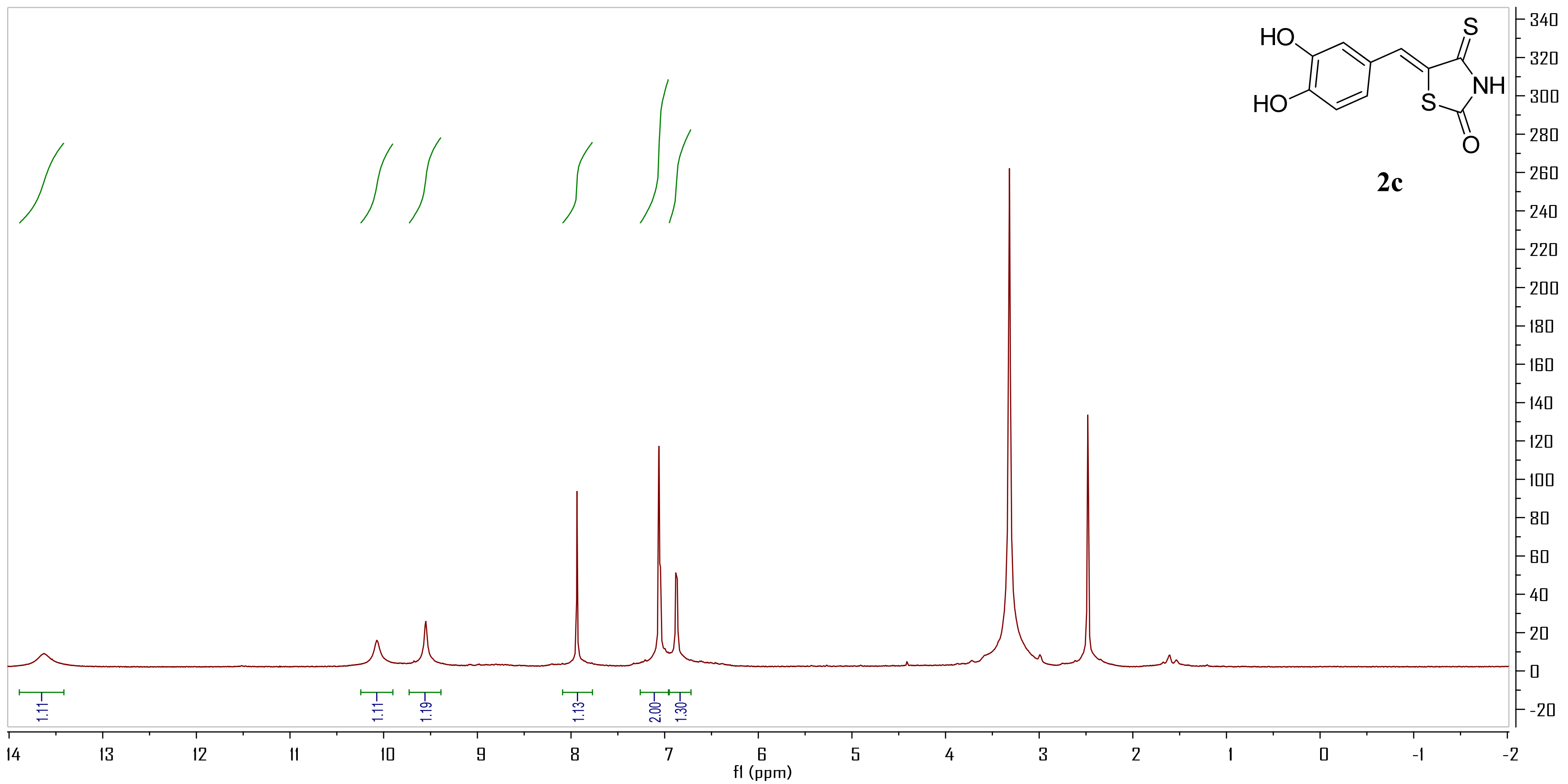

S8. ^{1}H NMR spectrum of compound **2c**

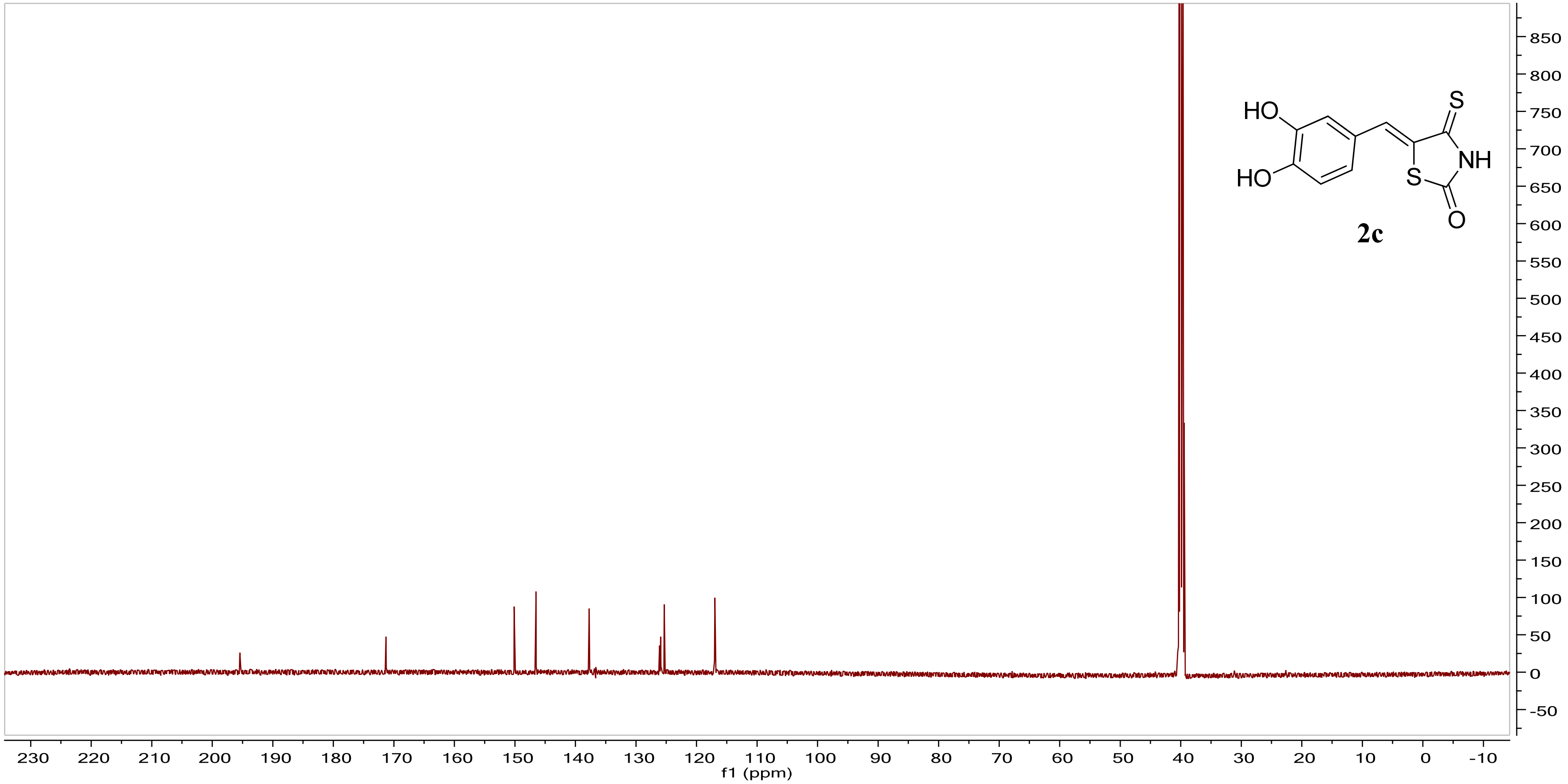

S9. ^{13}C NMR spectrum of compound **2c**

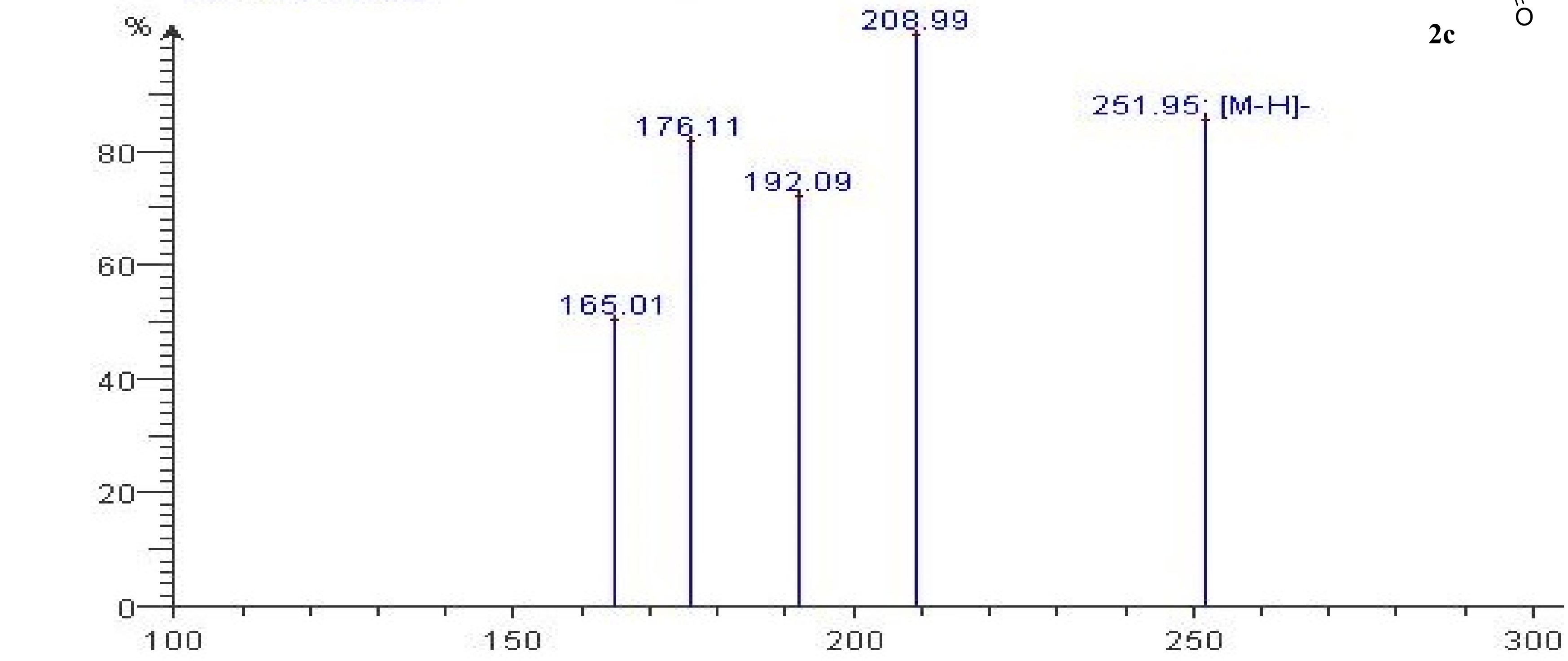

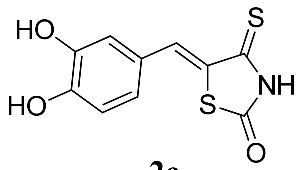

2c

S10. LRMS spectrum of compound **2c**

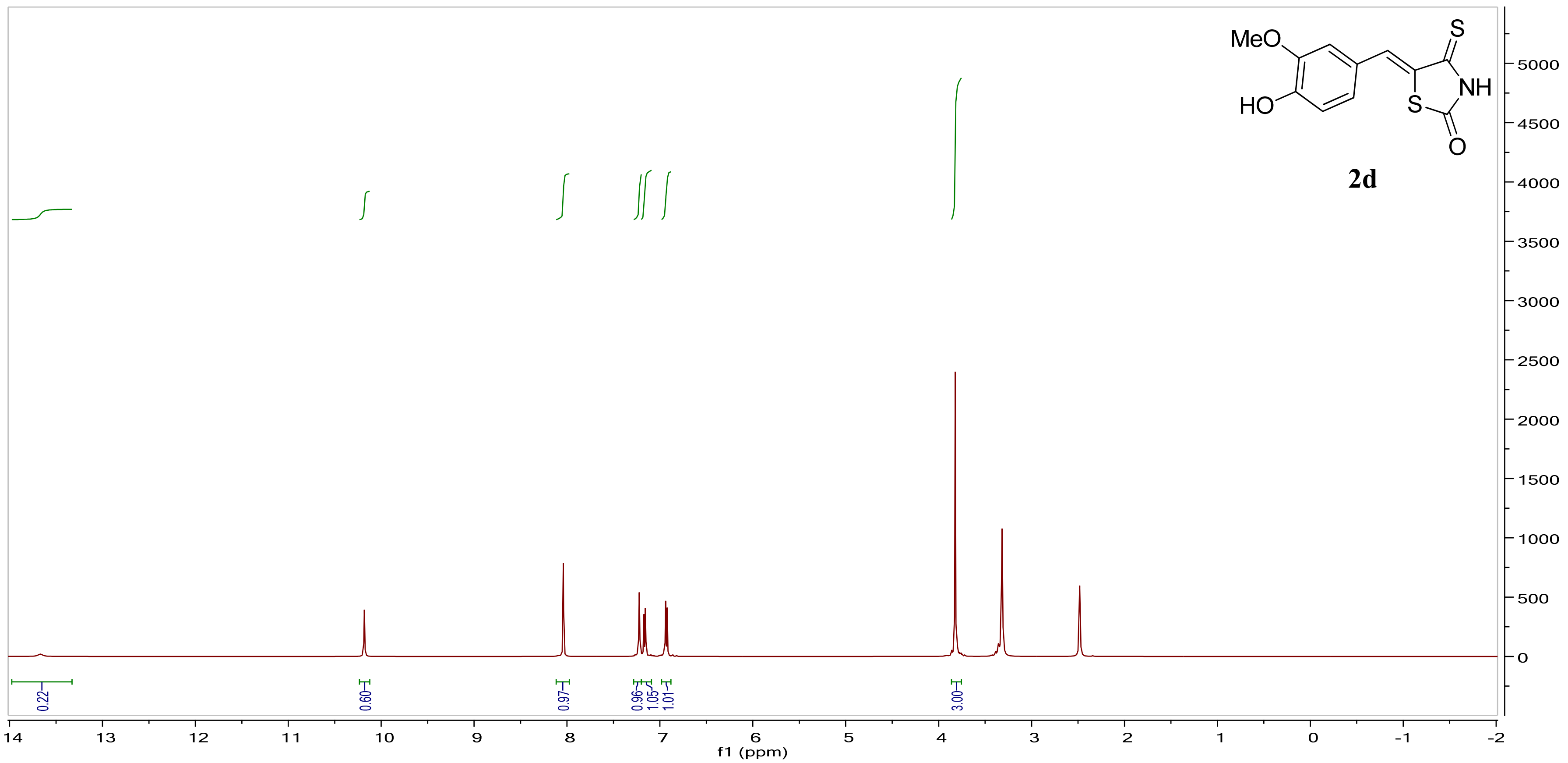

S11. ^{1}H NMR spectrum of compound **2d**

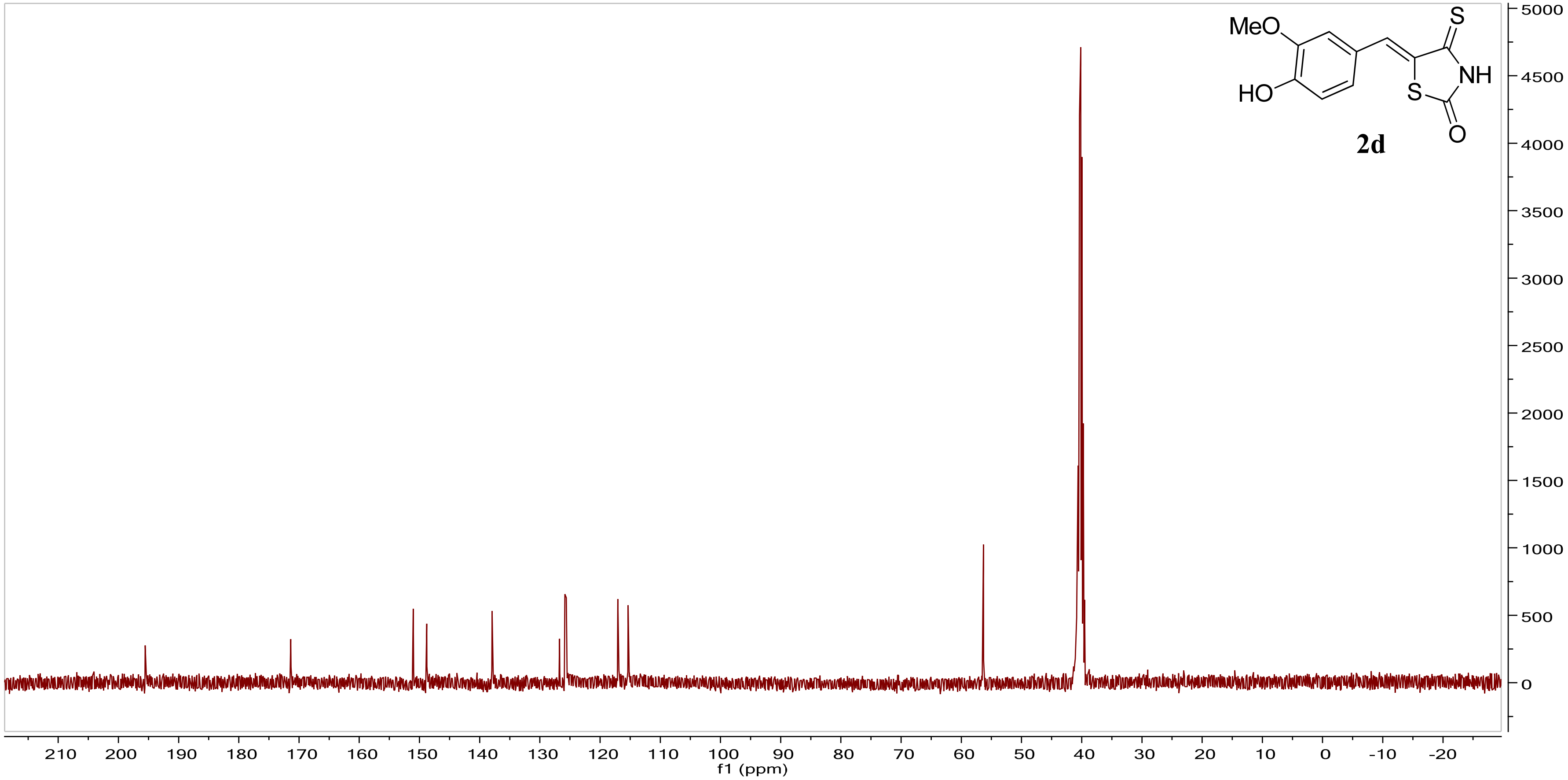

S12. ^{13}C NMR spectrum of compound **2d**

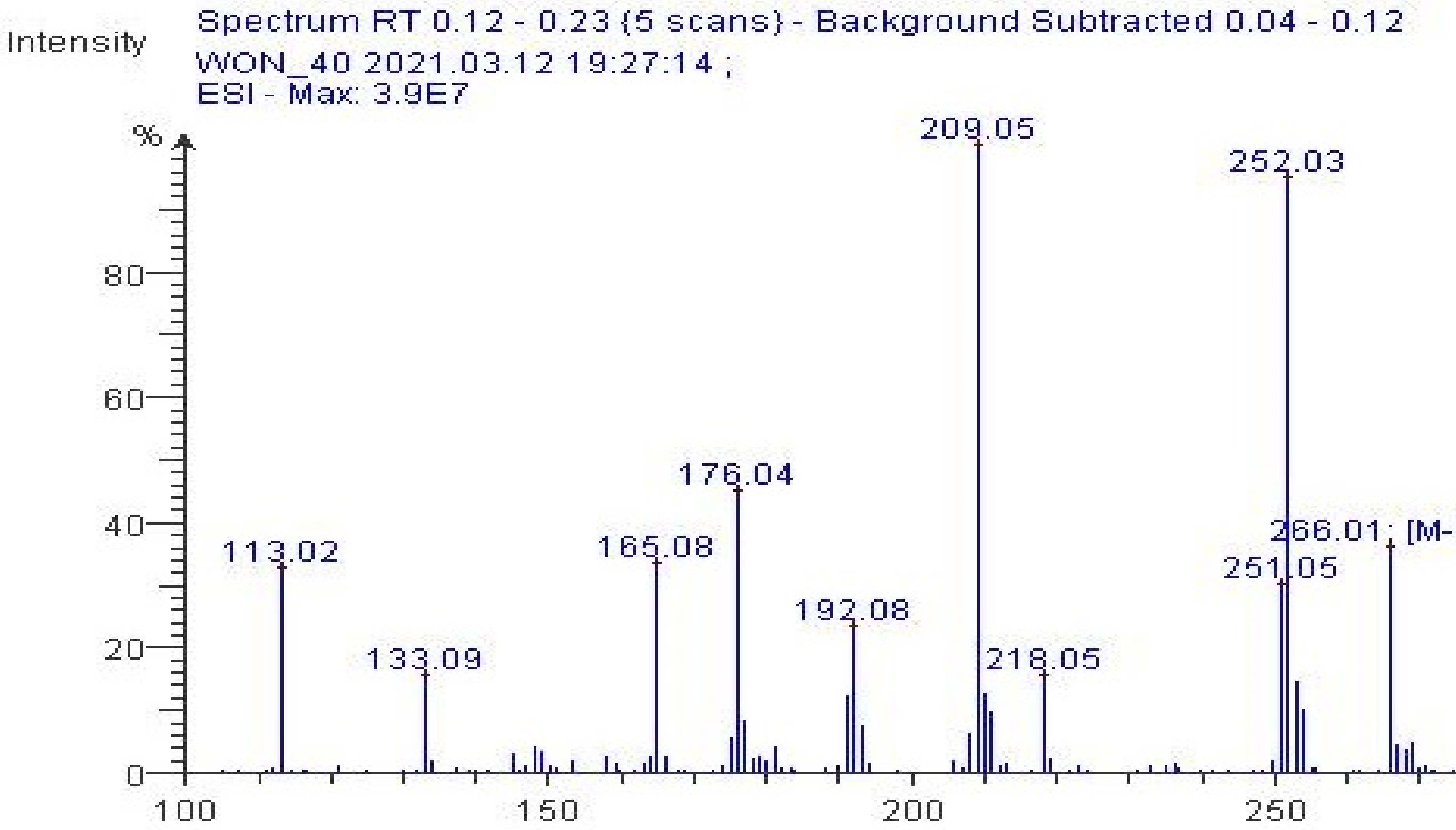

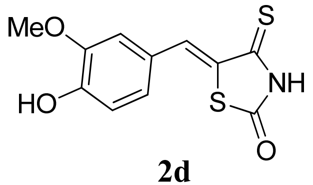

2d

S13. LRMS spectrum of compound **2d**

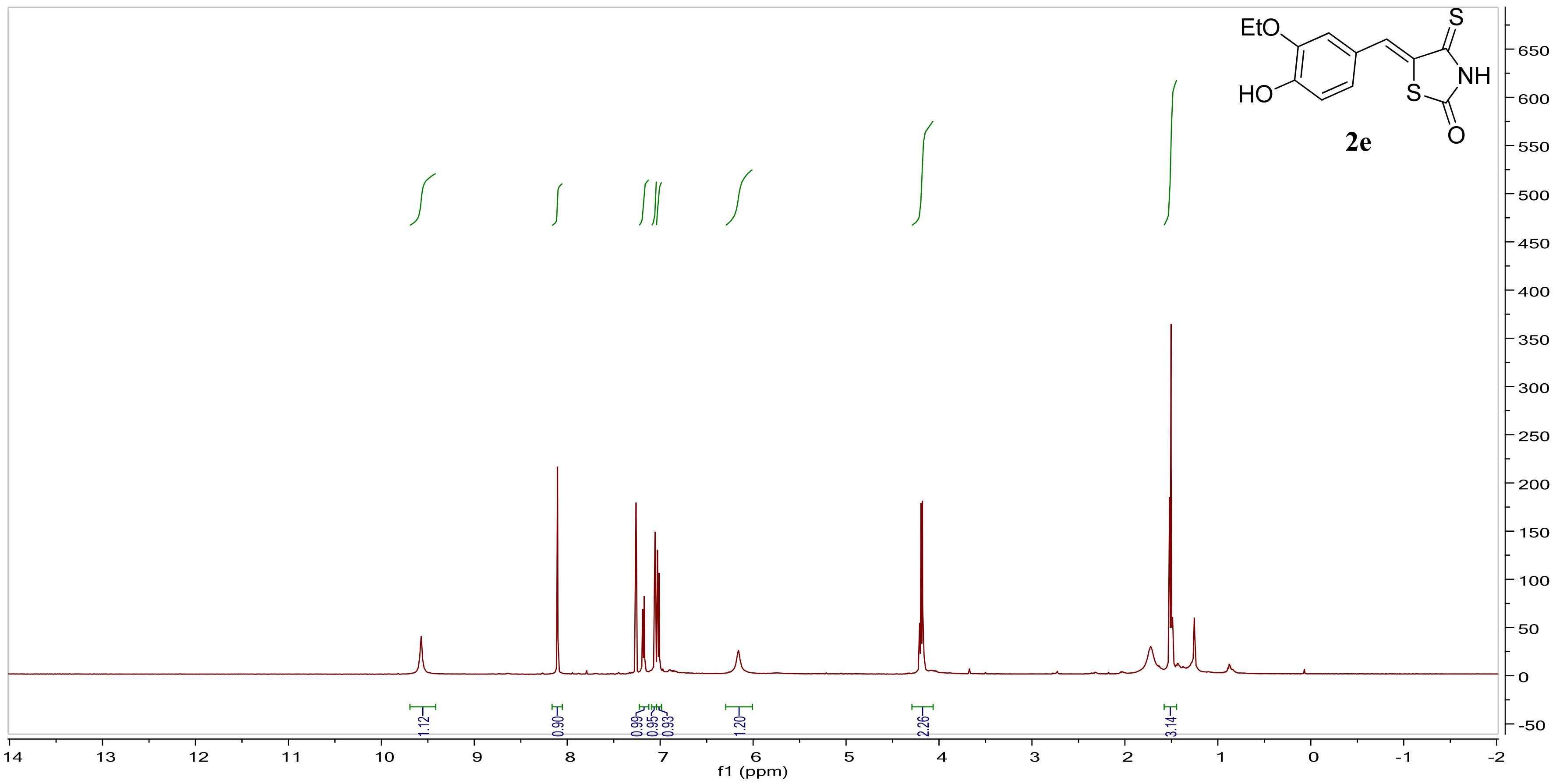

S14. ^{1}H NMR spectrum of compound **2e**

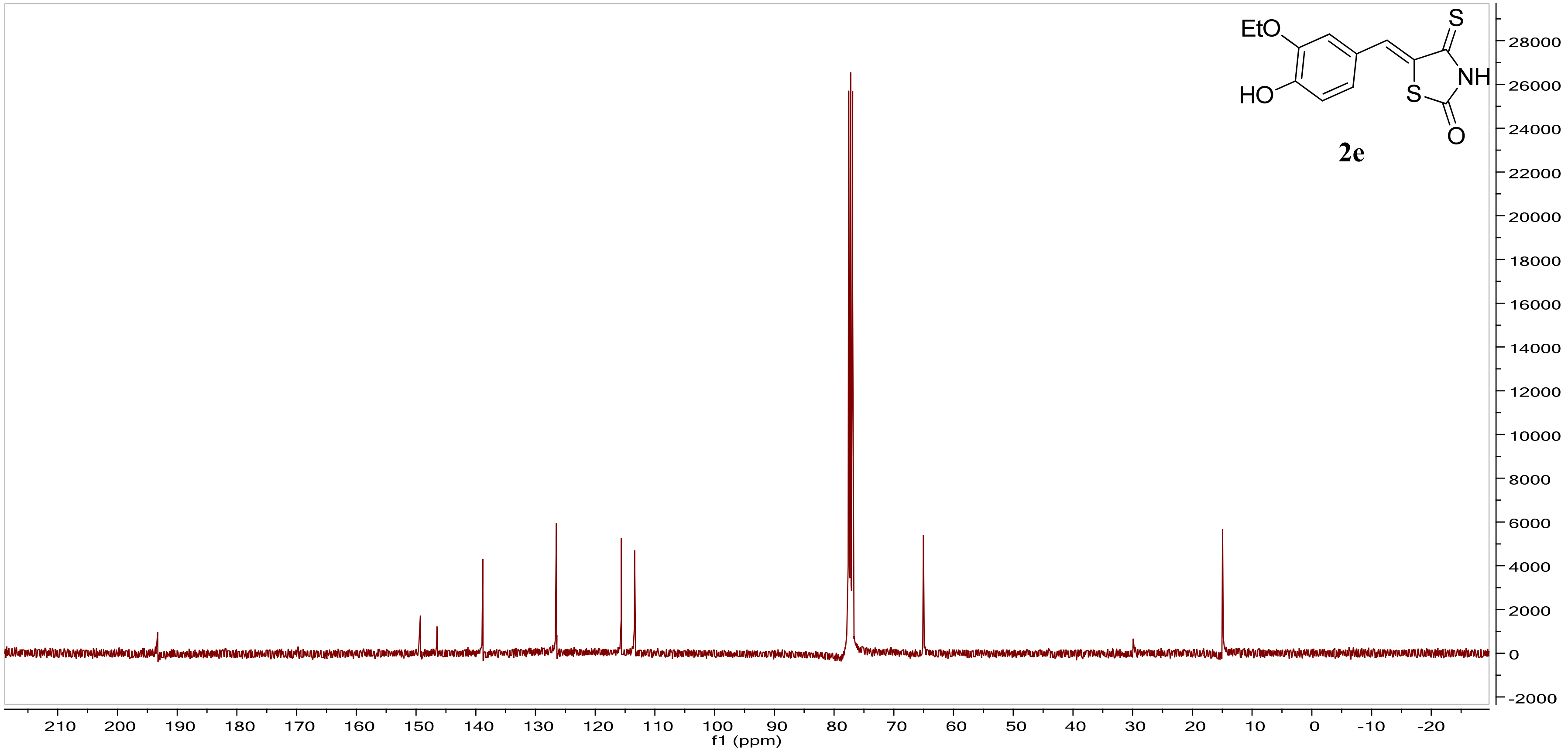

S15. ^{13}C NMR spectrum of compound **2e**

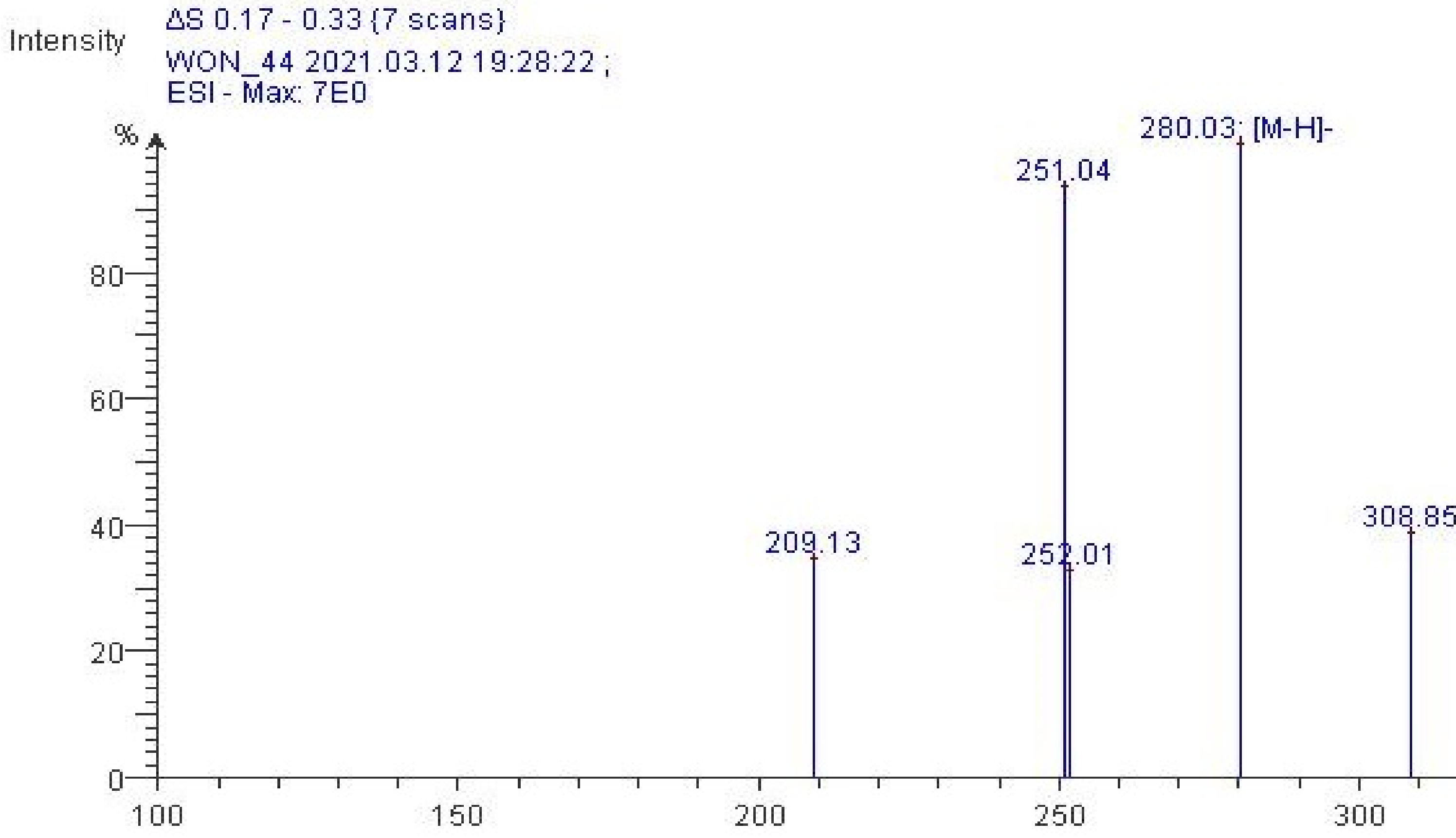

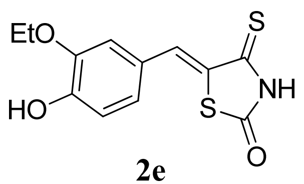

2e

S16. LRMS spectrum of compound **2e**

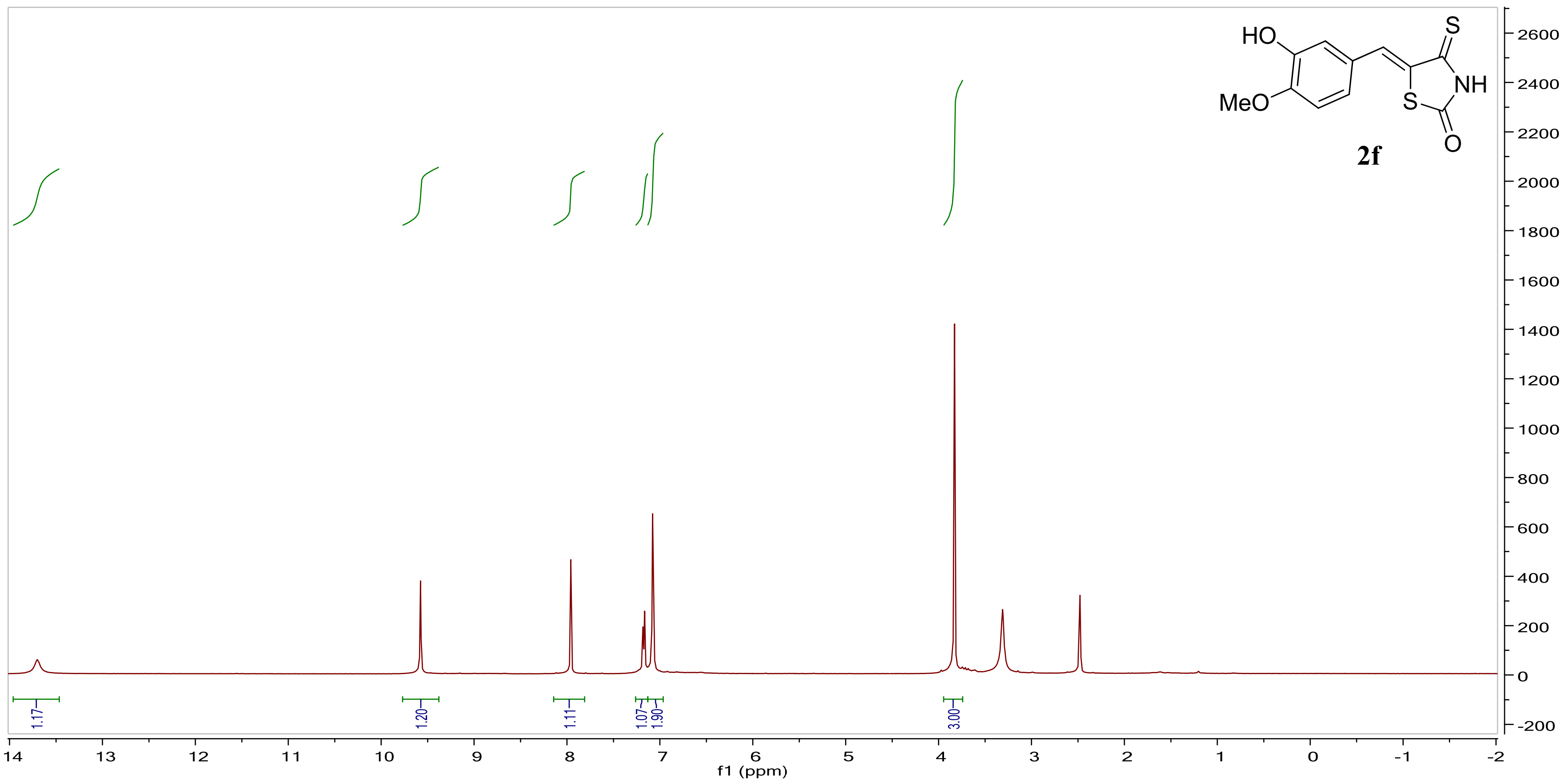

S17. ^{1}H NMR spectrum of compound **2f**

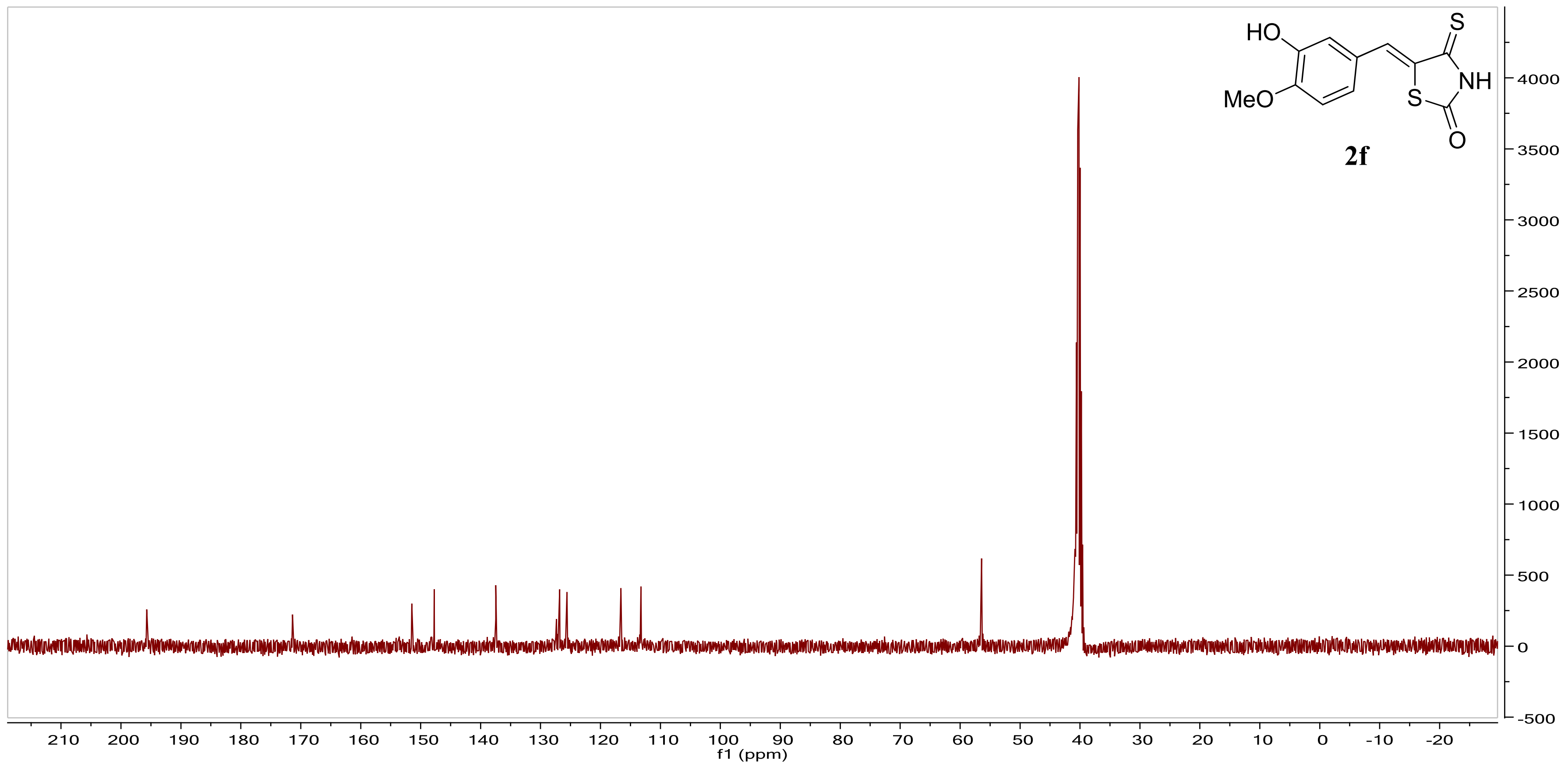

S18. ^{13}C NMR spectrum of compound **2f**

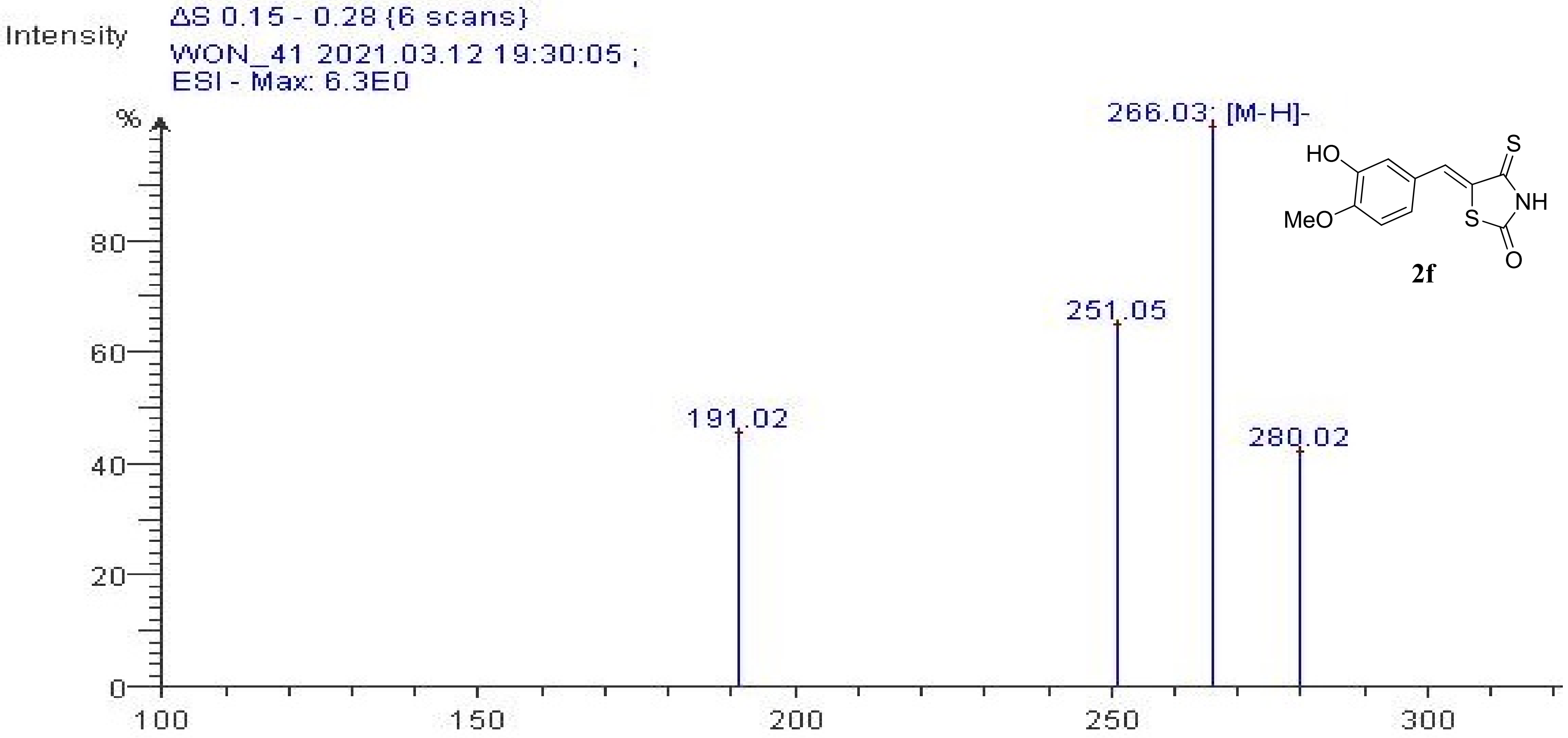

S19. LRMS spectrum of compound **2f**

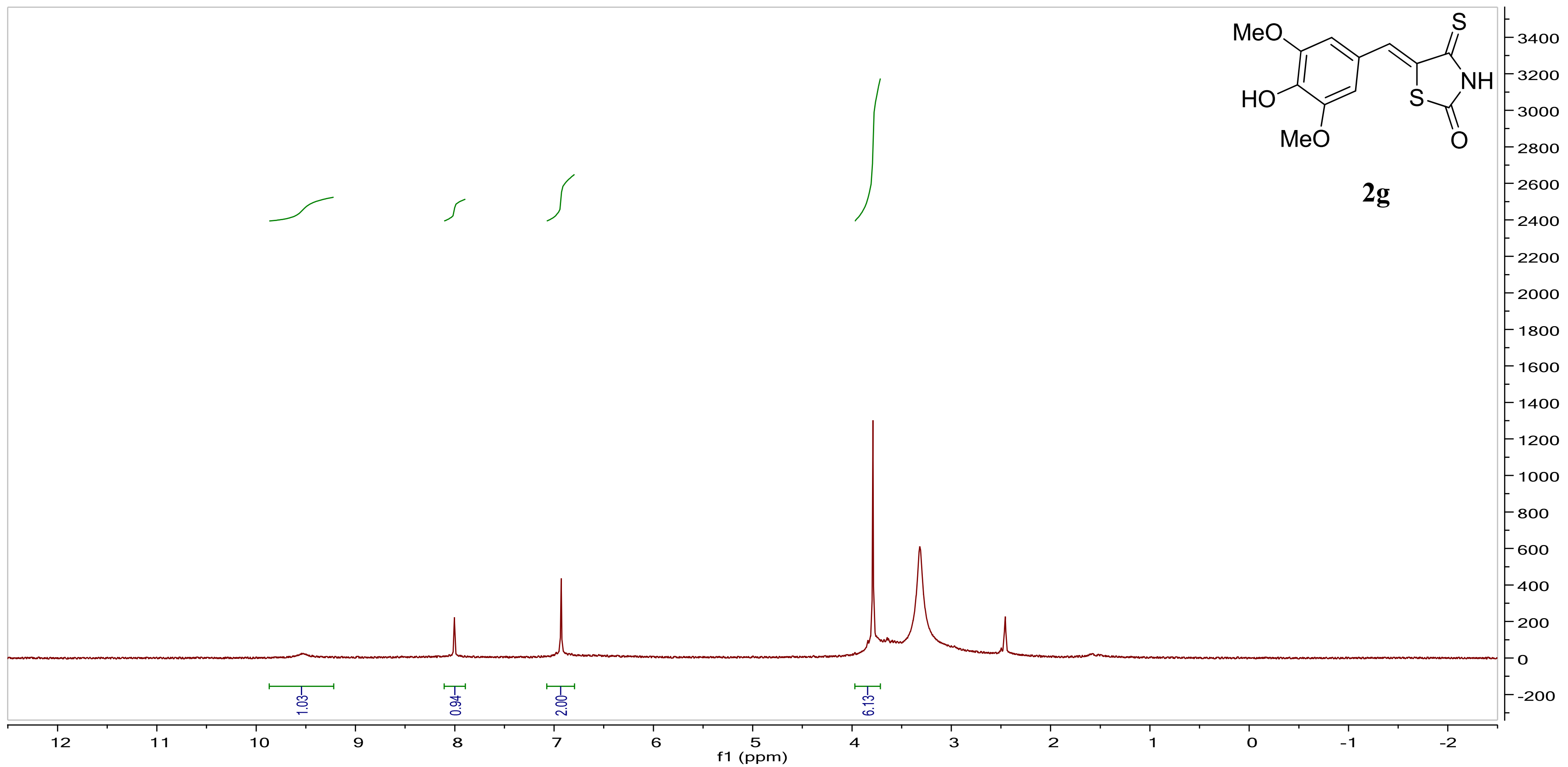

S20. ^{1}H NMR spectrum of compound **2g**

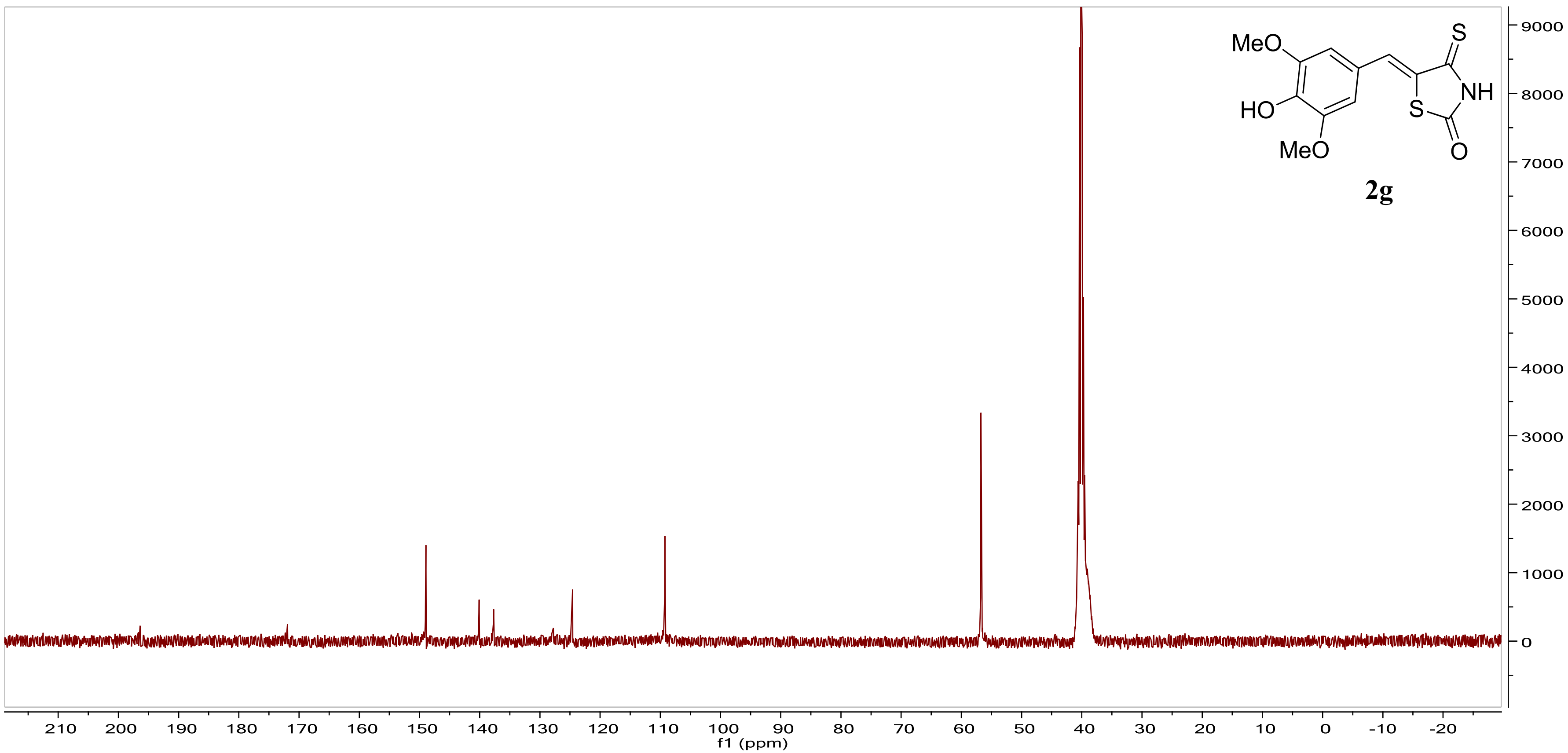

S21. ^{13}C NMR spectrum of compound **2g**

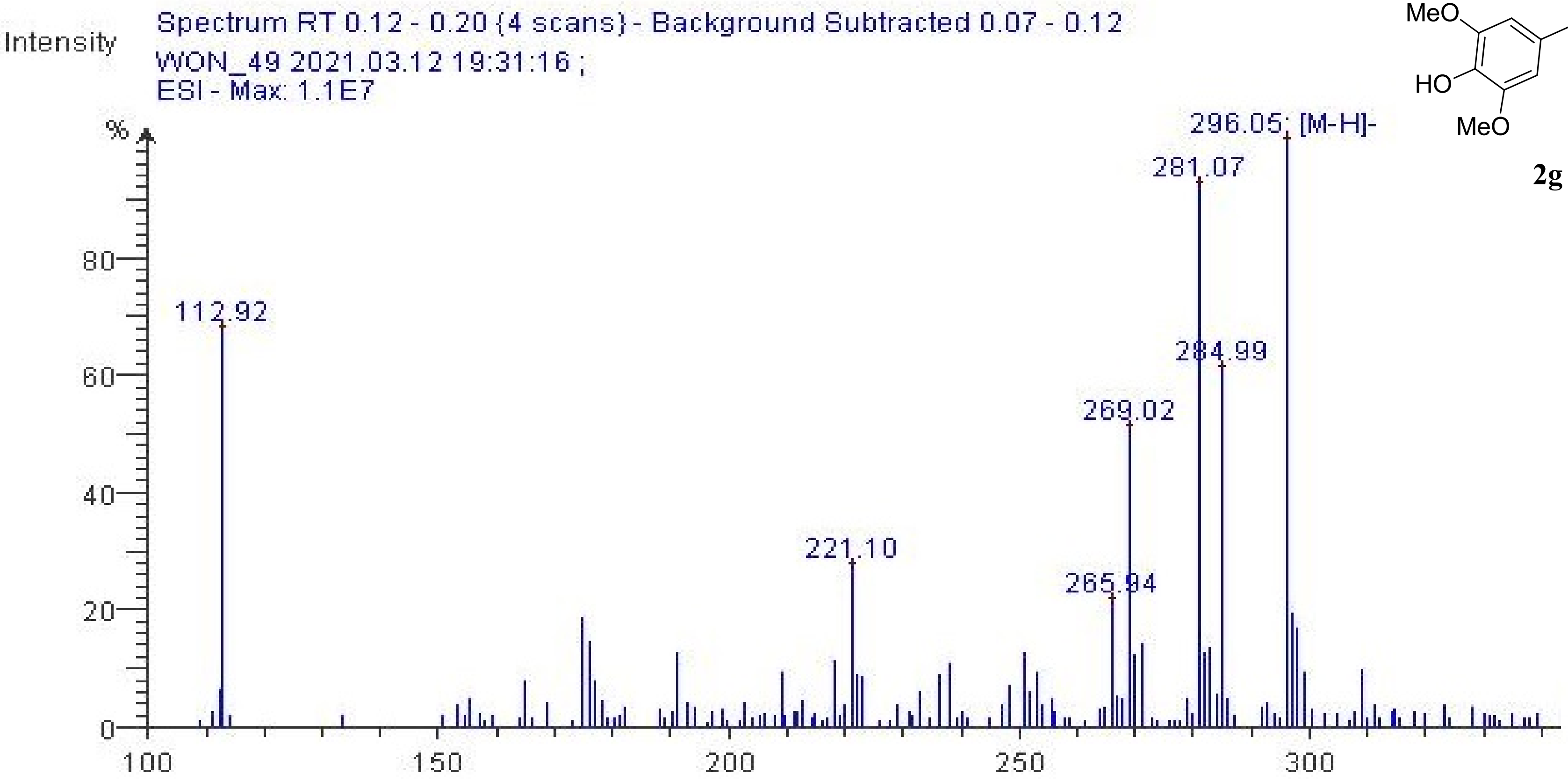

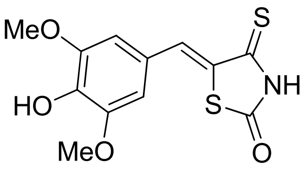

2g

S22. LRMS spectrum of compound **2g**

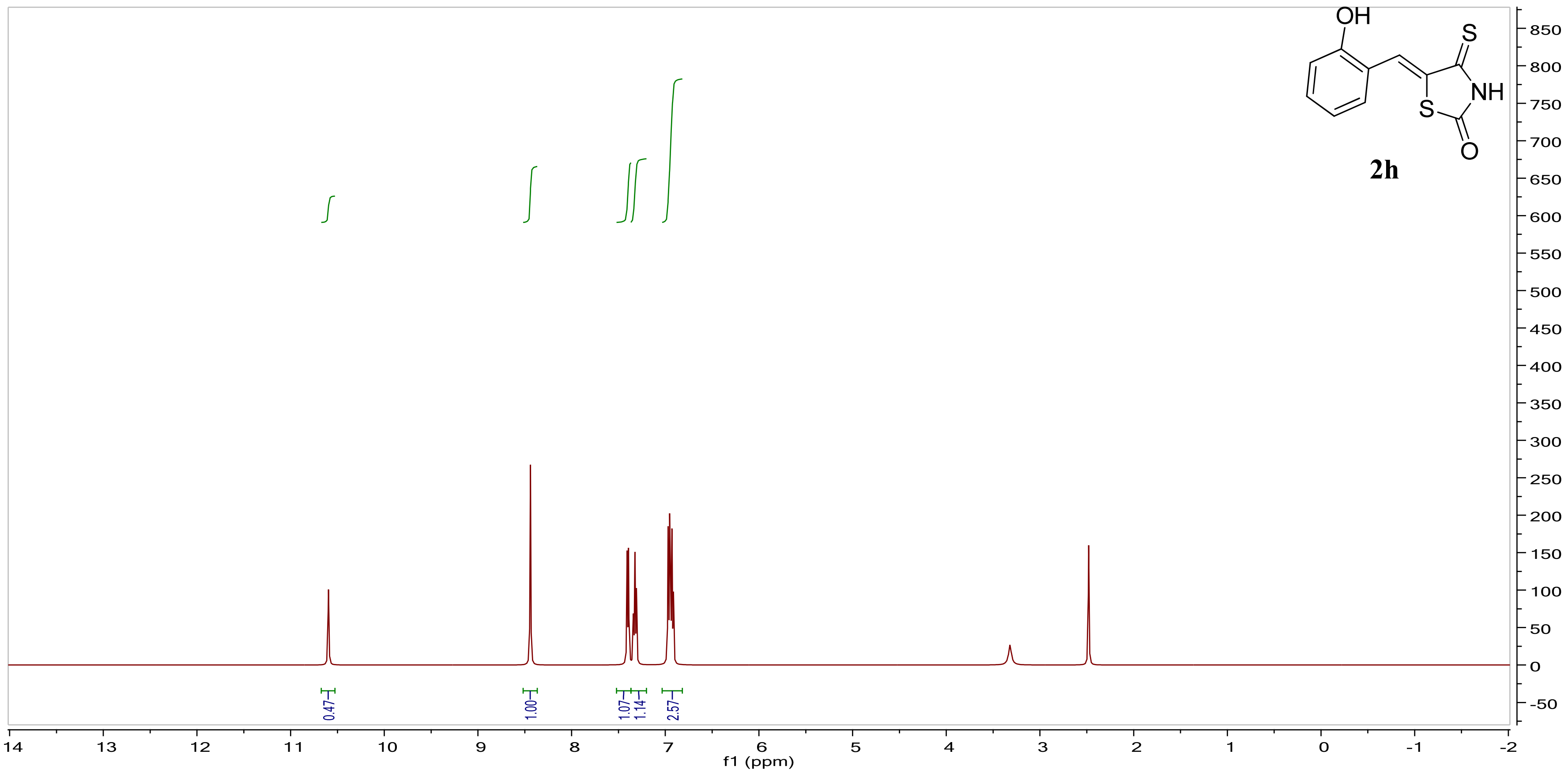

S23. ^{1}H NMR spectrum of compound **2h**

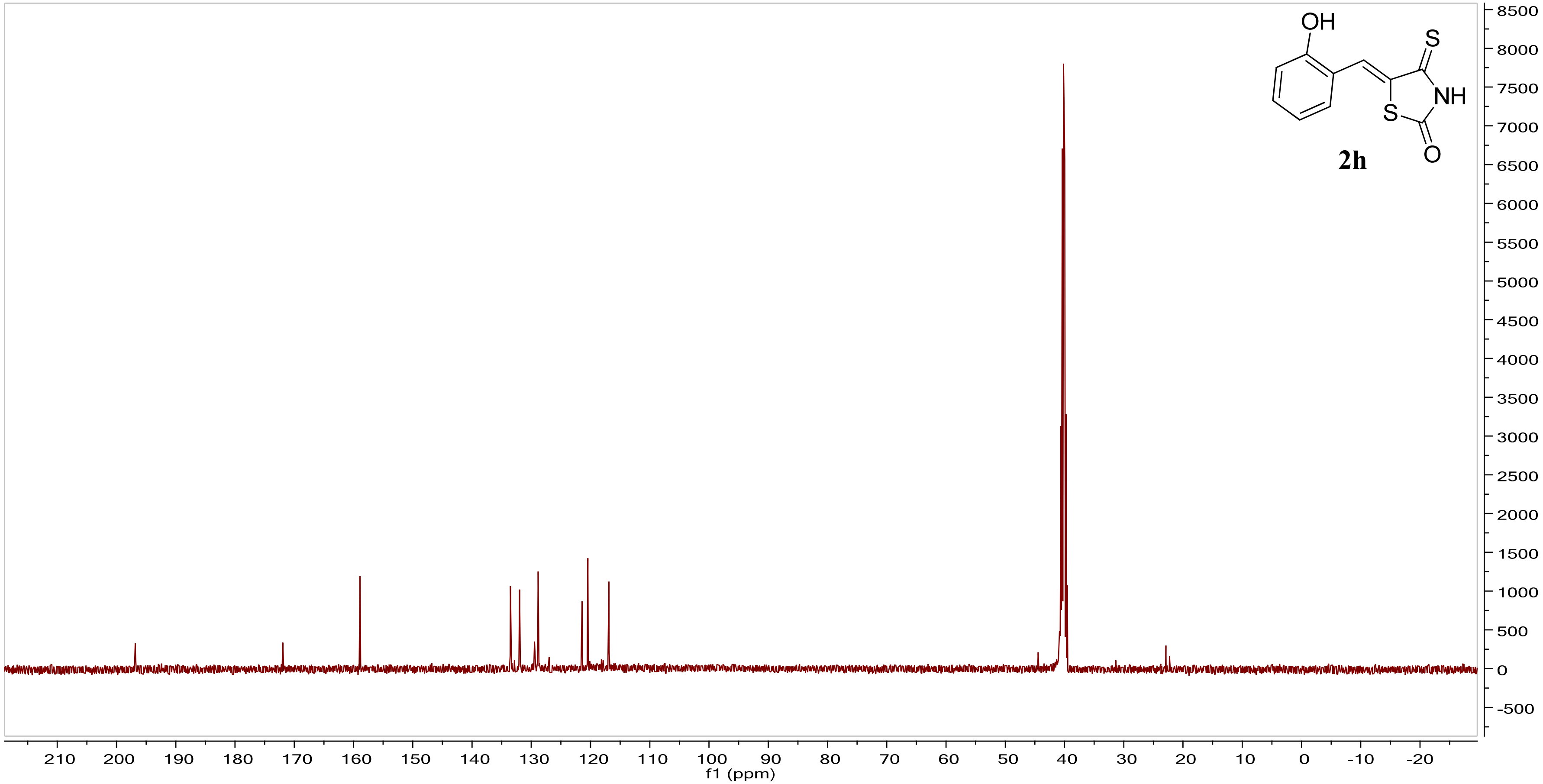

S24. ^{13}C NMR spectrum of compound **2h**

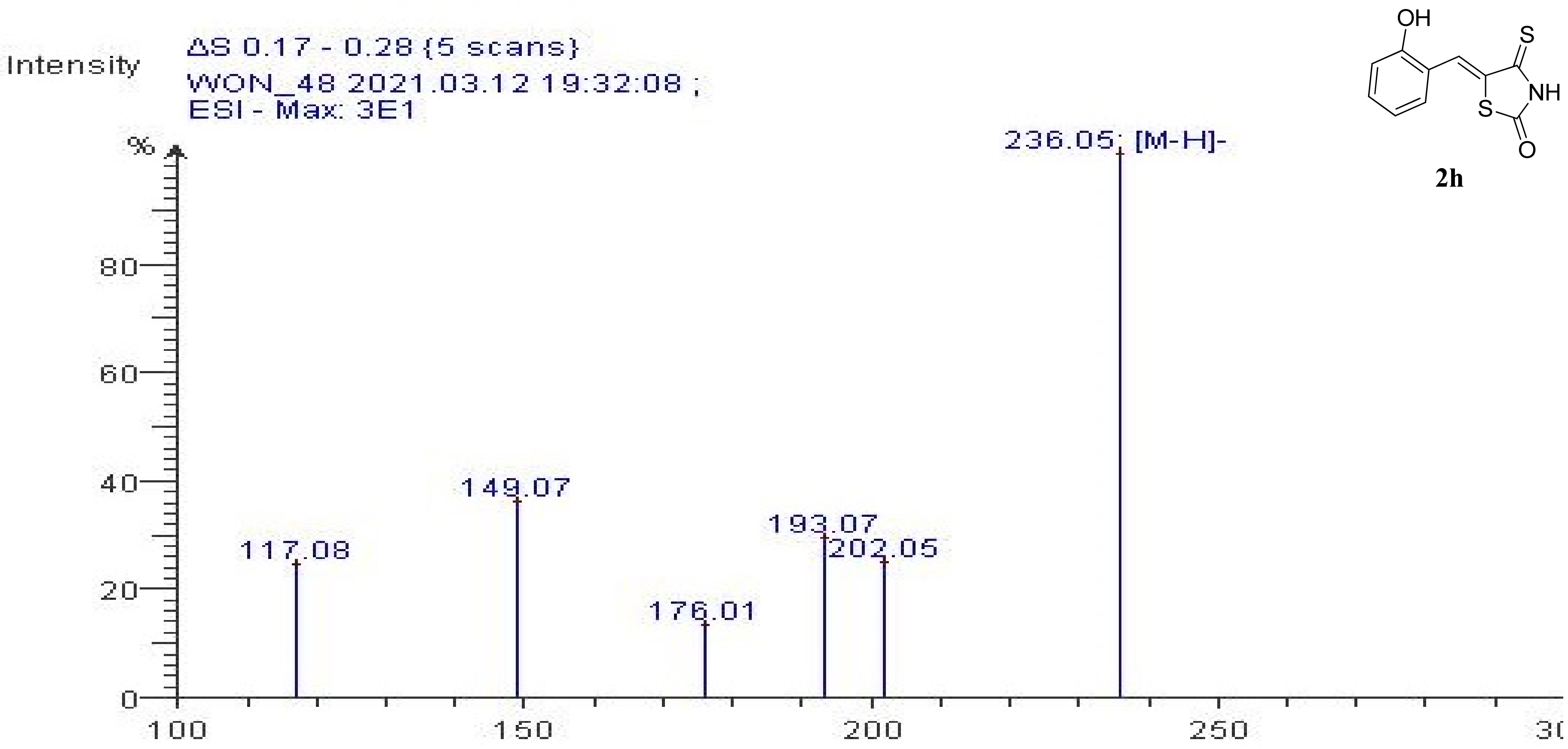

2h

S25. LRMS spectrum of compound **2h**

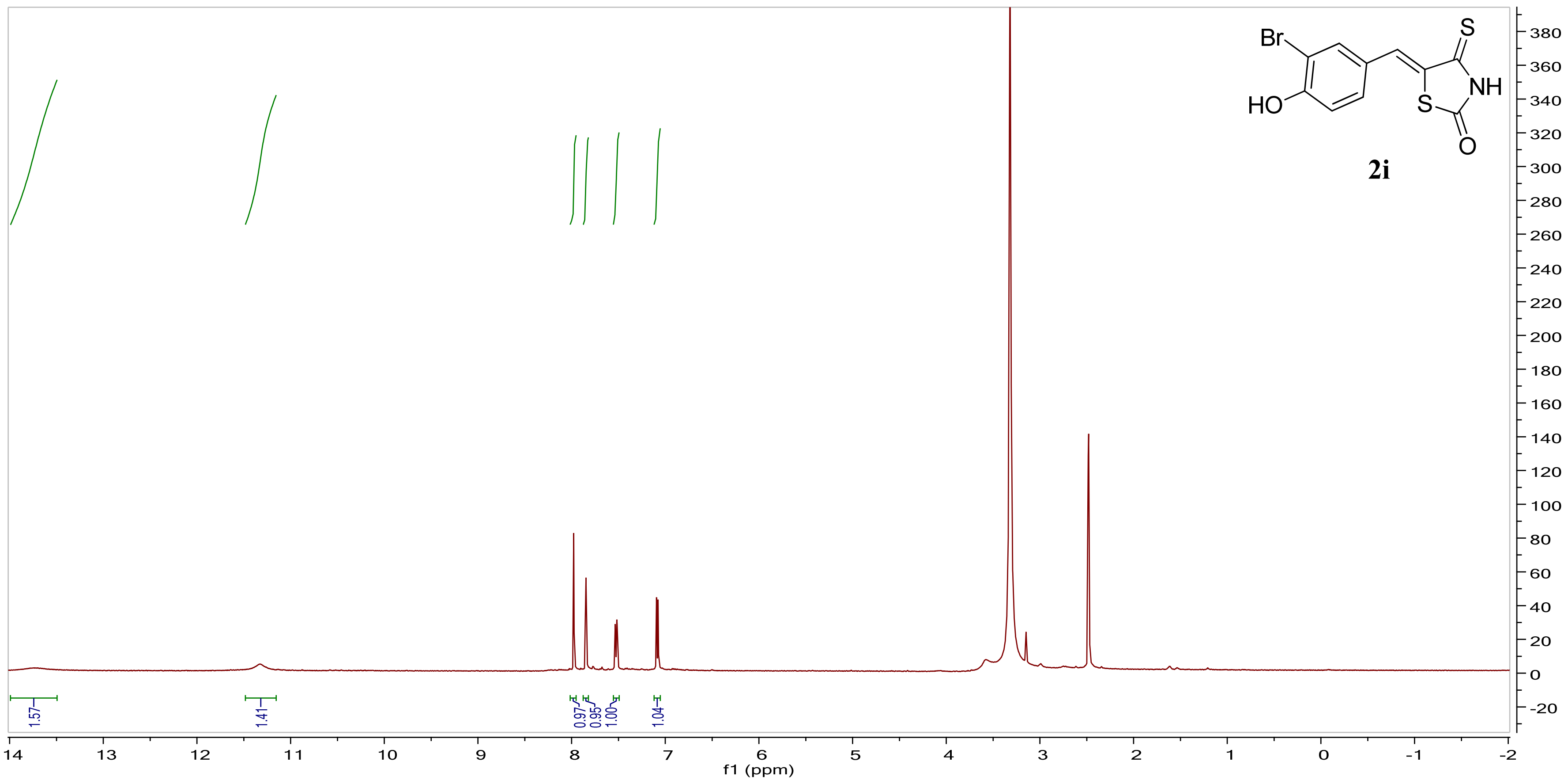

S26. ^{1}H NMR spectrum of compound **2i**

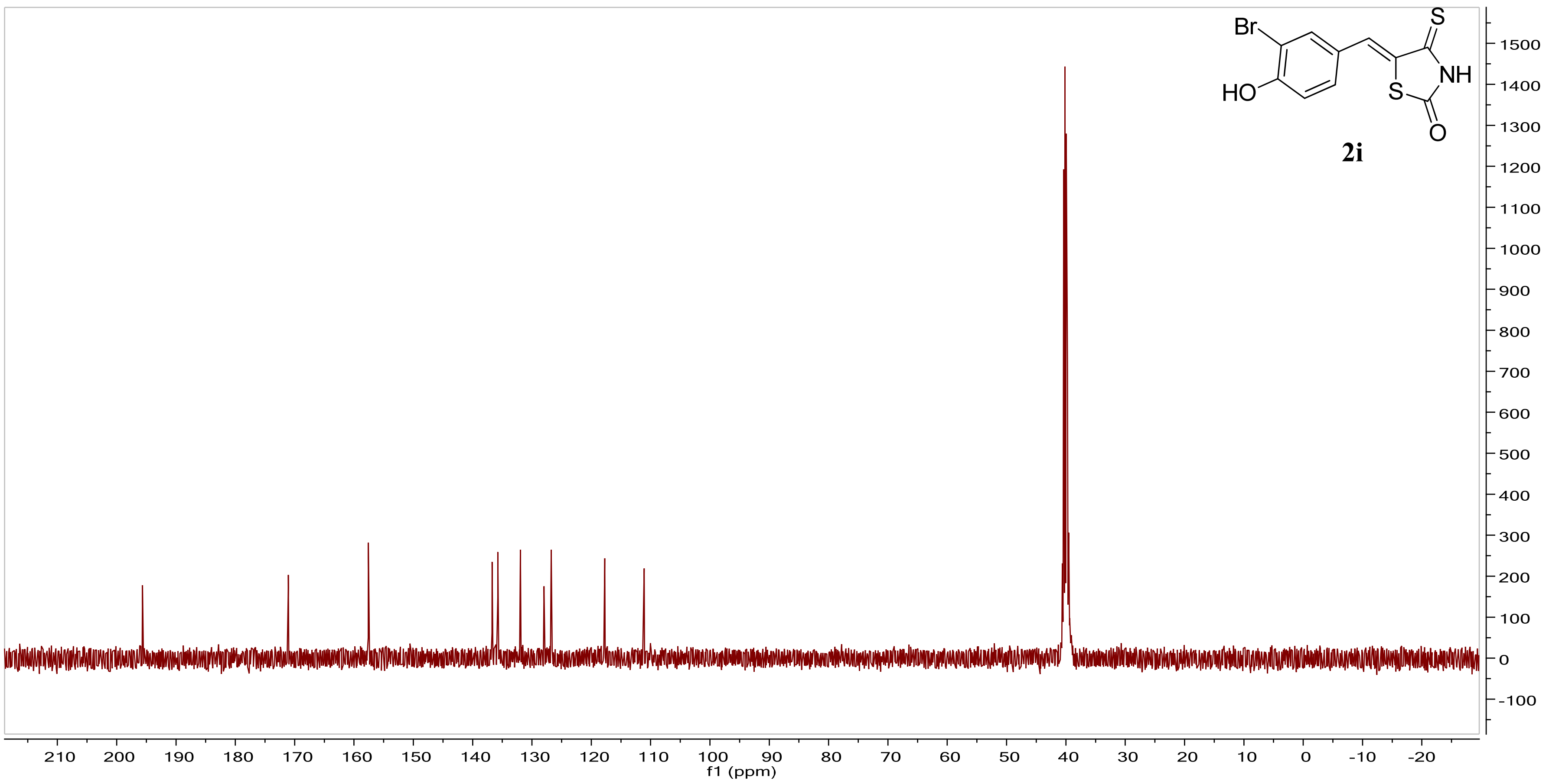

S27. ^{13}C NMR spectrum of compound **2i**

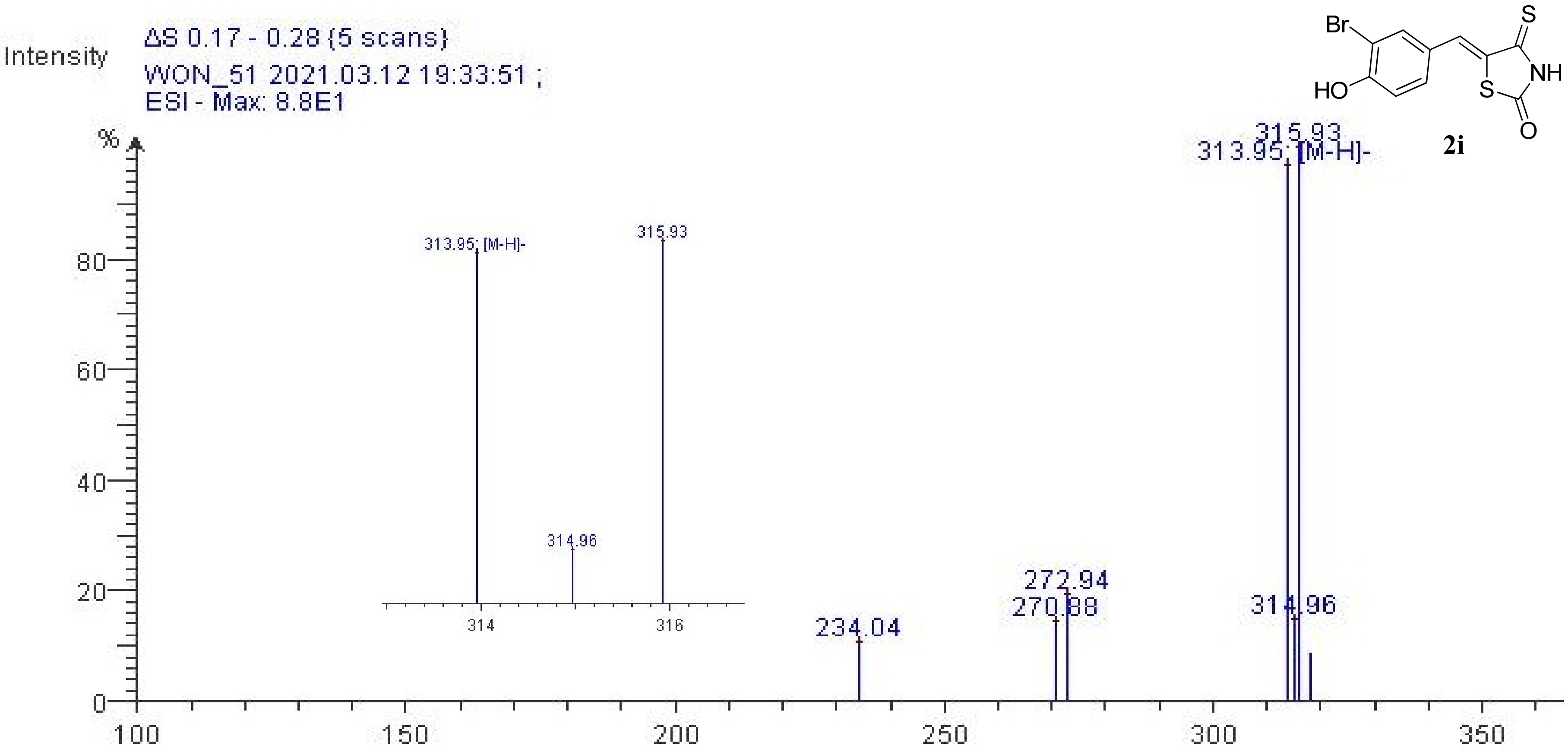

2i

S28. LRMS spectrum of compound **2i**

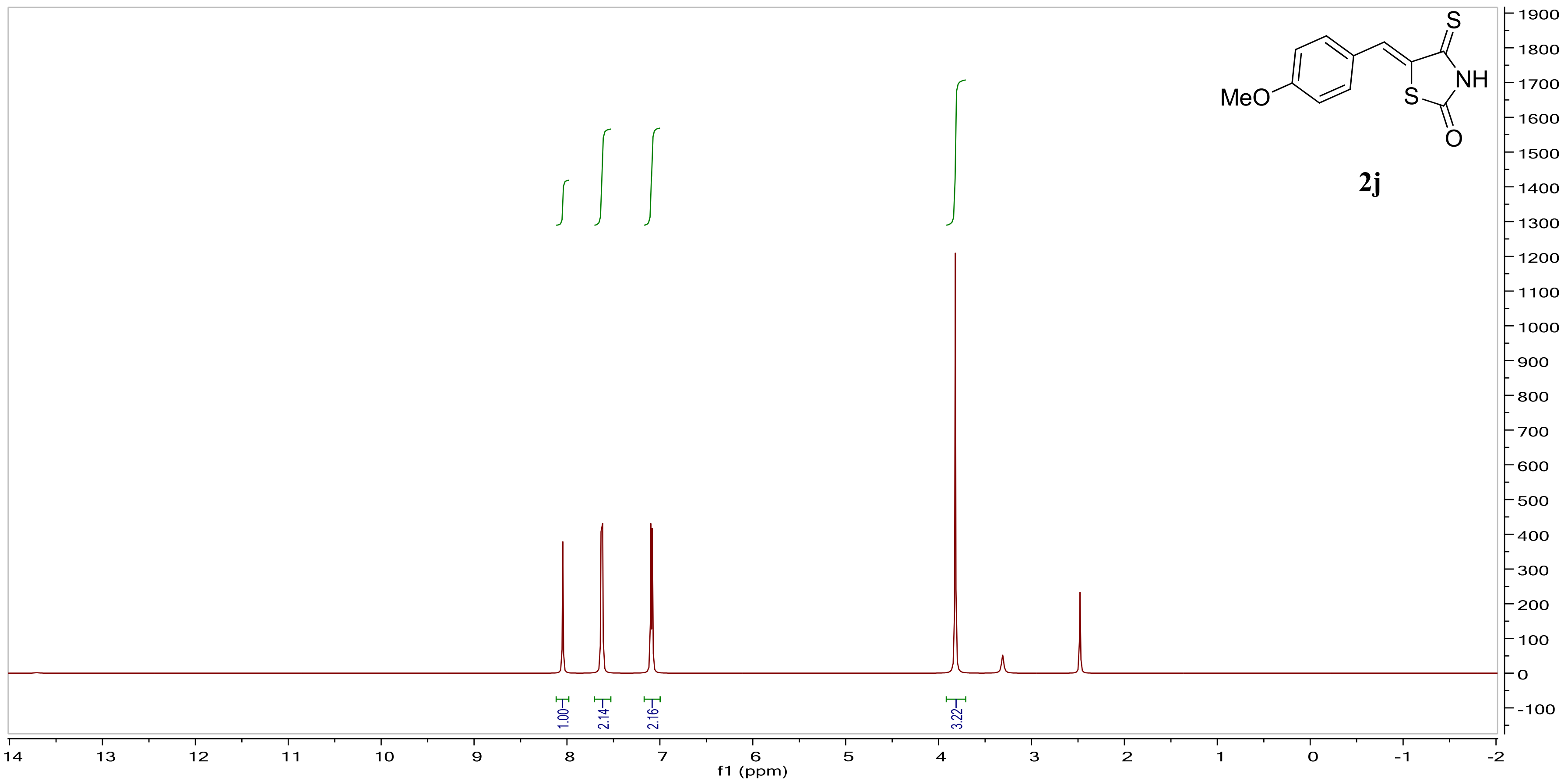

S29. ^{1}H NMR spectrum of compound **2j**

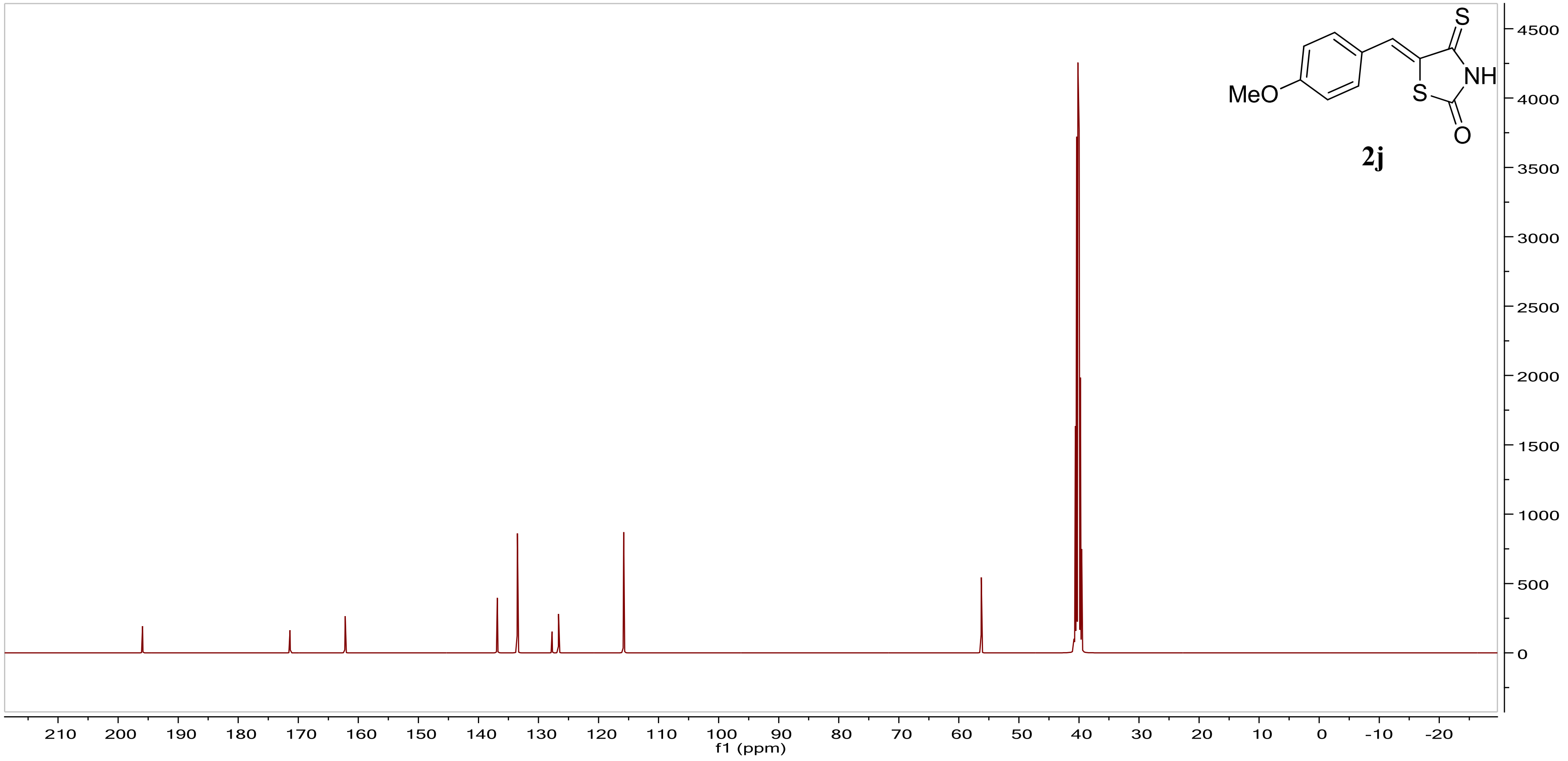

S30. ^{13}C NMR spectrum of compound **2j**

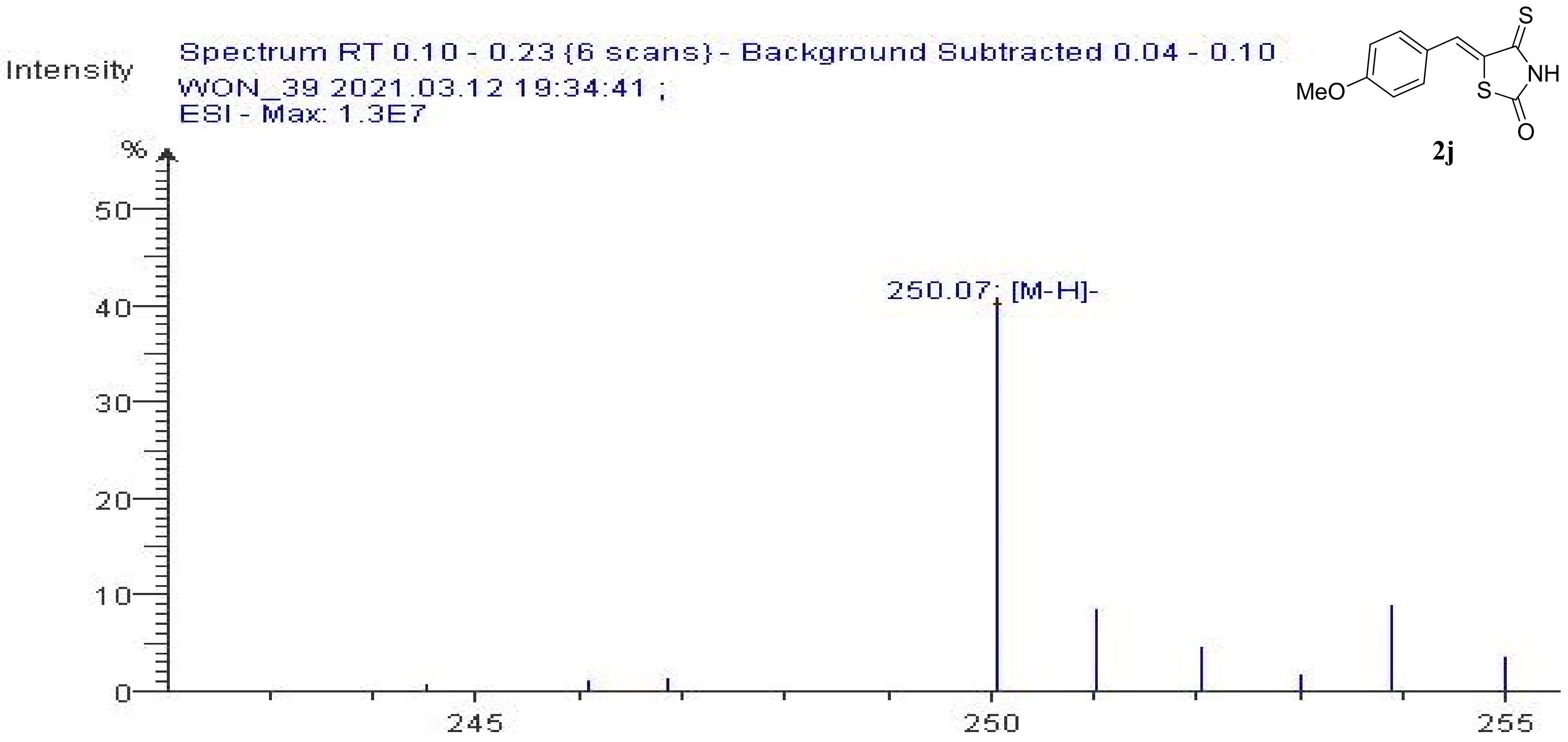

2j

S31. LRMS spectrum of compound **2j**

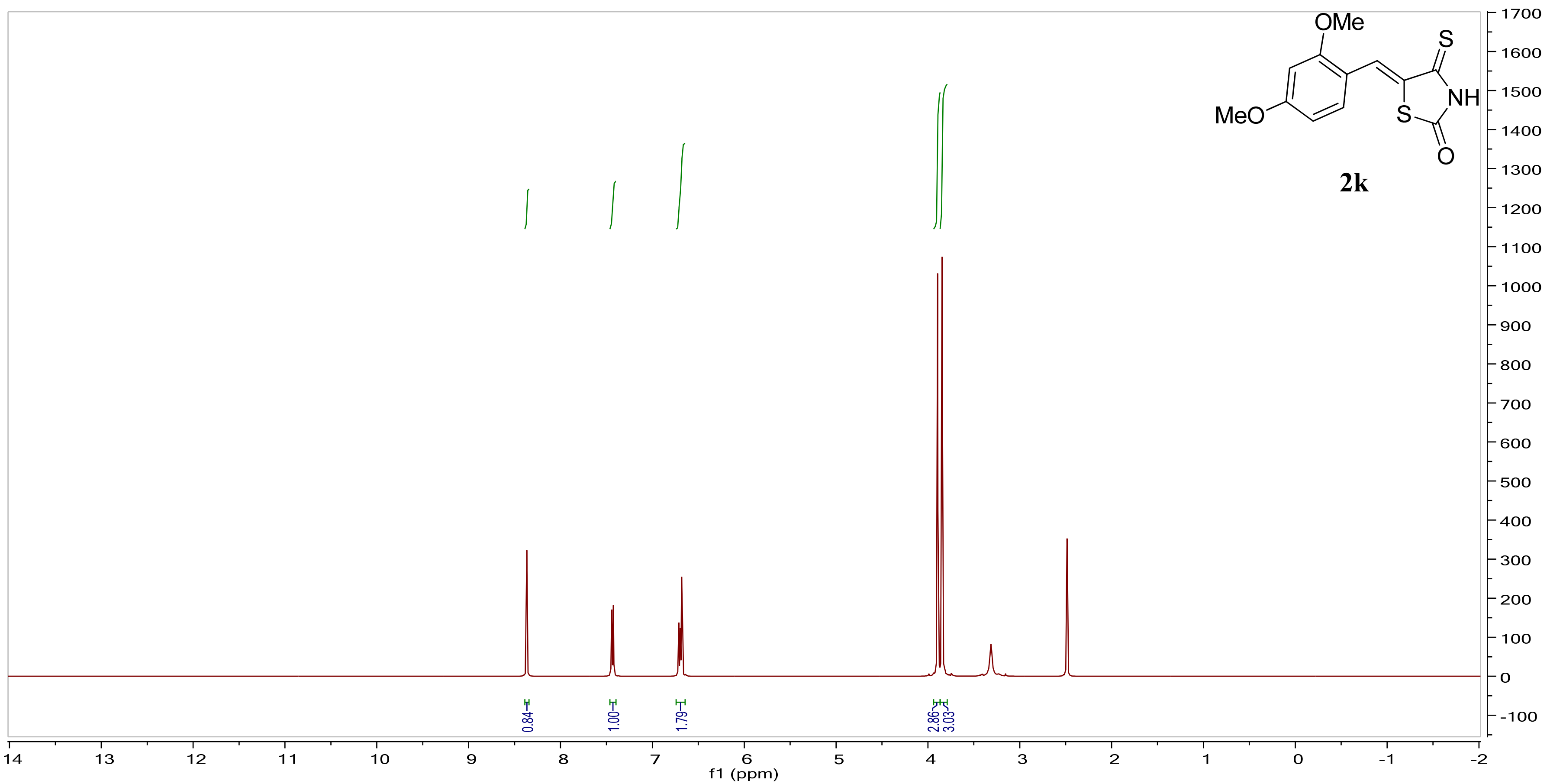

S32. ^{1}H NMR spectrum of compound **2k**

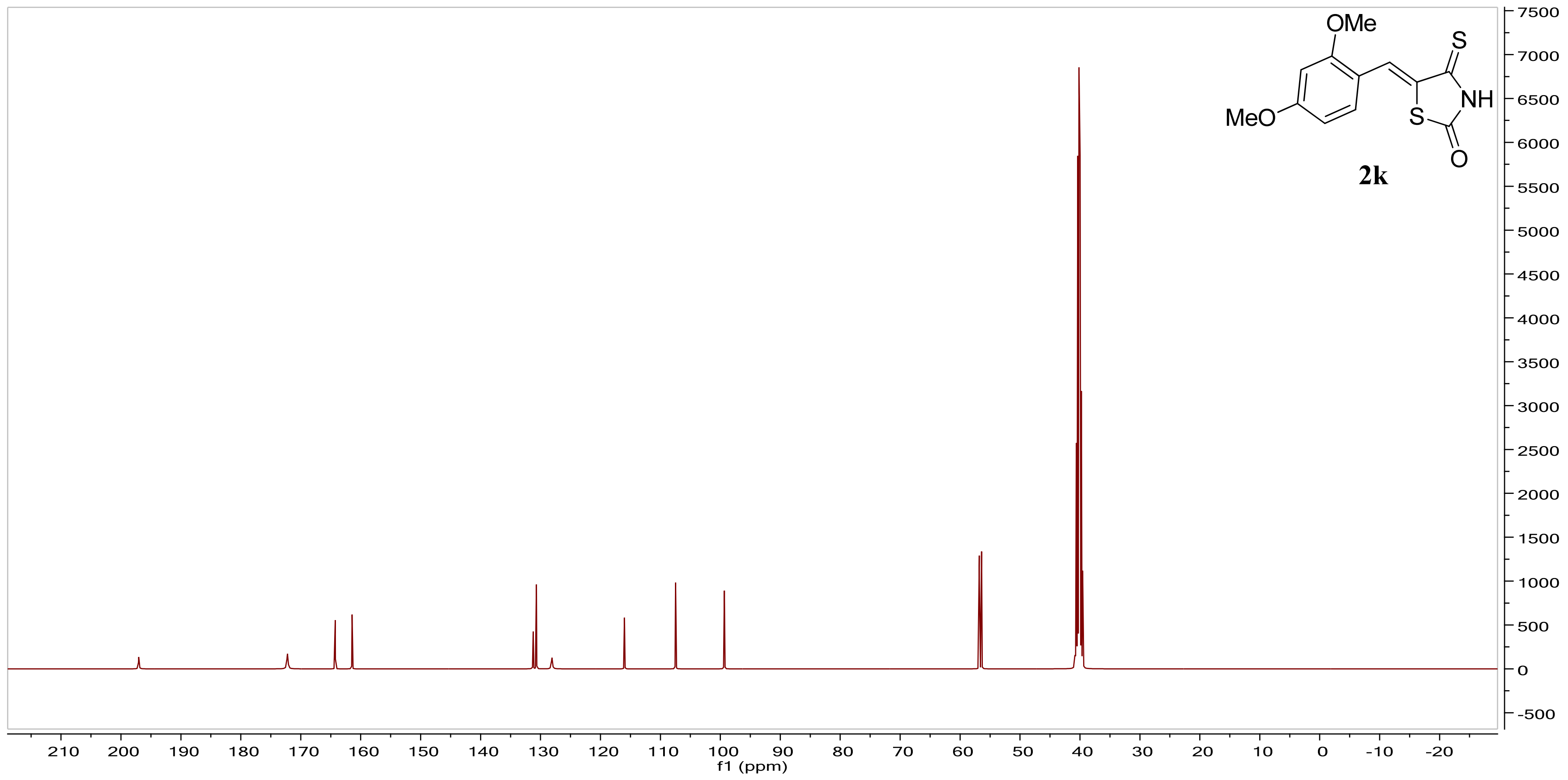

S33. ^{13}C NMR spectrum of compound **2k**

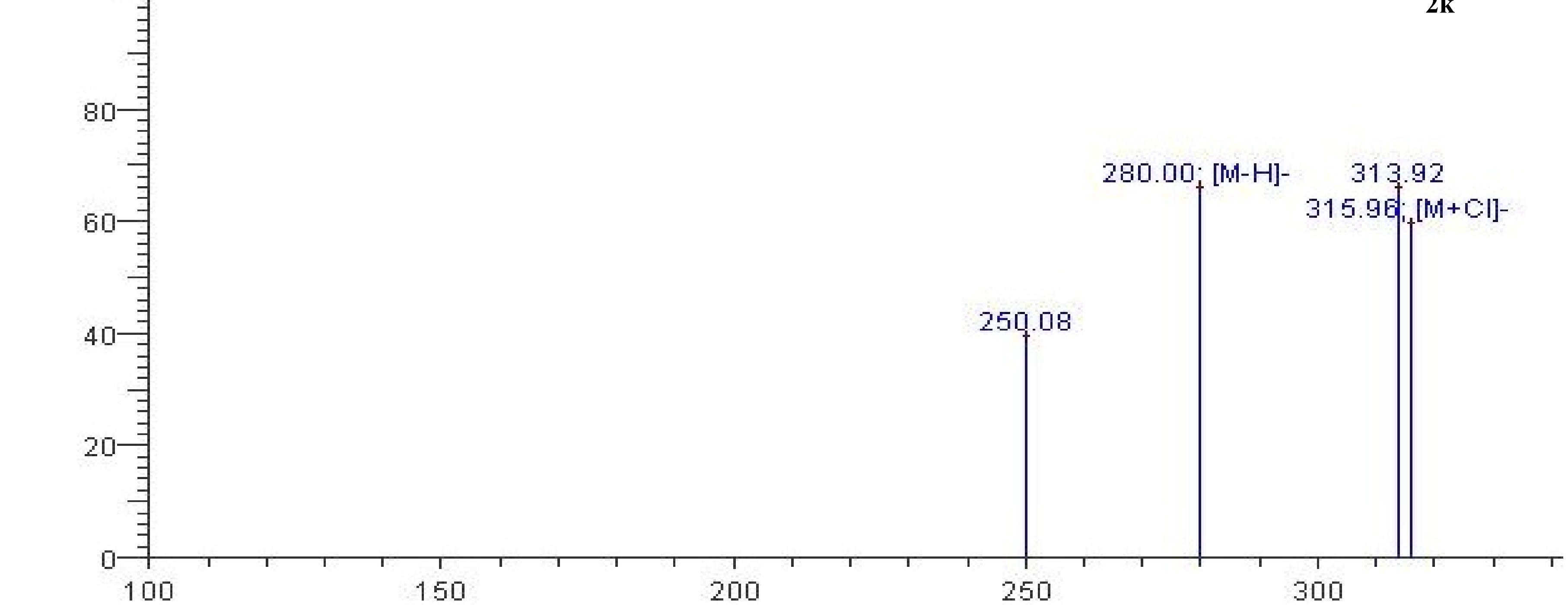

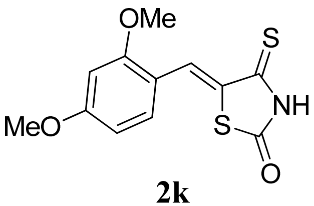

2k

S34. LRMS spectrum of compound **2k**

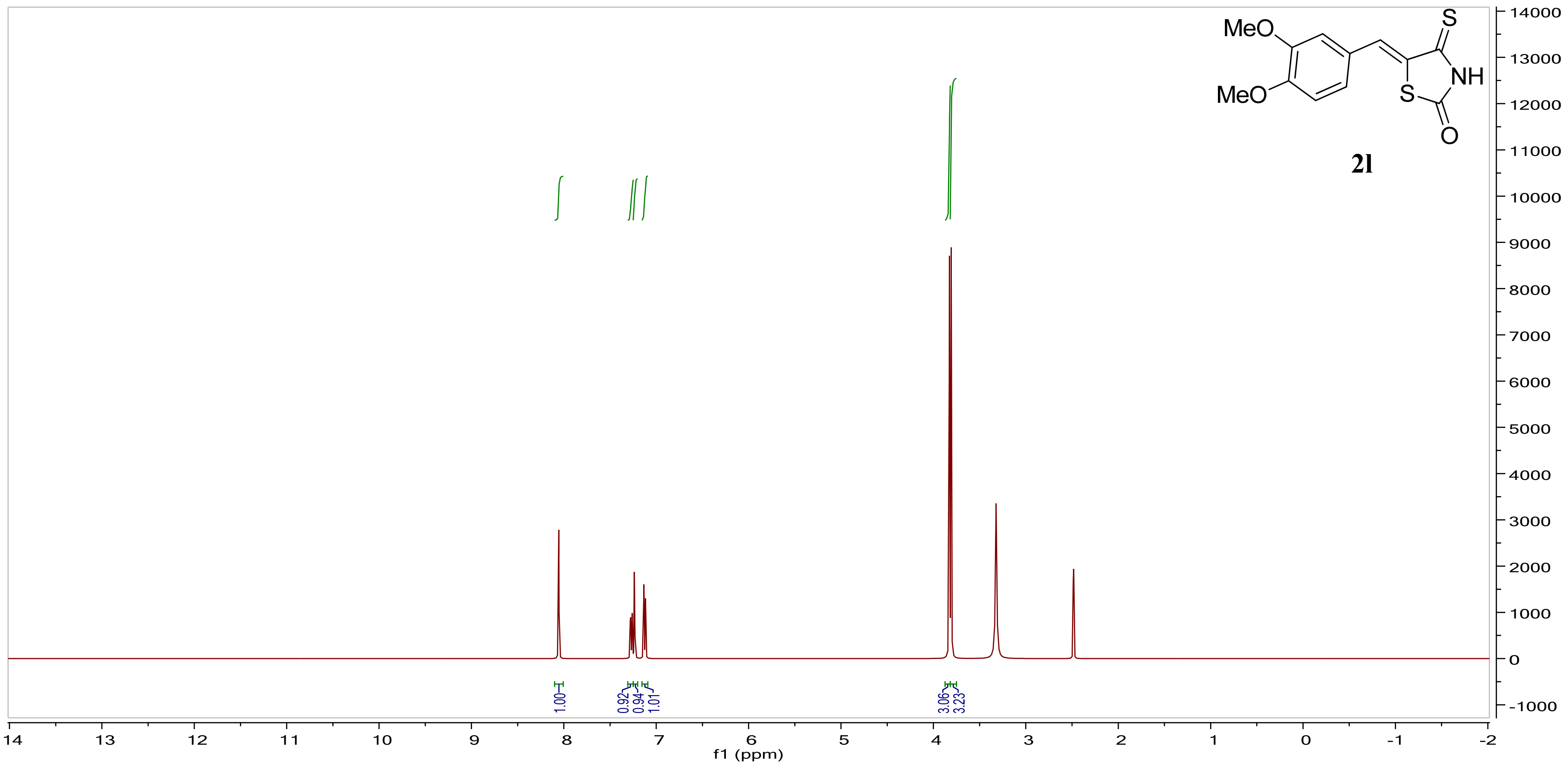

S35. ^{1}H NMR spectrum of compound **2l**

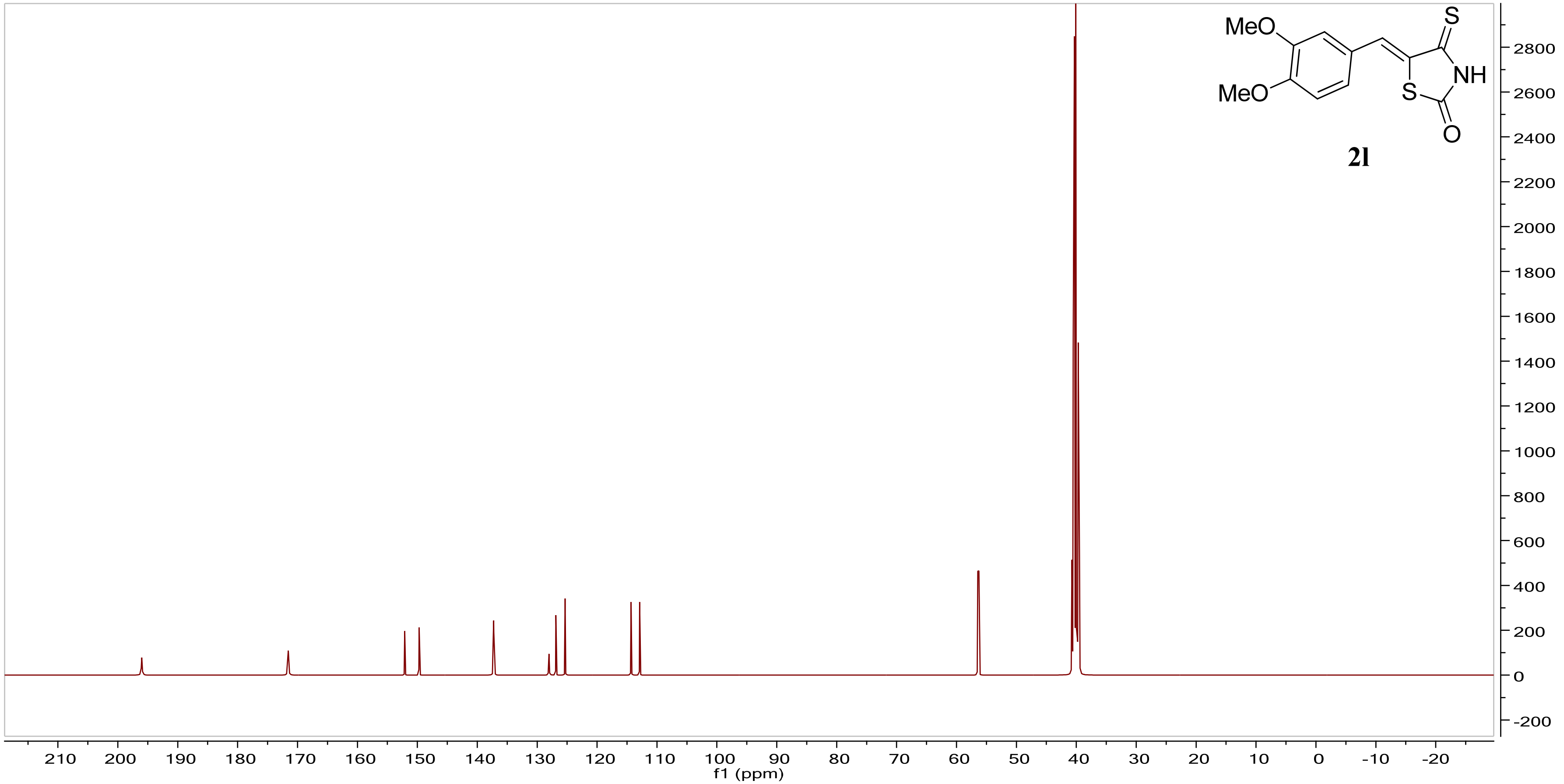

S36. ^{13}C NMR spectrum of compound **2l**

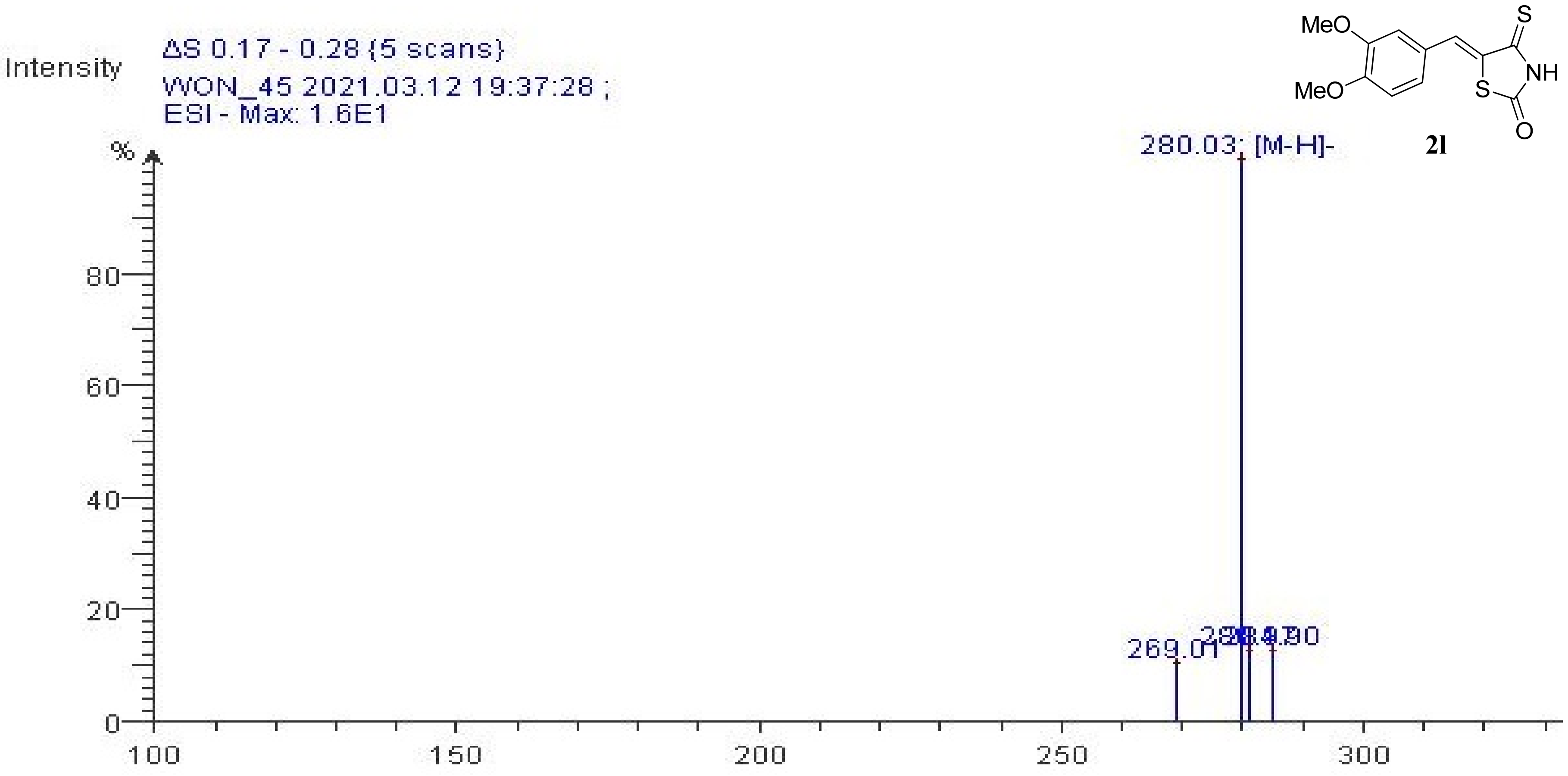

2l

S37. LRMS spectrum of compound **2l**

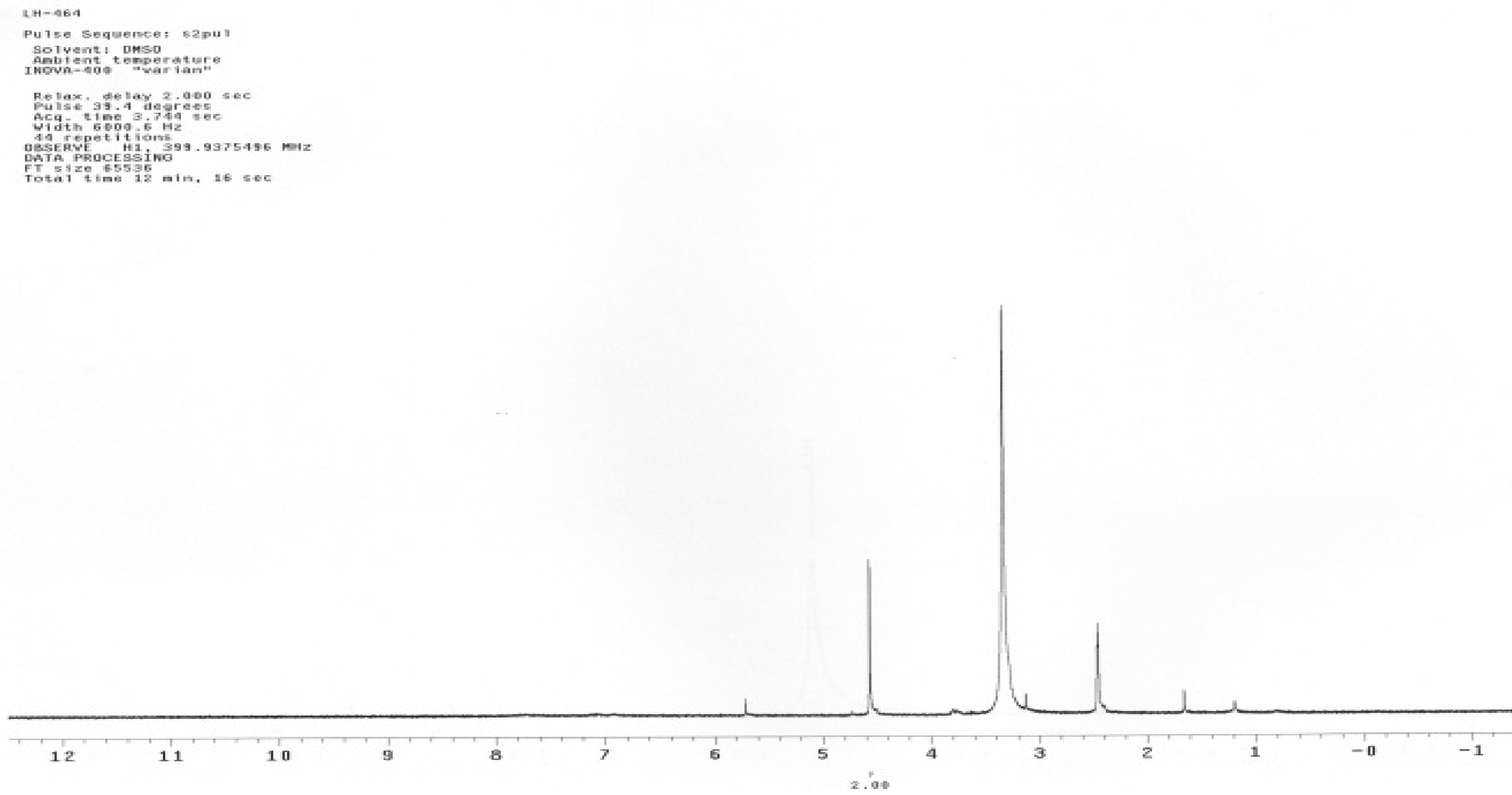

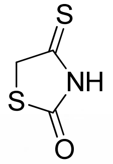

1a

S38. ^{1}H NMR spectrum of compound **1a**

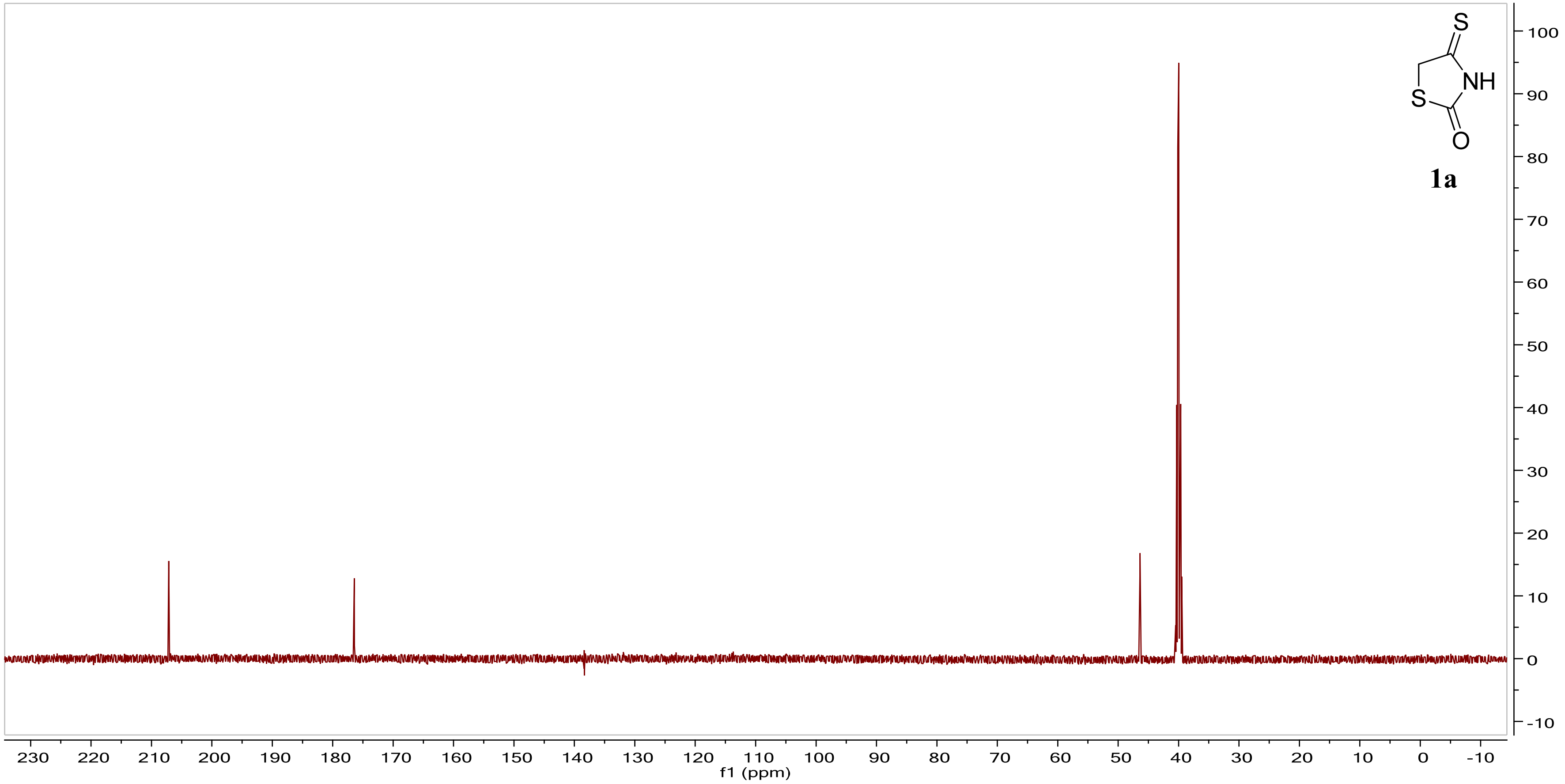

S39. ^{13}C NMR spectrum of compound **1a**

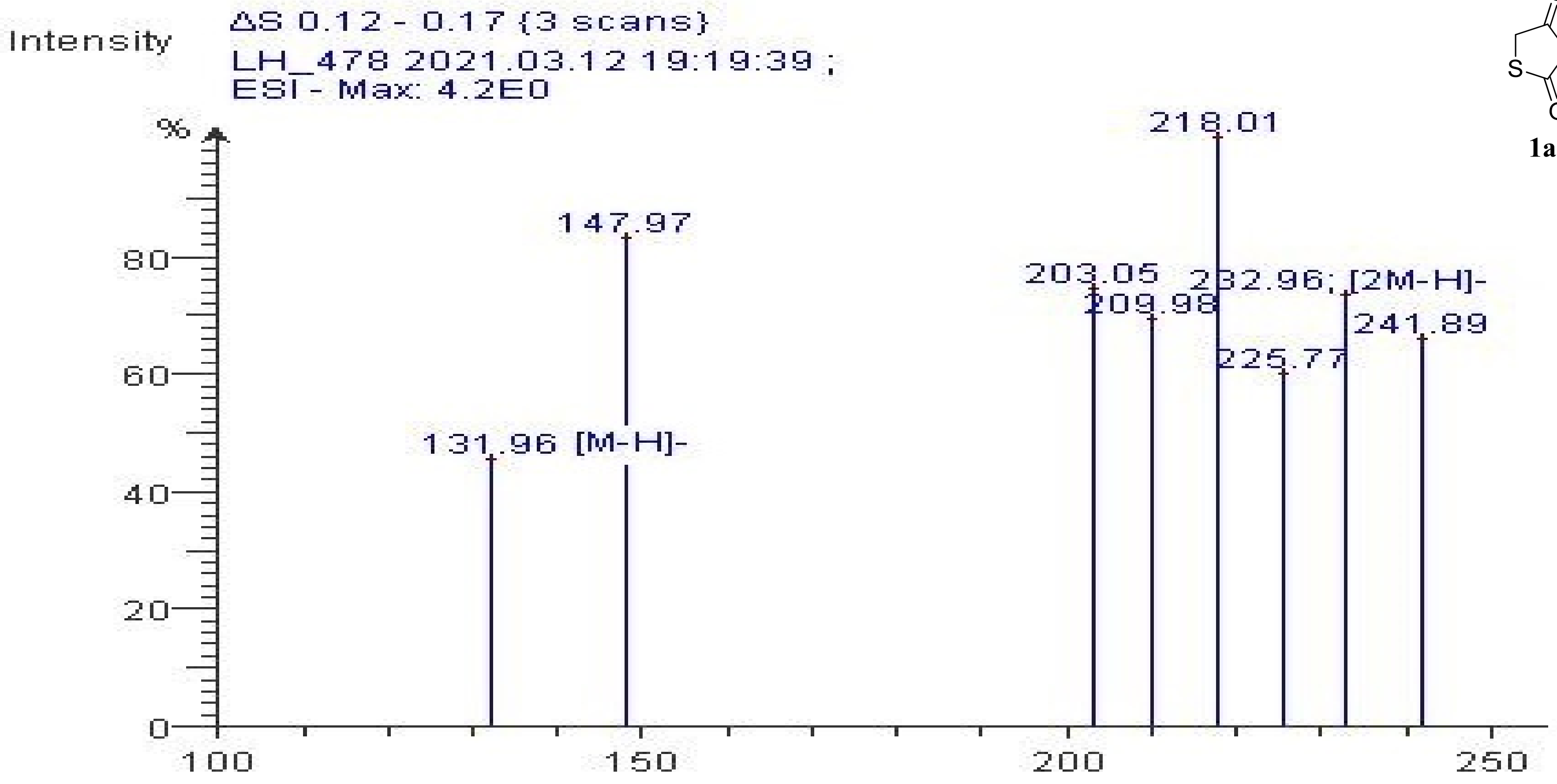

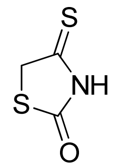

1a

S40. LRMS spectrum of compound **1a**
